# (*E*)-1-(3-(3-Hydroxy-4-Methoxyphenyl)-1-(3,4,5-Trimethoxyphenyl)allyl)-1*H*-1,2,4-Triazole and Related Compounds: Their Synthesis and Biological Evaluation as Novel Antimitotic Agents Targeting Breast Cancer

**DOI:** 10.3390/ph18010118

**Published:** 2025-01-17

**Authors:** Gloria Ana, Azizah M. Malebari, Sara Noorani, Darren Fayne, Niamh M. O’Boyle, Daniela M. Zisterer, Elisangela Flavia Pimentel, Denise Coutinho Endringer, Mary J. Meegan

**Affiliations:** 1 School of Pharmacy and Pharmaceutical Sciences, Panoz Institute, Trinity College Dublin, D02 PN40 Dublin, Ireland; 2 Department of Pharmaceutical Chemistry, College of Pharmacy, King Abdulaziz University, Jeddah 21589, Saudi Arabia; 3 Molecular Design Group, School of Chemical Sciences, Dublin City University, Glasnevin, D09 V209 Dublin, Ireland; 4 DCU Life Sciences Institute, Dublin City University, Glasnevin, D09 V209 Dublin, Ireland; 5 School of Biochemistry and Immunology, Trinity Biomedical Sciences Institute, Trinity College Dublin, 152-160 Pearse Street, D02 R590 Dublin, Ireland; 6 Department of Pharmaceutical Sciences, University Vila Velha, Av. Comissário José Dantas de Melo, n°21, Boa Vista, Vila Velha CEP 29102-920, Brazil

**Keywords:** breast cancer, tubulin polymerization inhibitor, hybrid molecule, dual targeting molecule, 1,2,4-triazole, aromatase inhibitor

## Abstract

**Background/Objectives:** The synthesis of (*E*)-1-(1,3-diphenylallyl)-1*H*-1,2,4-triazoles and related compounds as anti-mitotic agents with activity in breast cancer was investigated. These compounds were designed as hybrids of the microtubule-targeting chalcones, indanones, and the aromatase inhibitor letrozole. **Methods**: A panel of 29 compounds was synthesized and examined by a preliminary screening in estrogen receptor (ER) and progesterone receptor (PR)-positive MCF-7 breast cancer cells together with cell cycle analysis and tubulin polymerization inhibition. **Results**: (*E*)-5-(3-(1*H*-1,2,4-triazol-1-yl)-3-(3,4,5-trimethoxyphenyl)prop-1-en-1-yl)-2-methoxyphenol **22b** was identified as a potent antiproliferative compound with an IC_50_ value of 0.39 mM in MCF-7 breast cancer cells, 0.77 mM in triple-negative MDA-MB-231 breast cancer cells, and 0.37 mM in leukemia HL-60 cells. In addition, compound **22b** demonstrated potent activity in the sub-micromolar range against the NCI 60 cancer cell line panel including prostate, melanoma, colon, leukemia, and non-small cell lung cancers. G_2_/M phase cell cycle arrest and the induction of apoptosis in MCF-7 cells together with inhibition of tubulin polymerization were demonstrated. Immunofluorescence studies confirmed that compound **22b** targeted tubulin in MCF-7 cells, while computational docking studies predicted binding conformations for **22b** in the colchicine binding site of tubulin. Compound **22b** also selectively inhibited aromatase. **Conclusions**: Based on the results obtained, these novel compounds are suitable candidates for further investigation as antiproliferative microtubule-targeting agents for breast cancer.

## 1. Introduction

**Figure 1:**
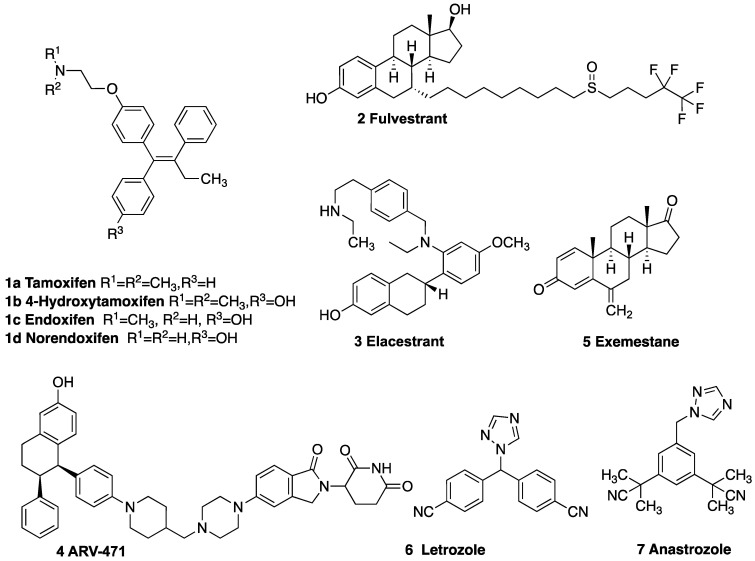
Drugs for the treatment of breast cancer: SERMs (tamoxifen **1a**, 4-hydroxytamoxifen **1b**, endoxifen **1c**, norendoxifen **1d**), SERD fulvestrant **2**, PROTAC elacestrant **3**, ARV-471 **4**, aromatase inhibitors (exemestane **5**, letrozole **6**, and anastrozole **7**).

Breast cancer (BC) is one of the leading causes of cancer-related deaths in women. One in nine women will develop breast cancer in the course of their lifetime with 609,820 breast cancer deaths projected for the US in 2023 [1]. BC is the second leading cause of mortality in Europe and the United States with an incident rate of about 2.6 million cases per year; 0.5–1% of BCs diagnosed occur in men [1]. Drugs commonly used for BC chemotherapy include topisomerase I/II inhibitors (anthracyclines doxorubicin and epirubicin), taxanes (paclitaxel and docetaxel), antimetabolites (e.g., 5-fluorouracil, capecitabine), platinum salts (carboplatin), and alkylating agents (cyclophosphamide) [2]. Approximately 70% of BCs are estrogen receptor α (ERα) positive (ER+). The selective estrogen receptor modulator (SERM) tamoxifen **1a** with antiestrogen action in breast cells is the most commonly used drug in endocrine therapy for ER + BC [3]; the related metabolites 4-hydroxytamoxifen **1b**, endoxifen **1c,** and norendoxifen **1d** also demonstrate potent antiestrogenic activity (Figure 1). However, BC cells can easily develop resistance to tamoxifen [4] while side-effects include the increased risk of endometrial cancer [5] and liver abnormalities [6]. Fulvestrant **2** and elacestrant **3** act as selective estrogen receptor degraders (SERD) [7], while selective estrogen receptor covalent antagonists (SERCAs) [8] and proteolysis targeting chimerics (PROTACs) such as ARV-471 **4** are in clinical development [9,10] (Figure 1).

**Figure 2:**
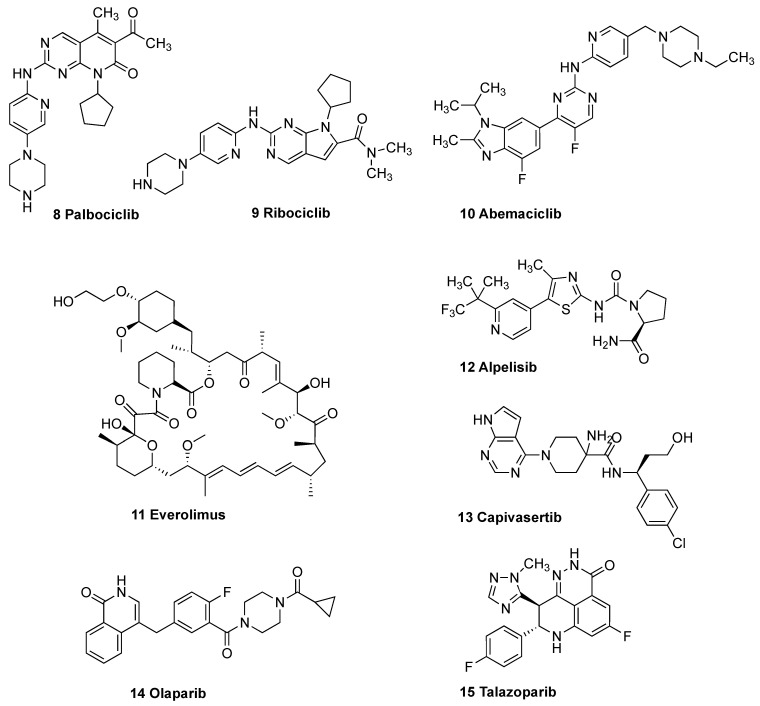
Targeted therapies for breast cancer: CDK4/6 inhibitors palbociclib **8**, ribociclib **9**, and abemacicilib **10**, mTOR inhibitor everolimus **11**; PI3K inhibitor alpelisib **12**, AKT inhibitor capivasertib **13**; PARP inhibitors olaparib **14**, and talazoparib **15**.

The cytochrome P450 family (CYP19) enzyme aromatase has a key role in the biosynthesis of the aromatic C18 estrogens estradiol and estrone from the C19 androgens androstenedione and testosterone. High levels of estrogens are associated with stimulation of hormone-dependent BC (HDBC) and metastasis in both pre- and post-menopausal women [11]. The inhibition of aromatase is an important clinically validated approach in the clinical management of hormone-dependent BC, particularly in post-menopausal patients [12]. Clinically approved aromatase inhibitors (AIs) include the steroid exemestane **5**, which binds covalently with the heme iron in the catalytic site, and the triazole containing AIs, such as letrozole **6** and anastrozole **7** (Figure 1), which interact reversibly with the aromatase active site [13]. Side effects of AIs include bone loss and cardiovascular disease, and resistance is emerging [14,15]. Anastrozole was recently authorized for the preventative treatment of post-menopausal women at high risk of BC [16]. Targeted therapies are available for HER2-positive hormone receptor-positive BRCA gene mutations and triple-negative BCs (TNBC) [17]. Targeted drug therapy for HER2-positive BCs includes monoclonal antibodies (trastuzumab, pertuzumab, and margetuximab), antibody–drug conjugate (ADC) ado-trastuzumab emtansine (Kadcyla), and Fam-trastuzumab deruxtecan (Enhertu) together with the kinase inhibitors lapatinib, neratinib, and tucatinib [17]. Targeted therapy for hormone receptor-positive BC includes the CDK4/6 inhibitors palbociclib **8**, ribociclib **9**, and abemaciclib **10**, which are effective with an AI or fulvestrant. The mTOR inhibitor everolimus **11**, the PI3K inhibitor alpelisib **12** [18], and the recently approved AKT inhibitor capivasertib **13** are used in combination with fulvestrant (Figure 2) [19]. Sacituzumab govitecan is a conjugate of the humanized anti-Trop-2 monoclonal antibody linked with the active metabolite of the topoisomerase inhibitor irinotecan and is used in TNBC [20,21]. Targeted therapy for women with *BRCA* gene mutations includes the poly (ADP-ribose) polymerase (PARP) inhibitors olaparib **14** and talazoparib **15**, which can prevent the repair of damaged DNA [22] (Figure 2).

TNBC is characterized by the absence of the ER, progesterone, and HER2 receptors and accounts for 10−15% of breast cancers diagnosed. TNBC is associated with an increased risk of recurrence and an unfavorable prognosis. Cytotoxic chemotherapy in combination with PARP inhibition has demonstrated efficacy in BRCA1/2-mutated TNBC patients, while immune therapies have emerged as promising targeted therapies specifically for TNBC patients [23,24,25,26]; however, new approaches to novel targeted therapeutic strategies are still urgently required [27]. Allosteric Hsp90 C-terminal domain (CTD) inhibitors were recently reported as potential TNBC therapeutics [28].

**Figure 3:**
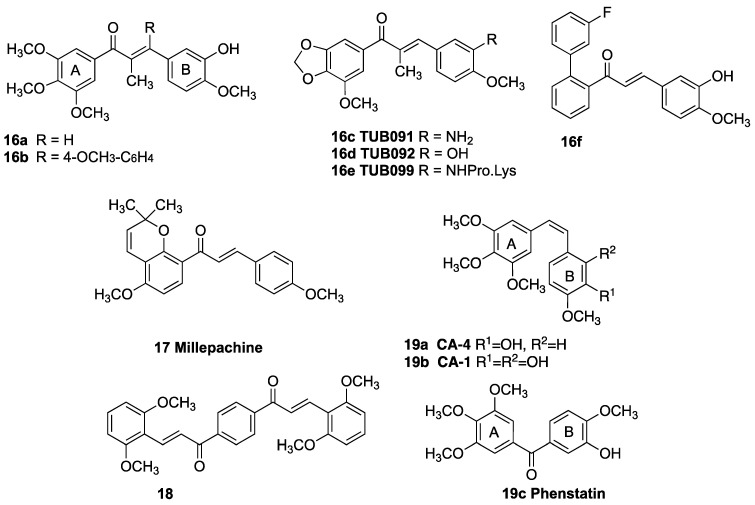
Antiproliferative chalcones and related compounds that target the colchicine binding site of tubulin: α-methylchalcones **16a–e**, O-arylchalcone **16f**, millepachine **17**, bischalcone **18**, combretastatins CA-4 **19a** and CA-1 **19b**, and phenstatin **19c**.

Chalcones, containing an α,β-unsaturated ketone fragment, are important pharmacologically active agents with diverse biological activities [29,30,31,32]. Due to their abundance in plants and ease of synthesis, the chalcone class of compounds has continued to attract interest in potential therapeutic uses such as antidiabetic, anti-inflammatory, antiparasitic, antimicrobial, and antifungal agents [33,34,35] and neurodegenerative conditions [36]. Chalcones substituted with a triazole at the α-position of the ketone demonstrated antibacterial activity [37]. Chalcone-based structures have demonstrated promise as agents for the treatment of human cancers [38,39] as they promote apoptosis [40] and inhibit tubulin assembly by interacting with the colchicine-binding site of tubulin [41,42]. α-Methylchalcone **16a** is a potent anticancer and antimitotic agent with IC_50_ = 0.21 nM in the K562 human chronic myelogenous leukemia cell line [43] (Figure 3). The α-arylchalcone **16b**, designed as a mimic of podophyllotoxin, demonstrated potent antiproliferative activity with inhibition of tubulin assembly [44]. The α-methylchalcones TUB091 **16c**, TUB092 **16d**, and water soluble prodrug TUB099 **16e** showed potent antitumor activity in melanoma and BC xenograft models, while X-ray studies confirmed the interaction of TUB092 at the colchicine binding site of tubulin [45]. Additional examples include the O-arylchalcone **16f** active against multidrug-resistant cancers [46], the antimitotic millepachine **17** [47], and the bis-chalcone **18** identified as a BC resistance protein ABCG2 inhibitor [48].

There is considerable interest in the development of multitarget-directed ligands, which may have the potential to improve clinical outcomes and resistance [49]. Dual targeting BC agents include ER/tubulin [50], tubulin/HSP90 [51], and ER/AI, e.g., norendoxifen and endoxifen [52,53,54] and related compounds [55,56], sulfatase/AI [57], tubulin/sulfatase [58], ER/histone deacetylase [59], and ER*α*/aromatase PROTAC degraders [60].

**Figure 4:**
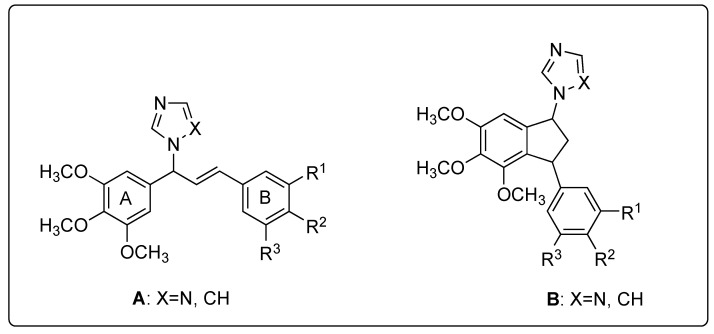
Target structures **A** (chalcone-based) and **B** (indane-based) for synthesis.

The rationale in designing the target compounds: The series of (*E*)-1-(3-(4-methoxyphenyl)-1-(3,4,5-trimethoxyphenyl)allyl)-1*H*-1,2,4-triazoles and related compounds are designed as hybrid scaffolds derived from the tubulin targeting combretastatins CA-1 **19a** and CA-4 **19b** [61], phenstatin **19c** [62], and the chalcone **16a**, together with the 1,2,4-triazole characteristic of the aromatase inhibitor letrozole **2** [63]. These compounds are designed to target tubulin polymerization and could also be effective by inhibiting estrogen production [64]. We previously reported 1-(diarylmethyl)-1*H*-1,2,4-triazoles and 1-(diarylmethyl)-1*H*-imidazoles derivatives as tubulin inhibitors, which demonstrated aromatase inhibitory activity [65], while phenstatin/isocombretastatin–chalcone conjugates are reported as potent tubulin polymerization inhibitors [66]. The target hybrid structures (chalcone-based scaffold **A**) are shown in Figure 4. In addition, a number of related hybrid compounds containing the indane-based scaffold structure **B** were also investigated. The objective of this strategy was the development of novel tubulin inhibitors in BC cells with potential dual-targeting aromatase inhibition.

## 2. Chemistry

**Scheme 1:**
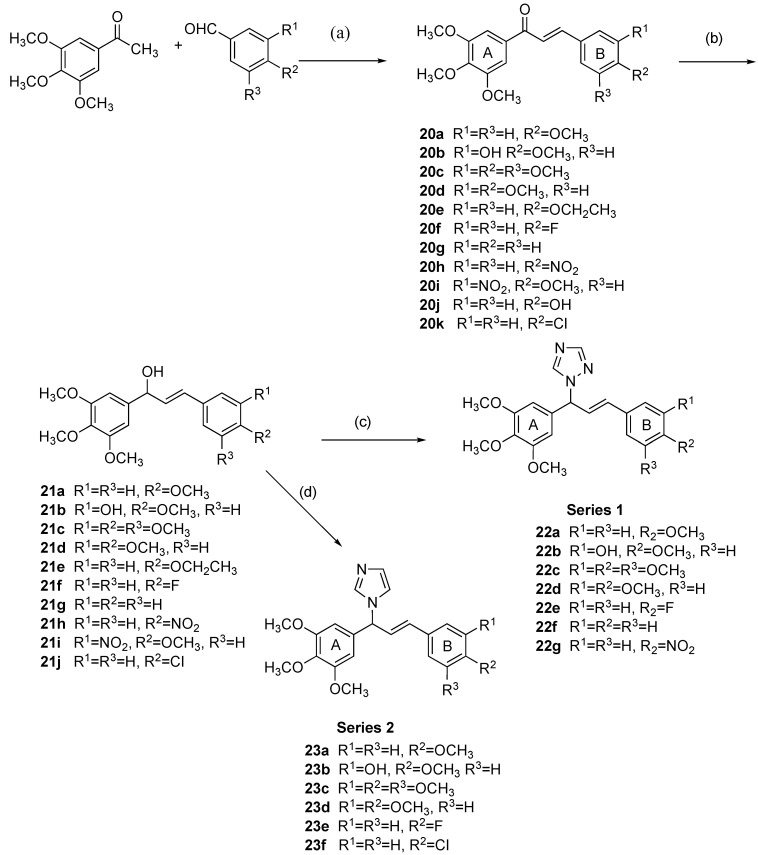
Synthesis of (*E*)-1-(3-aryl)-1-(3,4,5-trimethoxyphenyl)allyl)-1*H*-1,2,4-triazoles **22a–g** (Series 1) and (*E*)-1-(3-(aryl)-1-(3,4,5-trimethoxyphenyl)allyl)-1*H*-imidazoles **23a–e** (Series 2): reagents and conditions (**a**): KOH, methanol, 20 °C (27–87%) (**b**): NaBH_4_, MeOH/THF, 1 h, 20 °C (85–100%); (**c**) *p*-TSA, 1,2,4-triazole, toluene, microwave, 4 h (30–76%); (**d**) CDI, dry ACN, reflux, 1 h (26–45%).

The synthesis of a panel of chalcones containing the 3,4,5-trimethoxyphenyl group (A ring) followed by the introduction of the heterocyclic 1,2,4-triazole or imidazole onto C-1 of the α,β-unsaturated ketone system is illustrated in Scheme 1 (Steps (c) and (d)). The imidazole and triazole heterocycles are introduced into the chalcone scaffold structure to explore the effect on antiproliferative and tubulin activity. The 3,4,5-trimethoxyaryl group (ring A) is retained as it is considered to be required for optimal interaction with the colchicine binding site of tubulin [67], while the substituents on the B ring are varied. Reduction in the chalcone carbonyl group to afford the alcohol and subsequent chlorination and substitution with either the 1,2-4-triazole or imidazole were explored to afford the target compounds as illustrated in Scheme 1.

The panel of chalcones **20a–k** was prepared by Claisen–Schmidt condensation reactions of 3,4,5-trimethoxyacetophenone with the appropriate aryl aldehyde using the base potassium hydroxide in yields of 27–87% (Scheme 1). The A ring 3,4,5-trimethoxyaryl substituent of the synthesized chalcones was chosen to mimic the A ring present in phenstatin **19c** and CA-4 **19a** was regarded as required for antiproliferative activity in prostate and colon cancer cells [68]. The B ring contains a number of diverse substituents (OCH_3_, OCH_2_CH_3_, OH, F, NO_2_), together with the 3-hydroxy-4-methoxyphenyl Ring B characteristic of **19a** and **19c**. Substituents on the B ring are at C-4 (compounds **20a**, **20e**, **20f**, **20h, 20j**, and **20k**), C-3 and C-4 (compounds **20b**, **20d**, and **20i**), or C-3, 4, and 5 as in compound **20c** where both rings A and B contain the 3,4,5-trimethoxy substitution pattern. Compound **20b** is structurally related to **19a** and **19c** since it not only carries the 3,4,5-trimethoxy on the A ring but also the 3-hydroxy-4-methoxy substituents on the B ring [39]. In this work, **19c** was prepared as a control in the biochemical screen [62,65,69].

In the ^1^H-NMR spectrum of the chalcone **20a**, the signals of the alkene protons are identified as doublets at δ 7.34 and δ 7.78, *J* = 15.3 Hz *(trans)* [44], and confirm the thermodynamically more stable *E* isomer obtained [29]. In the ^13^C-NMR spectrum of the chalcone **20a**, the signal at 189.3 ppm is assigned to the carbonyl while the C-2 (α) and C-3 (β) are observed at 119.4 ppm and 144.6 ppm, respectively. Reduction in the chalcones **20a–j** with sodium borohydride afforded secondary alcohols **21a–i** in good yields (85–100%), Scheme 1, Step (b). In the ^1^H-NMR spectrum, the *E* structure was retained following the reduction reaction with *J* values for the alkene protons observed in the range 15–17 Hz. From the ^1^H-NMR spectrum of compound **21a,** the alkene proton H-2 (α-H) is observed as a double doublet at δ 6.23 (*J* = 15.8 Hz and 7.1 Hz) and alkene H-3 is assigned as the doublet δ 6.59 (*J* = 16.6 Hz). The doublet δ 5.30 (*J* = 9.5 Hz) is assigned to the tertiary C-1 proton (CH-OH). In the ^13^C-NMR spectrum of **21a**, the tertiary carbon C-3 is observed at 75.4 ppm, and the alkene C-2 (α) and C-3 (β) occur at 127.9 ppm and 129.1 ppm, respectively.

The panel of novel hybrid compounds **22a–g** containing the heterocycle 1,2,4-triazole and 3,4,5-trimethoxy moiety was prepared by reacting the secondary alcohols **21a–d** and **21f–h** with *p*-TSA and 1,2,4-triazole and were obtained in yields of 30–76%, (Scheme 1, Series 1, Step (c)). In the ^1^H-NMR spectra of compounds **22a–g**, the characteristic signals for the triazole ring protons are observed in the region δ 8.02–8.18 (H-3, H5); the doublet at δ 6.05–6.19 (*J* = 6.8 Hz) is assigned to the tertiary CH at C-1, while the two alkene protons partially overlap with the aromatic proton signals in the region δ 6.11–6.85. Some multiplicity is observed for NMR signals of triazole compounds **22a–g**, possibly due to triazole tautomerization [70,71]. In the ^13^C spectrum, the tertiary carbon (C-1) is identified in the region 63.9–66.3; the signal at 142.5–142.87 ppm is assigned to C-5 of the 1*H*-1,2,4-triazole, while the triazole C-3 is observed in the region 151.9–152.3 ppm.

In a further extension of the hybrid compound design, a related panel of imidazole chalcone derivatives **23a–f** was prepared from the secondary alcohols **21a–d**, **21f**, **21j** by treatment with 1,1′-carbonyldiimidazole (CDI) in acetonitrile in 26–45% yield (Series 2, Scheme 1, Step (d)). In the ^1^H-NMR spectrum of compound **23c**, the doublet at δ 5.87 (*J* = 6.2 Hz) is assigned to the tertiary CH. The protons of the imidazole ring can be observed as three broad singlets: δ 6.98, δ 7.16, and δ 7.67 assigned to H-5, H-4, and H-2 of the imidazole ring. The signals for the alkene protons overlap with the aromatic signals and can be identified as a multiplet at δ 6.44 for the β-proton and a multiplet at δ 6.35 for the α-proton. In the ^13^C-NMR spectrum of compound **23c**, the signals at 63.4, 125.8, and 131.1 ppm are assigned to the tertiary CH, C-2, and C-3, respectively.

**Scheme 2:**
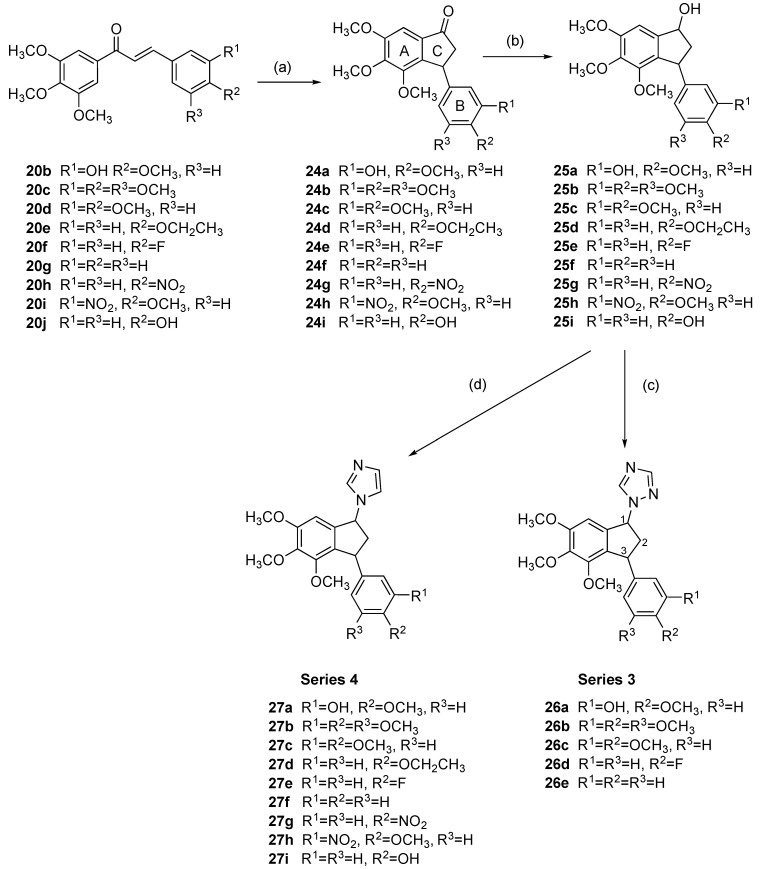
Synthesis of 1-(3-aryl-4,5,6-trimethoxy-2,3-dihydro-1*H*-inden-1-yl)-1*H*-1,2,4-triazoles **26a–e** (Series 3) and 1-(3-aryl-4,5,6-trimethoxy-2,3-dihydro-1*H*-inden-1-yl)-1*H*-imidazoles **27a–i** (Series 4). Scheme reagents and conditions: (**a**) TFA, 120 °C, 10 min microwave (44–96%); (**b**) NaBH_4_, MeOH/THF (1:1), 0–20 °C (43–100%); (**c**) *p*-TSA, 1,2,4-triazole, toluene, microwave, 4 h (30–54%); (**d**) CDI, dry acetonitrile, reflux, 3 h (4–70%).

Indanone-containing compounds are well known in medicinal chemistry in several pharmaceutical areas with many diverse applications [72], e.g., in neurodegenerative conditions [73,74], and with anti-infective [75,76], anti-inflammatory [77], and antioxidant activities [75]. The anticancer activity of cyclic chalcone analogs (benzylidene indanones) has been reported [78,79,80,81,82,83] together with applications such as indanone-based fluorogenic probes and biosensors [84]. Structurally, 3-phenylindanones represent a class of chalcone analog in which the β-carbon of the corresponding chalcone is bonded directly to the C-2 of the A-ring and can be synthesized by cyclization of the corresponding chalcone. In the present work, the synthesis of a series of hybrid compounds derived from 3-phenylindanones and the heterocycles imidazole and 1,2,4-triazole were next investigated (Scheme 2). 3-Phenylindanones can be prepared by treating chalcones in a sealed tube with trifluoroacetic acid for 4 to 24 h in a Nazarov cyclization reaction [80]. In the present work, the synthesis of the indanone derivatives **24a–i** from the chalcones **20b–j** was efficiently achieved via microwave reaction in yields of 44–96%, Scheme 2, Step (a). The reaction time was reduced from 4 h to 10 min with improved yields, e.g., 76% compared with 42% for **24b** [85]. Only chalcones with electron-donating groups on the aromatic ring of the benzoyl moiety (such as the 3,4,5-trimethoxy substituent in ring A) undergo Nazarov cyclization to their respective indanone possibly due to deactivation of the carbonyl group [80]. The substituents present on the B ring are small electron-donating or electron-withdrawing groups. From the ^1^H-NMR spectrum of compound **24b**, the double doublets at δ 2.63 (*J* = 19.1 Hz and *J* = 2.5 Hz) and δ 3.19 (*J* = 19.3 Hz and *J* = 8.1 Hz) are assigned to the C-2 methylene protons. The double doublet at δ 4.52 (*J* = 8.3 Hz and *J* = 2.5 Hz can be assigned to H-3 of ring C. From the ^13^C-NMR spectrum of compound **24b**, the signal at 42.0 ppm was assigned to the C-3 of Ring C ring, the signal at 47.0 ppm was assigned to the methylene C-2 of the 5-membered ring, and the carbonyl signal was identified at 205.8 ppm.

The indanones **24a–i** were next reduced with sodium borohydride to afford alcohols **25a–i** in good yields (43–100%) (Scheme 2, Step (b)). The alcohols were obtained as diastereomeric mixtures, due to the presence of the stereogenic centers at C-1 and C-3. The indanol scaffold was confirmed from the IR spectrum with a broad hydroxyl band in the region 3400–3600 cm^−1^. In the ^1^H-NMR spectrum of **25c**, the double doublet at δ 5.17 (*J* = 7 Hz and 5 Hz) was assigned to the C-1 proton. The double doublet at δ 4.28 (*J* = 8.3 Hz and 5.8 Hz) was assigned to the C ring H-3. The multiplet δ 2.97 (*J* = 13.7 Hz, 8.3 Hz, and 7.5 Hz, ddd) and the multiplet centered at δ 1.95 are assigned to the methylene protons of ring C. From the ^13^C-NMR spectrum, the methine H-1 was observed at 75.75 ppm. The 3-aryl-1-indols **25a–c**, **e**, **f** were then reacted with 1,2,4-triazole as before in a microwave-assisted reaction using *p*-TSA as a catalyst to afford the triazole derivatives **26a–e** in 30–54% yield, (Series 3, Scheme 2, Step (c)). These compounds were obtained as diastereomeric mixtures due to the presence of the two stereogenic centers (C1 and C3), and there is evidence of multiple signals in the ^1^H-NMR spectra. In the ^1^H-NMR spectrum of compound **26d**, the multiplets centered at δ 2.41 and δ 2.92 were assigned to the methylene protons of ring C. The double doublet at δ 4.46 was assigned to H-3; the multiplet signal at δ 5.87 was assigned to the C1 methine proton adjacent to the triazole ring while the singlets at δ 8.01 and δ 8.15 were characteristic of the triazole protons. In the ^13^C-NMR spectrum of compound **26d** the quaternary aromatic C-F was observed as a doublet at 160.3 ppm (*J* = 244 Hz); the signals at 43.5 ppm, 46.3 ppm, and 64.5 ppm were assigned to the methylene carbons C-2, C-3, and C-1, respectively. The triazole carbon signals appear at 143.1 ppm (C-5) and 152.3 (C-3) ppm.

As a further extension of this work, the reduced indanones **25a–i** were reacted with CDI to afford a series of imidazole-containing products **27a–i** in yields of up to 70% (Series 4, Scheme 2, step (d)). All compounds were obtained as diasteromeric mixtures due to the presence of two stereogenic centers (C1 and C3). In the ^1^H-NMR spectrum of compound **27c**, the multiplets at δ 2.17 and δ 3.22 were assigned to the C-2 methylene protons of ring C. The double doublet δ 4.64 (*J* = 7.7 and 3.9 Hz) was assigned to H-3, while the triplet at δ 5.77 (*J* =7.5 Hz) was assigned to the methine proton H-1. The imidazole ring protons were identified at δ 7.56 (H-2), δ 6.79 (H-4), and δ 7.10 (H-5). In the ^13^C-NMR spectrum of **27c**, the ring C carbons are identified at 45.8 ppm (CH_2_), 46.8 ppm (C-3), and 61.6 ppm (C-1). The imidazole ring carbons were identified at 137.6 ppm (C2), 129.8 ppm (C4), and 118.7 ppm (C5). The novel imidazole and triazole hybrids synthesized retain the main structural features of the antimitotic CA-4, phenstatin, and chalcones such as **20b**, together with the azole of letrozole (Figure 1). The synthesis of these compounds allowed the investigation of the potential biological activity change when the azole ring is introduced into the structure.

**Scheme 3:**
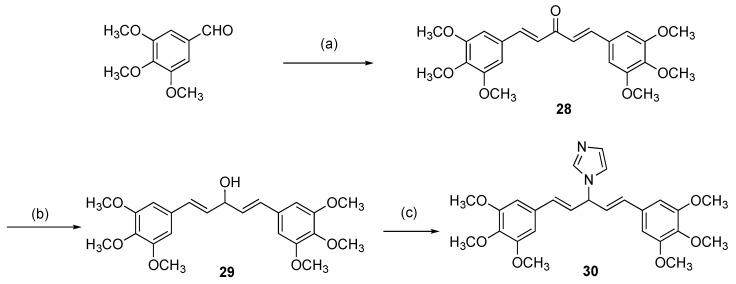
Synthesis of 1-((1*E*,4*E*)-1,5-bis(3,4,5-trimethoxyphenyl)penta-1,4-dien-3-yl)-1*H*-imidazole **30**. Reagents and conditions: (**a**): Acetone, EtOH, NaOH (10%, aqueous), 30 min, 20 °C (68%); (**b**): NaBH_4_, MeOH/THF, 1 h, 20 °C (92%); (**c**) CDI, dry ACN, 3 h, reflux (27%).

**Scheme 4:**
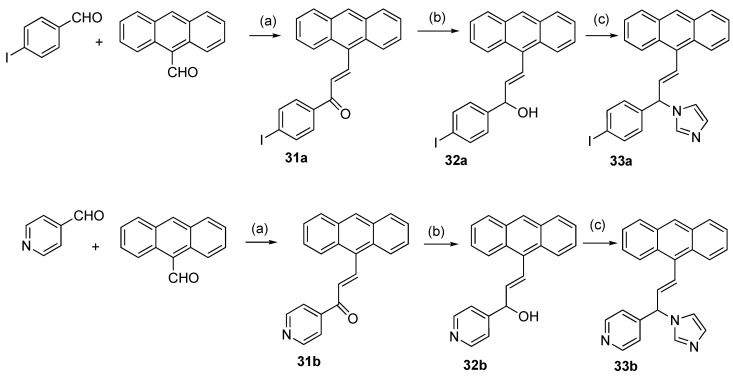
Synthesis of (*E*)-3-(anthracen-9-yl)-1-(4-iodophenyl)allyl)-1*H*-imidazole (**33a**) and (*E*)-3-(anthracen-9-yl)-1-(4-pyridyl))allyl)-1*H*-imidazole (**33b**): reagents and conditions: (**a**): KOH, methanol, 20 °C (49–82%) (**b**): NaBH_4_, MeOH/THF, 1 h, 20 °C (78–98%); (**c**) CDI, dry ACN, reflux, 1 h (5–58%).

Additional structural variation was investigated by the reaction of acetone with 3,4,5-trimethoxybenzaldehyde, which afforded the ketone product **28** (68%), which was reduced with sodium borohydride to afford the alcohol **29** (92%). Subsequent reaction with CDI gave the imidazole product **30** (27%), Scheme 3. In a further extension of this work, the anthracene-based chalcones **31a** and **32b** were prepared by condensation of the anthracene carbaldehyde with the appropriate aryl ketones; reduction of these α,β-unsaturated ketones with sodium borohydride afforded the alcohols **32a** and **31b**, which were treated with CDI to give the imidazole products **33a** and **33b**, respectively (Scheme 4).

## 3. Biochemical Studies

**Figure 5:**
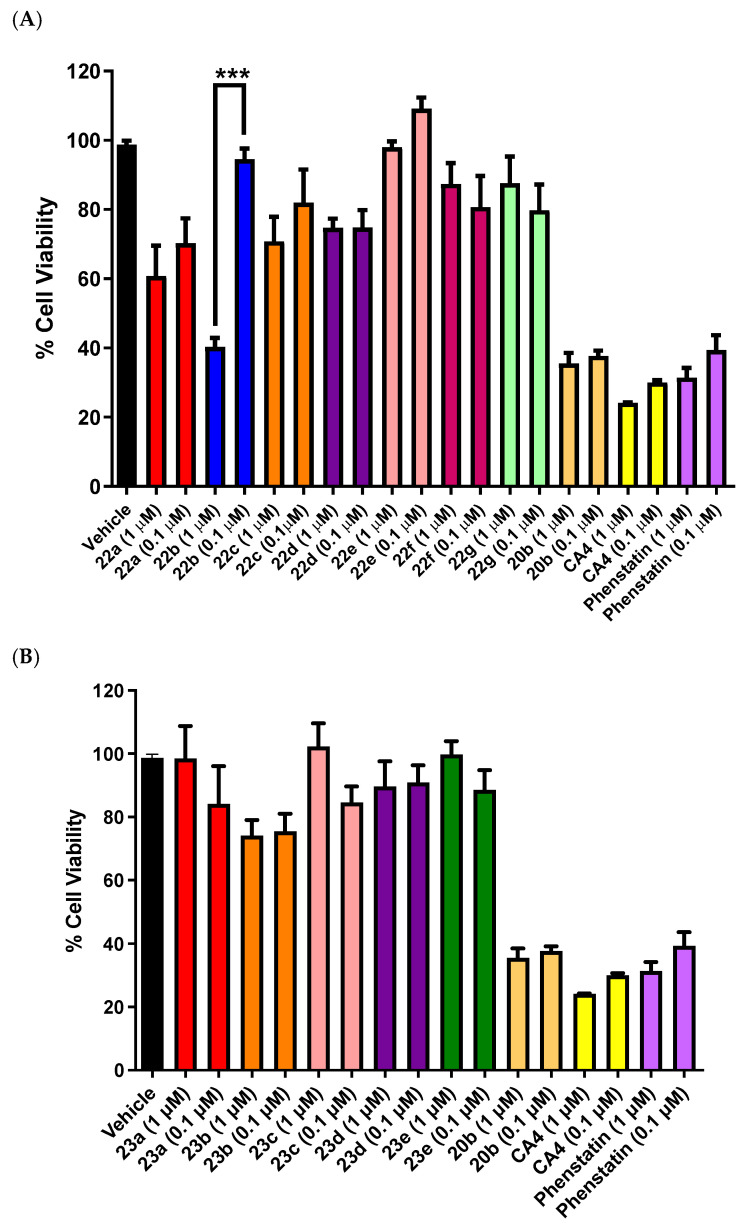
Preliminary cell viability data for Series 1: (**A**) compounds **22a–22g** and chalcone **20b** and Series 2: (**B**) compounds **23a–e** and chalcone **20b** in MCF-7 breast cancer cells. Cell proliferation of MCF-7 cells was determined with an alamarBlue assay (seeding density 2.5 × 10^4^ cells/mL per well for 96-well plates). Compound concentrations of either 1 or 0.1 μM for 72 h were used to treat the cells (in triplicate) with control wells containing vehicle ethanol (1% *v*/*v*). The mean value ± SEM for three independent experiments is shown. The positive controls used are CA-4 and phenstatin (1.0 μM and 0.1 μM). Statistical analysis was performed using One-way ANOVA with the Sidak multiple comparison test (***, *p* < 0.001).

**Figure 6:**
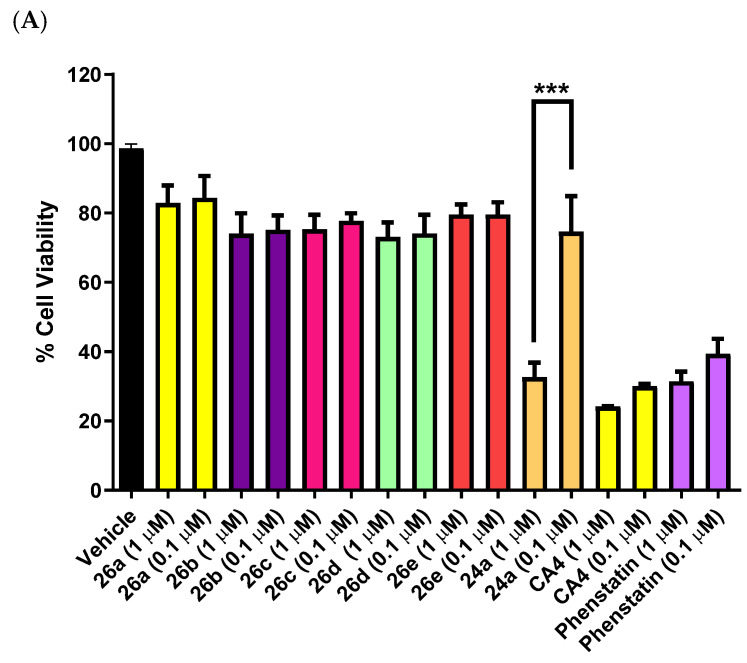

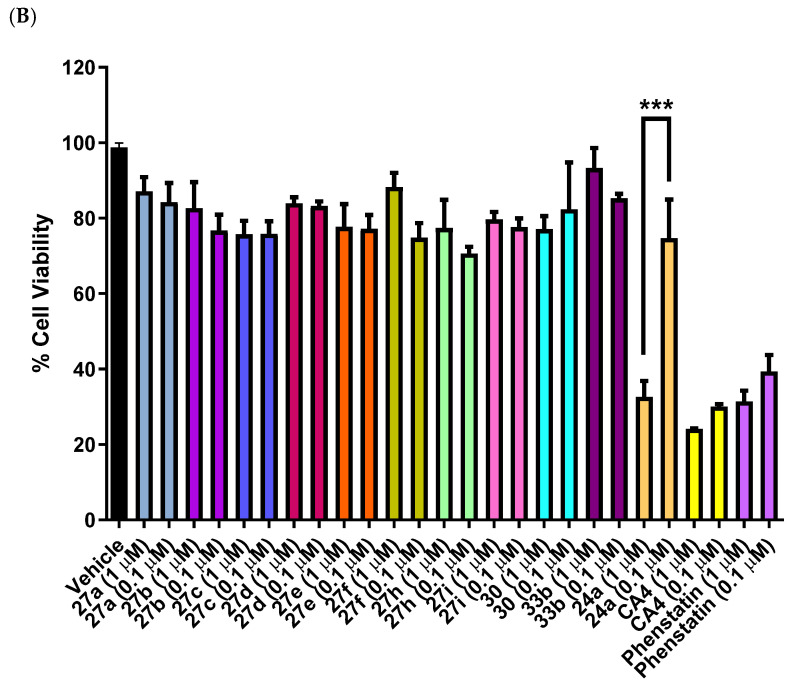
Preliminary cell viability data for (**A**) triazoles **26a–e** and related indanone **24a** and (**B**) imidazoles **27a–f**, **27h**, **27i** and related compounds **30** and **33b** in MCF-7 breast cancer cells. Cell proliferation of MCF-7 cells was determined with an alamarBlue assay (seeding density 2.5 × 10^4^ cells/mL per well for 96-well plates). Compound concentrations of either 1 or 0.1 μM for 72 h were used to treat the cells (in triplicate) with control wells containing vehicle ethanol (1% *v*/*v*). The mean value ± SEM for three independent experiments is shown. The positive controls used were CA-4 and phenstatin (1.0 μM and 0.1 μM). Statistical test was performed using One-way ANOVA with Sidak multiple comparison test (***, *p* < 0.001).

**Figure 7:**
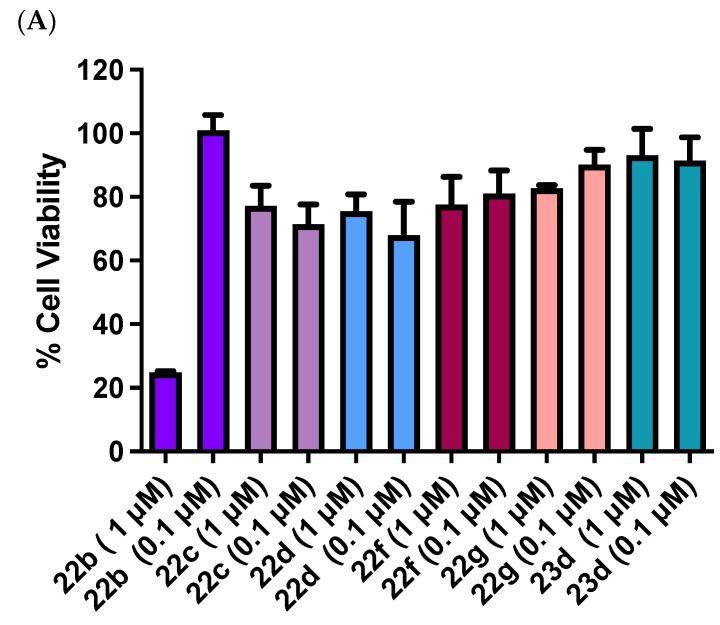

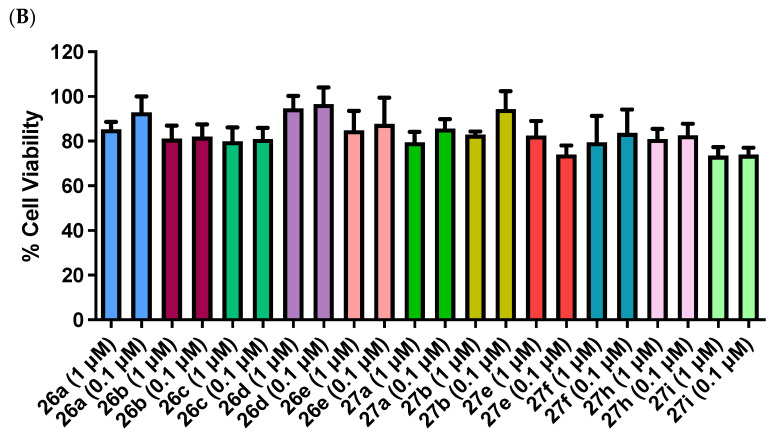
Preliminary cell viability data for (**A**) triazoles **22b–d**, **22f**, **22g** and imidazole **23d** and (**B**) triazoles **26a–e** and imidazoles **27a**, **27b**, **27e**, **27f**, **27h** and **27i** in HL-60 cells. Cell proliferation of HL-60 cells was determined with an alamarBlue assay (seeding density 2.5 × 10^4^ cells/mL per well for 96-well plates). Compound concentrations of either 1 or 0.1 μM for 72 h were used to treat the cells (in triplicate) with control wells containing vehicle ethanol (1% *v*/*v*). The mean value ± SEM for three independent experiments is shown. The positive control was CA-4 (1.0 μM and 0.1 μM).

The panel of compounds synthesized was initially evaluated for cytotoxic effects on human estrogen and progesterone receptor positive BC cell line MCF-7, triple-negative MDA-MB-231, and the promyelocytic leukemia cell line HL-60. An initial screening of the compounds using the alamarBlue cell viability assay was used to identify the most potent compounds and to establish structure–activity relationships for the series of compounds. The related benzophenone phenstatin **19c [62]** was prepared for use as a positive control (IC_50_ value 34 nM in MCF-7 cells [86]), together with the stilbene combretastatin CA-4 (**19a**) (IC_50_ = 4 nM) [87] as previously reported. The synthetic intermediates chalcone **20b** and indanone **24a** were also screened, to enable further structure–activity relationships to be determined. The results obtained from this preliminary screen evaluation at compound concentrations of 1 μM and 0.1 μM are displayed in Figure 5, Figure 6 and Figure 7. Those compounds showing potential activity (cell viability < 60% at 1 μM) were selected for further evaluation in MCF-7 and in additional cell lines. The positive controls used were CA-4 **19a** (24% viable cells at 1 μM) and phenstatin **19c** (30% viable cells at 1 μM), which demonstrated a potent antiproliferative effect in these experiments. Ethanol (1% *v*/*v*) was the vehicle control (with 99% cell viability). The antiproliferative results obtained for these novel compounds are discussed by structural type (Series 1–4).

### 3.1. Preliminary Screening of SERIES 1 Chalcone 1,2,4-Triazole Derivatives in MCF-7 Cells

The panel of chalcone triazole derivatives **22a–g** (Series 1) was evaluated in MCF-7 cells at concentrations of 1 μM and 0.1 μM (Figure 5A). The substituents on the aryl rings were the 3,4,5-trimethoxyphenyl on the A ring of each compound, and the substituents on the B ring were various methoxy and 3-hydroxy-4-methoxy groups and fluorine in compound **22e**. Compound **22b** displayed the most promising activity with 40% viable cells at 1 μM. It is of interest that **22b** contains the 3-hydroxy-4-methoxy substituents on the B ring, which are also present in phenstatin, CA-4, and other related tubulin-targeting compounds. This result also indicated that the introduction of the additional alkene in the structure of **22b** resulted in retention of antiproliferative activity in MCF-7 cells, although it was less active than the corresponding triazole phenstatin-based derivatives [65]. The next most active compound of the series was compound **22a** (containing the *p*-methoxy ring B), which demonstrated 60% cell viability at 1 μM. Interestingly, compound **22c** (3.4.5-trimethoxysubstituted Ring B) and **22d** (3,4-dimethoxy phenyl Ring B) demonstrated 70% and 74% cell viability, respectively, at 1 μM. The IC_50_ value of compound **22b** in MCF-7 cells was determined as 0.385 ± 0.12 μM. The synthetic precursor chalcone compound **20b** [39,85,88] was used as a positive control in the viability assay (IC_50_ 0.067 ± 0.017 μM). The introduction of the triazole ring on the chalcone structure reduced the antiproliferative activity five-fold. However, the triazole ring is necessary for desired aromatase activity so was retained in subsequent compounds. The hybridization of chalcones with other pharmacophores through the 1,2,3-triazole ring has afforded products with interesting pharmacological activities [89,90,91].

### 3.2. Preliminary Screening of Chalcone Imidazole Derivatives in MCF-7 Cells (Series 2)

The panel of chalcone imidazole derivatives **23a–e** was next evaluated at concentrations of 1 μM and 0.1 μM (Series 2, Figure 5B) in MCF-7 cells. The substituents on the aryl rings were the 3,4,5-trimethoxyphenyl on the A ring of each compound, and the substituents on the B ring were various methoxy and 3-hydroxy-4-methoxy groups (**23a–d**) and a fluoro component in compound **23e**. None of the compounds tested were particularly active; the most potent compound of the series was compound **23b** (74% viable cells at 1 μM and 75% viable cells at 0.1 μM), but the activity was not comparable to the previous related chalcone triazole compound **22b** (40% viable cells at 1 μM, IC_50_ = 0.385 ± 0.12 μM) and was not selected for further analysis. These results identified the triazole compound **22b** as the most potent compound in the Series 1 and Series 2 panels and demonstrated the selective effect of interchanging the imidazole and 1,2,4-triazole rings on cell viability in MCF-7 cells.

### 3.3. Preliminary Screening of Triazole and Imidazole Derivatives of Indanones in MCF-7 Cells (Series 3 and Series 4)

The triazole and imidazole derivatives of the 3-phenylindanones **26a–e** and **27a–i** were evaluated at 1 and 0.1 μM concentrations in MCF-7 cells (Series 3 and Series 4, Figure 6A,B). When compared with the activity of the chalcone compounds (Series 1 and 2), the indanone series 3 and 4 compounds were not as effective. Cell viability was greater than 70% for **26a–e** and **27a–i** and they were not selected for further studies. The 1,2,4-triazole-indane derivatives **26a–e** (Series 3) and imidazole-indane derivatives **27a–i** (Series 4) evaluated did not show significant activity at 1 and 0.1 μM concentrations tested in MCF-7 cells, with cell viability > 70%, and were not selected for further studies. The indanone compound **24a** (a synthetic precursor of compounds **26a** and **27a)** [80,81,85] was used as a positive control for the indane Series 3 and Series 4 with cell viability of 35% and 70% at 1 μM and 0.1 μM concentrations, respectively, in MCF-7 cells. This result indicates that the carbonyl group of indanone **24a** is essential for antiproliferative activity and replacement by the azole (triazole or imidazole) reduces the potency of the compound in the MCF-7 cell line. The 1,5-bis((3,4,5-trimethoxyphenyl)penta-1,4-dien-3-yl)-1*H*-imidazole **30** and also the imidazole anthracene chalcone **33b** were evaluated in MCF-7 cells, but both were found to have poor potency (Figure 6B), with cell viability of 77% and 92%, respectively, at 1 μM. This result confirms that the chalcone core scaffold is required for the significant antiproliferative effect in these compounds; replacement by the anthracene-chalcone (as in **33b**) or the bis-chalcone (as in **30**) does not provide an effective pharmacophore for interaction with the tubulin binding site.

### 3.4. Preliminary Screening of Azole-Containing Chalcone and Indane Hybrids in Leukemia HL-60 and Triple-Negative MDA-MB-231 Breast Cancer Cells

A series of azole-containing chalcone hybrids (**22b–d, f, g** and **23d**) were evaluated on the promyelocytic leukemia HL-60 cell line at two concentrations: 1 μM and 0.1 μM (Figure 7A,B). Although these compounds showed moderate activity in MCF-7 cells, it was decided to screen some of them in leukemia HL-60 cells due to the known activity of polymethylated chalcones on leukemia cells previously reported by Ducki et al. [92]. Chalcones **22c**, **d**, **f**, **g** and **23d**, both with the 1,2,4-triazole and with the imidazole, failed to show significant activity, with a percentage of viable cells between 75 and 95%, and were not selected for further studies. However, the IC_50_ value of compound **22b** was determined as 0.366 ± 0.13 μM in the HL-60 cell line at 72 h, which is slightly less potent than the corresponding phenstatin triazole derivative that we previously reported (0.261 μM) [65]. The screening of the indane derivatives **26a-e**, **27a**, **b**, **e**, **f**, **h**, **i** (both with 1,2,4-triazole and imidazole) in the leukemia HL-60 cell line at 1 μM and 0.1 μM is displayed in Figure 7B. Of all the compounds tested, the triazoles **26f** and **26i** were slightly more active (73% viable cells at 1 and 0.1 μM) compared to the remaining compounds of the series. However, this was not deemed sufficient to proceed with further evaluation.

From the chalcone library, compound **22b** was selected for evaluation in triple-negative MDA-MB-231 breast cancer cell lines and gave an IC_50_ value of 0.765 ± 0.030 μM. Compound **22b** demonstrated superior activity when compared to the corresponding phenstatin-triazole derivative, which we previously reported with an IC_50_ value of 0.978 ± 0.130 μM [65]. The IC_50_ values determined for CA-4 control in this assay are in agreement with the reported IC_50_ values for CA-4 in MCF-7 (0.0039 ± 0.00032 μM), MDA-MB-231 (0.0430 μM), and HL-60 (0.0019 ± 0.0005 μM) [87,93,94].

In summary, the preliminary screening results above identified the triazole compound **22b** as the most potent synthesized compound in the Series 1 and Series 2 panels of chalcone-azole hybrid compounds. This result demonstrated the selective effect of interchanging the imidazole and 1,2,4-triazole rings positioned at C-1 of the chalcone structure on cell viability in MCF-7 cells, with the triazole ring displaying superior potency when directly compared with the imidazole series. The IC_50_ value of compound **22b** in MCF-7 cells was determined as 0.385 ± 0.12 μM. Compound **22a** (containing the *p*-methoxy ring B) was less active with 60% cell viability at 1 μM. From the chalcone library, compound **22b** was evaluated in the triple-negative MDA-MB-231 breast cancer cell line (IC_50_ value = 0.765 ± 0.030 μM) and in the HL-60 leukemia cell line (IC_50_ = 0.366 ± 0.13 μM). Although the introduction of the triazole ring on the chalcone scaffold structure **20b** reduced its antiproliferative activity by 5-fold, the inclusion of the 1,2,4-triazole ring in the hybrid structure is necessary for the desired aromatase activity so was retained in subsequent compounds. When compared with the activity of the chalcone-based compounds (Series 1 and 2), the indane-based compounds (Series 3 and 4) were not as effective as antiproliferative agents and were not selected for further development. Although similar pharmacophores are present in both the chalcone **22b** and the indane **26a** (e.g., the 3,4,5,-trimethoxyaryl Ring A and 3-hydroxy-4-methoxyphenyl Ring B), it may be that the flexibility contained in the chalcone-based structure **22b** is better accommodated at the target colchicine binding site of tubulin than the conformationally constrained indane compound **26a**.

### 3.5. NCI 60 Cell Line Panel Screening

The National Cancer Institute (NCI), the Developmental Therapeutics Program (DTP), has utilized a panel of 60 human tumor-derived cell lines to screen the chemotherapeutic potential of novel chemical compounds and provides in vitro biological data for compounds evaluated on nine different types of cancer. The 60 cell lines include nine major groups of human cancer: leukemia, non-small cell lung, colon, CNS (central nervous system), melanoma, ovarian, renal, prostate, and breast cancers. The growth inhibition properties of the compounds were calculated at a single dose (10^−5^ M) first and subsequently using five different concentrations in the range 10^−4^–10^−8^ M. The incubation time was 48 h, and the test performed was the Sulforhodamine B assay. The following results are provided for each compound evaluated by NCI in the 5-dose assay; GI_50_ is the concentration for 50% of maximal inhibition of cell proliferation (similar to the IC_50_ value), TGI signifies a “total growth inhibition” or cytostatic level of effect, and LD_50_ is the concentration causing 50% cell death (LD = lethal dose).

**Figure 8:**
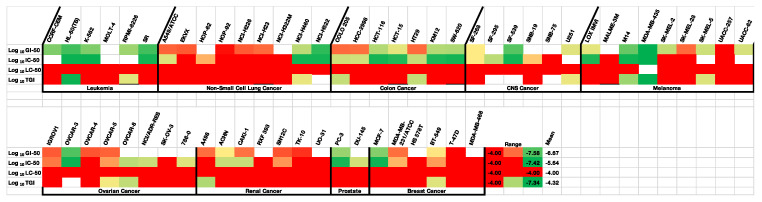
Heatmap for compound **22b** across cell lines in the NCI-60 cell screen. Heatmap for the antiproliferative activity of compound **22b** (NCI 788807), across the cell lines in the NCI-60 screen, using three different values: (growth-inhibitory effect, GI_50_; drug concentration at which the response is reduced by half, IC_50_; cytostatic effect, TGI; cytotoxic effect, LC_50_; concentration in molar). Color key for GI_50_ and IC_50_: green is more sensitive, and red is less sensitive.

Compounds **22a**, **22b**, **23b**, **27a**, and **30** were selected for evaluation by the NCI for the 60-cell line panel for in vitro primary one dose screening (at 10 μM concentration) [95] and the results are displayed in Table 1. It is interesting to see that the mean growth percentages for the compounds at this concentration over the 60-cell line panel were 41.9%, 29.9%, 39.7%, 79.2%, and 81.3% for compounds **22a**, **22b**, **27a**, **23b**, **27a**, and **30**, respectively, confirming that the triazole compound **22b** displays the greatest growth inhibition effects. The mean growth percentage in the BC panel follows a similar trend: 40.1%, 29.1%, 38.1%, 79.1%, and 71.5% for compounds **22a**, **22b**, **23b**, **27a**, and **30,** respectively; while the growth percentage in MCF-7 cells also reflected this trend: 22.5%, 21.2%, 17.9%, 44.8%, and 75.4%, respectively. The mean values in the leukemia panel were also encouraging for the series: 24.0%, 4.2%, 11.2%, 73.2%, and 60.5% for compounds **22a**, **22b**, **23b**, **27a**, and **30**, respectively. The most potent triazole-containing compound **22b** was then selected for the NCI 60 cell line panel screening at five different concentrations (in the range 10^−4^–10^−8^ M) and the results obtained for GI_50_ (concentration for 50% of maximal inhibition of cell proliferation) [95,96] are reported below in Table 2. The results for compound **22b** across the cell lines in the NCI-60 cell screen are also presented as a heatmap using GI_50_, IC_50_, TGI, and LC_50_ values (Figure 8).

Compound **22b** demonstrated potent activity in the sub-micromolar range against leukemia HL-60 cells (GI_50_ = 0.024 μM) and in CNS cancer cells SF-268, SF-539, and U251 with GI_50_ values in the range between 0.026 and 0.059 μM. The activity was also promising in colon cell line SW-620 (GI_50_ = 0.039 μM) and on the two non-small cell lung cancer cell lines NCI-H460 (GI_50_ = 0.042 μM) and NCI-H522 (GI_50_ = 0.022 μM). Of the breast cancer cell lines, the best results were obtained in MCF-7 (GI_50_ = 0.033 μM) and BT-549 (GI_50_ = 0.071 μM). Sub-micromolar GI_50_ values of 0.0183 μM for the MDA-MB-435 melanoma cell line and 0.036 μM for the PC-3 prostate cell line were also obtained. The MID GI_50_ for compound **22b** (the mean of GI_50_ values over all cell lines for the tested compound) was calculated over all 60 cell lines tested and afforded a result of 0.371 μM. The TGI value (total growth inhibition) obtained for **22b** was 57.5 μM over all 60 cell lines while the value obtained for the LC_50_ (concentration at which the number of viable cells is 50% of those present at time zero) was determined as >100 μM over all 60 cell lines, indicating the low toxicity of the compound.

The COMPARE algorithm was used to compare the differential antiproliferative activities of CA-4 hybrids **22b** to compounds with known mechanisms of action in the NCI Standard Agent Database. The COMPARE analysis was performed for compound **22b** and the results obtained are shown in Table S1, Supplementary Information [97]. High correlation values may indicate compounds with a similar mechanism of action, such as anti-tubulin targeting agents. The target set for this analysis was the standard agent database, and the target set endpoints were selected to be equal to the seed end points. Correlation values (*r*) were Pearson correlation coefficients. All three end-points of activity (GI_50_, TGI, and LC_50_) were used. The highest-ranked compound based on TGI values was the tubulin-targeting drug paclitaxel (*r* = 0.703). Based on GI_50_ values, the compounds with high rank were the tubulin-targeting drug vinblastine (*r* = 0.578) and brequinar (*r* = 0.569) [98] and dichloroallyl lawsone (*r* = 0.592) [99], both of which inhibit dihydroorotic acid dehydrogenase (DHO-DH), resulting in a decrease in pyrimidine nucleotide biosynthesis.

### 3.6. Cheminformatics Analysis of Lead Compounds: Physicochemical Properties

The physicochemical characteristics and metabolic properties of selected azole-containing compounds from the series of synthesized compounds were investigated to establish their drug-like features (see Supplementary Information Tables S2–S4). The relevant physicochemical and pharmacokinetic properties of selected compounds **22a–g**, **23a–g**, **27a–i**, **26a–e**, **30**, and **33a–c** were determined using the Swiss ADME cheminformatics webtool [100] (Supplementary Information Figures S1 and S2 and Tables S2–S4). The potential correlations can be identified between the estimated physicochemical properties and biological activity.

The physicochemical properties of the compounds were found to comply with the requirements of Lipinski rules (except compound **33b**), Ghose rules (except compounds **30**, **33a,b**), Veber rules (except compound **30**), Egan rules (except compound **33a**), and Muegge rules (except compounds **33a,b**) with molecular weights in the range 350–486, hydrogen bond acceptor range 1–8, hydrogen bond donor range 0–1, 4–11 rotatable bonds, and logP range 2.62–3.98 for all compounds except **33a,b**. The most potent compound **22b** [IC_50_ = 0.385 μM in MCF-7 cells, IC_50_ = 0.765 μM in MDA-MDA-231 cells and 8.27% growth in MDA-MDA-231 cells] and log P of 2.89 demonstrated a correlation between log P value and antiproliferative activity when compared with compound **22a** with logP of 3.21 and 40.9% growth in MDA-MDA-231 cells. However, the triazole compound **22b** (logP 2.89) was also more potent in MDA-MDA-231 cells (45.5% growth) than the corresponding imidazole **23b** (logP = 3.21, 53.0% growth inhibition), suggesting that the triazole compound **22b** may have a better fit at the colchicine-binding site for these compounds. It is interesting to compare the mean growth percent activities of the compounds over the NCI 60 cell line panel (29.9%, 39.7%, 41.9%, and 81.3% for compounds **22b**, **23b, 22a,** and **30,** respectively), and the correlation with logP values of 2.89, 3.09, 3.21, 2.85, and 3.96 for these compounds, respectively, suggesting that a lower logP value is favorable for growth inhibition; however, the indane-imidazole compound **27a** (logP 2.85) resulted in a mean growth percent of 79.2%, indicating that lipophilicity alone is not a predictor of activity.

The calculated topological polar surface area (TPSA) of this series of compounds was in the range 30.71–100.56 Å^2^, below the required limit of <140 Å^2^ for high gastrointestinal absorption and membrane permeability. In addition, many of the compounds followed the Pfizer and GSK rules for drug-likeness (MW ≤ 400, logP ≤ 4), e.g., with **22b** having MW 397, a low logP value 2.98, HBB = 1, HBA = 7, RB = 8, and are predicted to have high Abbott Bioavailability Scores (55%) [100]. Compound **22a** demonstrates a low TPSA value 67.36 Å^2^ (TPSA < 75 Å^2^), indicating high blood–brain barrier (BBB) absorption, and is not predicted to inhibit the metabolic activity of CYP2D6 (see Supplementary Information Tables S2–S4 and Figures S1 and S2 for Brain Or Intestinal EstimateD permeation method (BOILED-Egg) WLOGP-*versus*-TPSA plot and Bioavailability Radar for triazoles **22a** and **22b** and imidazoles **23a** and **23b.** These molecules are predicted to have a high probability for passive absorption by the GI tract, are not substrates for P-gp, and relevant examples such as **22a**, **23a**, **23b**, **27a**, and **27b** have a high probability for brain penetration. Moderate aqueous solubility (e.g., in the range 11.2–22.9 μg/mL) was predicted for the most potent azole compounds **22a**, **22b**, **23a**, **23b**, **27a**, and **27b** (see Supplementary Information Tables S2 and S3 for details). The pK_a_H values for the most potent compound **22b** were calculated with Chemicalize [101] as 9.70 (phenol) and 2.18 (triazole) and were predicted to be ionized at physiological pH, while the pK_a_H values for **23b** were calculated as 9.71 (phenol) and 6.69 (imidazole).

The panel of azole compounds evaluated in the preliminary screening in MCF-7 breast cancer cells was also determined to be free from pan-assay interference compound (PAINS) alerts [102]. PAINS are compounds containing functional groups or fragments that contribute to high reactivity and would not be desirable for further progression and optimization. The Brenk filters were used to identify compounds that are potentially toxic chemically reactive metabolically unstable compounds or have poor pharmacokinetics [100] and did not identify any alerts for these compounds. Based on the phenotypic screening and Tier-1 profiling of their physicochemical and drug-like properties, the triazole compounds **22a** and **22b** were identified as suitable candidate compounds for additional in vitro cytotoxicity and biochemical investigation (See Supplementary Information Tables S2–S4).

### 3.7. Cytotoxicity in MCF-10A Cells

**Figure 9:**
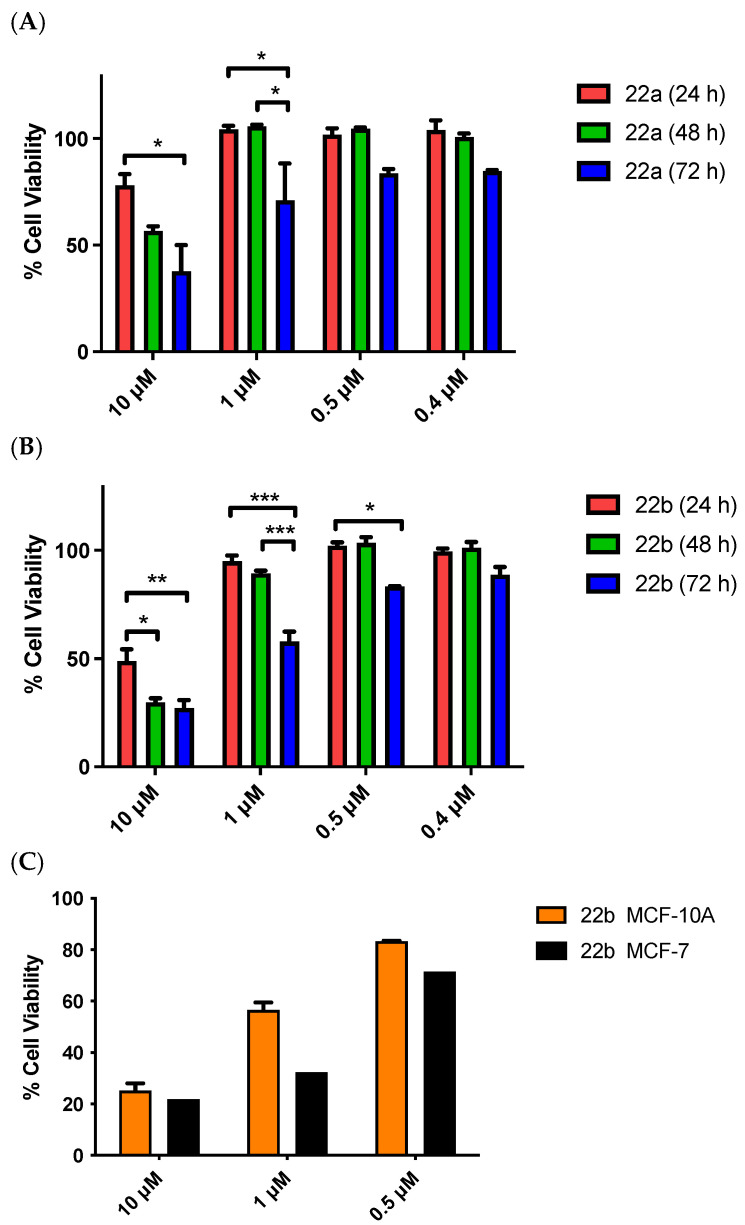
Effect of compounds **22a** (**A**) and **22b** (**B**) on the cell viability of non-tumorigenic MCF-10A human mammary epithelial cells at 24, 48, and 72 h. Cells were treated with the compounds **22a** and **22b** at concentrations of 10 μM, 1 μM, 0.5 μM, and 0.4 μM for 24, 48, or 72 h. (**C**) shows a comparison of the cell viability of MCF-10A cells and MCF-7 cells when treated with compound **22b** for 72 h at concentrations of 10 μM, 1 μM, and 0.5 μM. Cell viability was expressed as a percentage of vehicle control (ethanol 1% (*v*/*v*)) and was determined by an alamarBlue assay (average ± SEM of three independent experiments). Two-way ANOVA (Bonferroni post-test) was used to test for statistical significance (*, *p* < 0.05; **, *p* < 0.01; ***, *p* < 0.001).

MCF-10A is an immortalized human breast epithelial cell line derived from mastectomy tissue of fibrocystic disease [103]. These cells are widely used in toxicity studies as a control as they are structurally similar to normal human mammary epithelial cells [104]. MCF-10A are adherent, with characteristics of normal breast epithelium cells, i.e., non-tumorigenic in nude mice, with tridimensional growth in collagen, and their growth is controlled by hormones and growth factors [105]. In our studies, the MCF-10A cell line was used in the evaluation of the cytotoxicity of the novel compounds synthesized. The compounds selected (**22a** and **22b**) were tested at concentrations of 10, 1, 0.5, and 0.4 μM and at different time points (24, 48, 72 h) (Figure 9A,B). It was observed that the highest concentration (10 μM) of compound **22b** showed a cell death of approximately 50% at 24 h. Compound **22a** (10 μM) also had a higher percentage of viable cells (78%) but was less potent in MCF-7 cells. At 1 μM concentration, both compounds show 100% cell viability after 24 h. The percentage of viable cells at the highest concentration of 10 μM after 48 h decreased for both compounds to approximately 57% for compound **22a** and 30% for **22b**. The percentage of viable cells at 1 μM did not change significantly for each compound (>80%). The cell viability at 0.5 μM and 0.4 μM was close to 100%, indicating that the compound was not toxic to healthy cells. The third screening was at 72 h, which is the incubation time used for all the MCF-7 screenings (Figure 9). As the concentration of the compound decreases from 1 μM to 0.5 μM and 0.4 μM, the percentage of viable cells increased significantly, with >80% viability at 0.4 μM for **22a** and **22b**. This demonstrated that even at concentrations that would be toxic to the MCF-7 cancer cells, compound **22b** was not toxic to the MCF-10A cells and therefore possesses good selectivity and low cytotoxicity to normal cells. The most potent compound **22b** is less toxic to normal MCF-10A cells when compared to MCF-7 cells at the 72 h time point (Figure 9C). The MCF-7 cell viabilities at the 72 h time point are 20%, 30%, and 70% at 10 μM, 1 μM, and 0.5 μM concentrations, respectively. The corresponding cell viabilities for the MCF-10A cells at 72 h time point are 24%, 55%, and 80% at 10 μM, 1 μM, and 0.5 μM concentrations, respectively.

The low toxicity demonstrated by the triazole compound **22a** in the MCF-10A cells is also supported by the NCI 60-cell line 5-dose screen, with an LC_50_ value of 100 μM indicating the low toxicity of the compound over all 60 cell lines. Our results confirmed that azole **22b** was less toxic to normal human breast cells when compared with MCF-7 and MDA-MB-231 breast cancer cells and demonstrated potentially useful selectivity for development as an anticancer agent.

### 3.8. Cell Cycle and Pro-Apoptotic Effects of ***22b*** in MCF-7 and MDA-MB-231 Breast Cancer Cells

**Figure 10:**
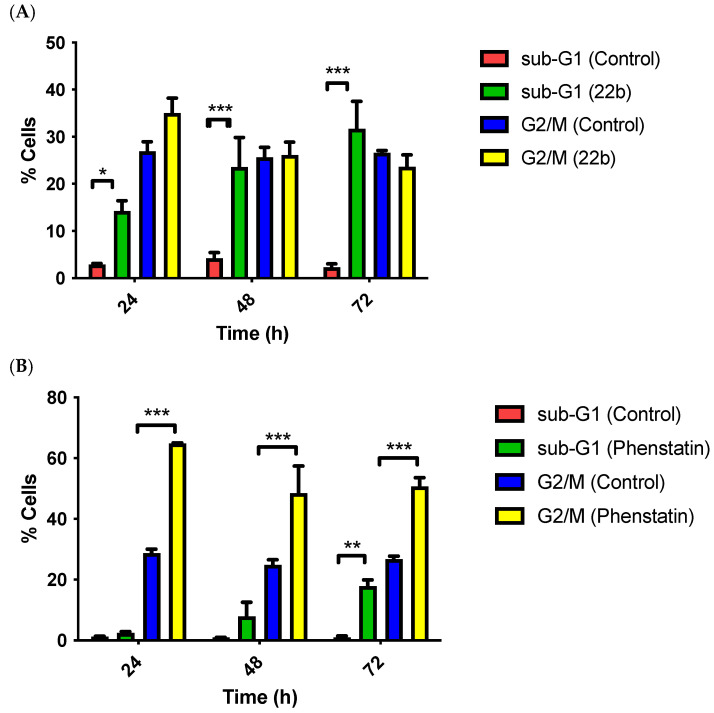
Compound (**A**) **22b**, (**B**) phenstatin **19c** induced apoptosis in a time-dependent manner in MCF-7 cells. Cells were treated with either vehicle control [0.1% ethanol (*v*/*v*)] or compound **22b** or phenstatin **19c** (1 μM) for 24, 48, and 72 h). The data shown for the control vehicle and phenstatin are as we previously reported [65]. Cells were fixed and stained with PI, followed by analysis using flow cytometry. Cell cycle analysis was performed on histograms of gated counts per DNA area (FL2-A). The number of cells with <2 N (sub-G_1_), 2 N (G_0_G_1_), and 4 N (G_2_/M) DNA content was determined with CellQuest software, BD CellQuest Pro. Values are represented as the mean ± SEM for three separate experiments. Two-way ANOVA (Bonferroni post-test) was used to test for statistical significance (*, *p* < 0.05; **, *p* < 0.01; ***, *p* < 0.001).

Cell cycle analysis allows measurement of the percentage of cells in each phase of the cell cycle at different time points; it is therefore an important tool in the investigation of the mechanism of action of drugs. Cell cycle analysis was determined in MCF-7 cells upon treatment with compound **22b**. There was an increase in cell death by apoptosis (sub-G_1_) observed at the three different time points 24, 48, and 72 h (14%, 23%, and 31%, respectively) compared to vehicle control (3%, 4%, and 2%, respectively) (Figure 10A). The percentage of cells in the G_2_/M phase for compound **22b** decreased from 35% to 26% to 23% at the relative time points of 24, 48, and 72 h corresponding to the increase in the population of cells undergoing apoptosis. It was observed that for phenstatin, the percentage of cells in apoptosis was very low at 24 and 48 h, only increasing to 18% at 72 h. The percentage of cells in the G_2_/M phase remained high at 24, 48, and 72 h time points with 65%, 57%, and 51% cells, respectively (Figure 10B). The data shown for the vehicle control and phenstatin are as we previously reported [65].

**Figure 11:**
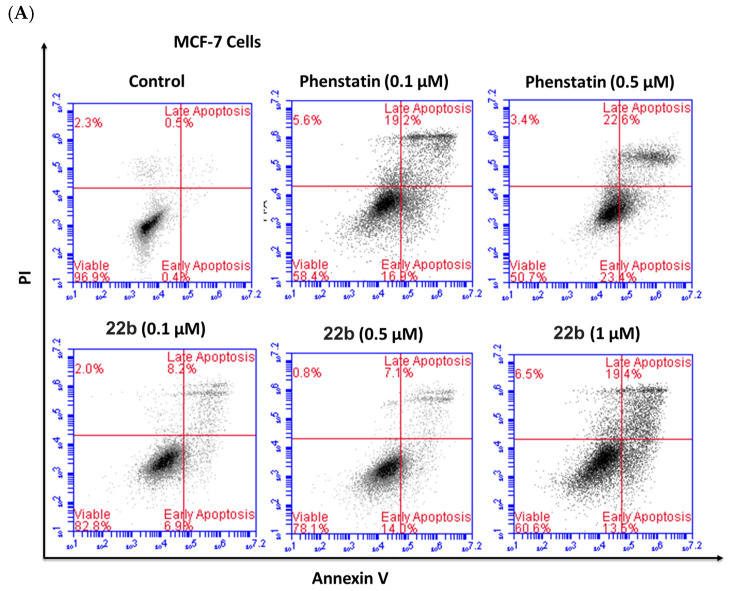

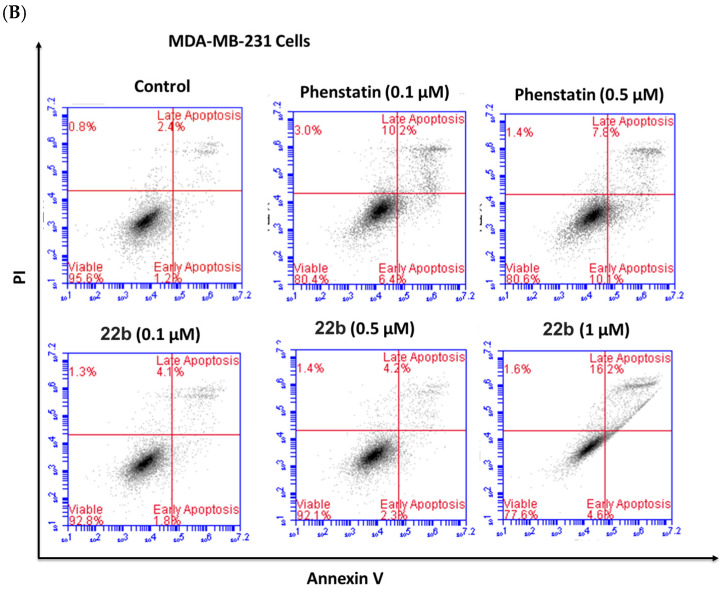
Compound **22b** induced apoptosis in (**A**) MCF-7 breast cancer cells and (**B**) MDA-MB-231 breast cancer cells. MCF-7 breast cancer cells (**A**) and MDA-MB-23 breast cancer cells (**B**) were treated with **22b** (0.1, 0.5, and 1.0 μM) or phenstatin (**19c**) (0.1 μM and 0.5 μM) or control vehicle (0.1% ethanol (*v*/*v*)). The data shown for the control vehicle and phenstatin are as we previously reported [65]. The apoptotic cell content was determined by staining with Annexin V-FITC and PI. In each panel, the lower right quadrant shows Annexin-positive cells in the early apoptotic stage and the upper right shows both Annexin/PI-positive cells in late apoptosis/necrosis. The lower left quadrant shows cells that are negative for both PI and Annexin V-FITC, and the upper left shows PI cells that are necrotic.

The pro-apoptotic effects of the triazole **22b** in MCF-7 and MDA-MB-231 cells were demonstrated by dual staining with annexin-V and propidium iodide (PI) (Figure 11), which is used to identify cells (annexin-V−/PI−), early apoptotic cells (annexin-V+/PI−), late apoptotic cells (annexin-V+/PI+), and necrotic cells (annexin-V−/PI+). It was observed that compound **22b** induced an increase in apoptosis (annexin-V positive cells) in a concentration-dependent manner (Figure 11A) in MCF-7 when compared to the vehicle (0.9%) and control phenstatin with 33% of cells undergoing apoptosis (early + late) at 1 μM concentration of **22b** and 21% at 0.5 μM. The control phenstatin (0.5 μM) induced apoptosis in 46% of the MCF-7 cells when examined at 72 h. In MDA-MB-231 cells, the percentage of cells observed in apoptosis following treatment with **22b** was considerably lower with 5.9%, 6.5%, and 20.8% at 0.1, 0.5, and 1.0 μM, respectively, as shown in Figure 11B. Total apoptosis for phenstatin was 36.1% (0.1 μM) and 46% (0.5 μM) in MCF-7 cells and 16.6% (0.1 μM) and 17.9% (0.5 μM) in MDA-MB-231 cells. These results indicate that the antiproliferative action of compound **22b** in MCF-7 cells could be attributed to its tubulin targeting effects, e.g., cell cycle G_2_/M arrest followed by apoptosis. The ability of the compound to inhibit tubulin polymerization was further examined.

**Figure 12:**
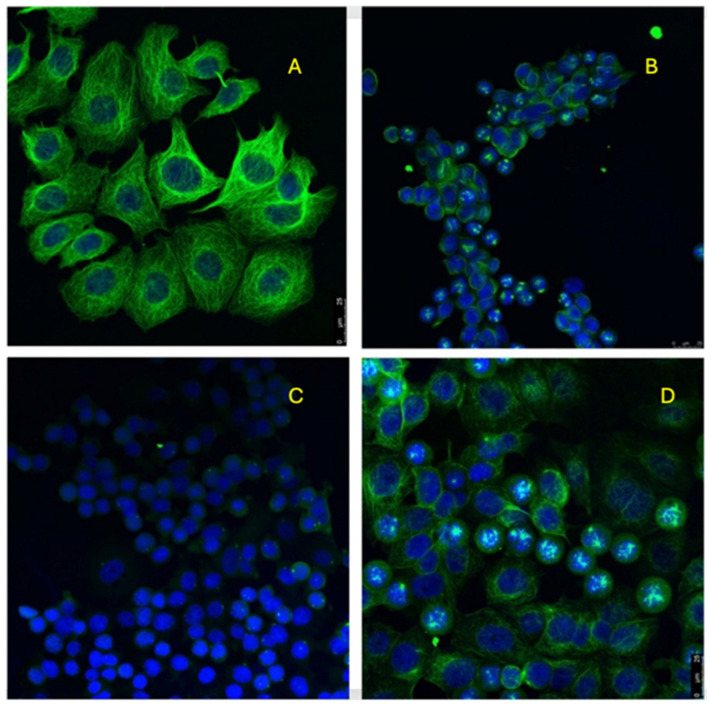
Compound **22b** depolymerizes the microtubule network of MCF-7 breast cancer cells. MCF-7 breast cancer cells were treated with (**A**) vehicle control [1% ethanol (*v*/*v*)], (**B**) paclitaxel (1 μM), (**C**) phenstatin (**19c**) (1 μM), or (**D**) compound **22b** (10 μM) for 16 h. Cells were preserved in ice-cold methanol and then stained with mouse monoclonal anti-α-tubulin–FITC–antibody (clone DM1A) (green), Alexa Fluor 488 dye, and counterstained with DAPI (blue). The micrograph images were obtained with Leica SP8 confocal microscopy utilizing Leica application suite X software. Representative confocal images of three separate experiments are shown. The scale bar indicates 25 μm.

The effects of compound **22b** on the microtubule structure of MCF-7 breast cancer cells were examined using confocal microscopy with anti-tubulin antibodies (Figure 12). Paclitaxel (tubulin polymerizer) and phenstatin (tubulin depolymerizer) were used as controls. The vehicle control (1% ethanol (*v*/*v*)) showed a well-organized microtubule network (stained green) around the cell nuclei (stained blue) Figure 12A. The paclitaxel-treated sample demonstrated the hyperpolymerization of tubulin (Figure 12B), while depolymerization of tubulin was observed in the phenstatin-treated sample (Figure 12C). Cells treated with the triazole **22b** (Figure 12D) displayed a disorganized microtubule network structure similar to that observed with phenstatin, together with multinucleation, indicative of mitotic catastrophe [106] as previously observed following treatment with tubulin-targeting agents, e.g., CA-4 in MCF-7 cells [107].

### 3.9. Inhibition of Tubulin Polymerization by Compound ***22b***

**Figure 13:**
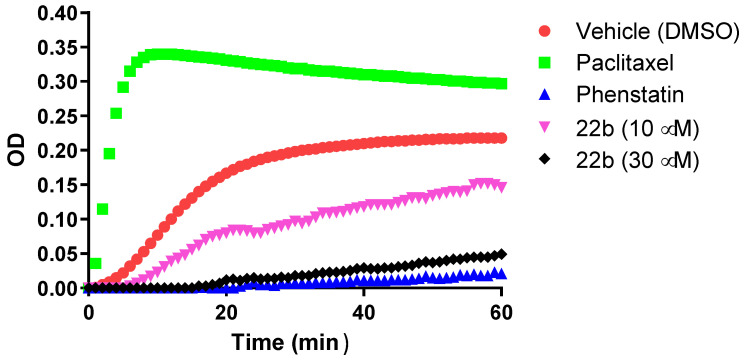
Inhibition of tubulin polymerization in vitro by compound **22b**. Tubulin polymerization assay for triazole compound **22b** at 10 μM and 30 μM concentration, together with control compounds paclitaxel (polymeriser) (10 μM) and phenstatin (depolymeriser) **19c** (10 μM). DMSO (1% *v*/*v*) was used in the vehicle control. Purified bovine tubulin and guanosine-5′-triphosphate (GTP) were initially mixed at 4 °C in a 96-well plate; the polymerization reaction was then initiated by warming the solution from 4 to 37 °C. The progress of the tubulin polymerization reaction at 37 °C was monitored at 340 nm in a Spectramax 340PC spectrophotometer at 30 s intervals for 60 min. Fold inhibition of tubulin polymerization can be calculated from the Vmax value for each reaction. The data shown for the control vehicle and phenstatin are as we previously reported [65].

Compound **22b** was selected for the tubulin polymerization assay as the lead compound for this study with antiproliferative activity (IC_50_ = 0.385 ± 0.12 μM) in MCF-7 cells. The structure of the A and B rings are similar to phenstatin and CA-4 and suggested that the mechanism of action of this compound could be antimitotic with the inhibition of tubulin polymerization. Following the protocol previously described [65], purified bovine brain tubulin was used for the assay, and its polymerization was determined spectrophotometrically. The light scattered is directly proportional to the concentration of polymerized microtubules produced in the assay, and the change in turbidity is determined (Figure 13). Paclitaxel (10 μM) was used as a control [108]. Compound **22b** at 30 μM concentration (black) and at 10 μM (pink) showed good inhibition of tubulin polymerization after 60 min, corresponding to a 1.5-fold reduction in the polymer mass at 10 μM, compared to the vehicle [1% DMSO (*v*/*v*)] and a 5-fold reduction–reduction at 30 μM concentration. This compares with 10-fold reduction for phenstatin (10 μM).

### 3.10. Aromatase Inhibition by Compound ***22b***

An objective of this research was to establish if it was possible to combine the known anti-tubulin activity of chalcone and CA4 scaffolds with the aromatase inhibition activity demonstrated by azoles such as triazoles and imidazole to create a hybrid compound with both anti-tubulin and anti-aromatase activity. The potential of the most potent antiproliferative hybrid compound synthesized (**22b**) as a dual-acting tubulin/aromatase inhibitor was next evaluated against two members of the cytochrome P450 family: CYP19 and CYP1A1 [109]. CYP19 is the aromatase cytochrome, which is responsible for the formation of endogenous estradiol by aromatization of testosterone and androstenedione. CYP1A1 is involved in the biotransformation and degradation of estrogen [110]. The specificity of aromatase inhibition of the triazole **22b** was determined in an assay using the xenobiotic and drug-metabolising cytochrome P450 enzymes CYP1A1. The determination of the aromatase activity of the compound is based on the detection of hydrolyzed dibenzylfluorescein (DBF) by the aromatase enzyme [111]. Both aromatase and CYP1A1 inhibition activities were determined from the fluorescent intensity of fluorescein, the hydrolysis product of dibenzylfluorescein (DBF) by aromatase as previously described [112,113]. The flavanone naringenin [114] was used as a positive control, with an IC_50_ value of 4.9 μM determined for aromatase inhibition.

Compound **22b** was found to be a potent inhibitor of the cytochrome CYP19 with inhibition of 93%, based on the result of the one-dose evaluation (20 µg/mL, 50 μM). The inhibition for compound **22b**, although potent, was not concentration-dependent and the IC_50_ could not be determined. The specificity of aromatase inhibition was determined with the xenobiotic-metabolizing cytochrome P450 enzymes CYP1A1. Compound **22b** did not show significant inhibitory activity of CYP1A1, and the IC_50_ value above 53 µM was determined, which is regarded as inactive [113,115]. From the results obtained and by comparison with our previously reported related compounds based on the phenstatin scaffold [65], the 1,2,4-triazole-containing chalcone-based compound **22b** was identified as a potential dual-acting drug for the treatment of breast cancer targeting both aromatase inhibition and tubulin polymerization.

### 3.11. Molecular Docking of Hybrids

**Figure 14:**
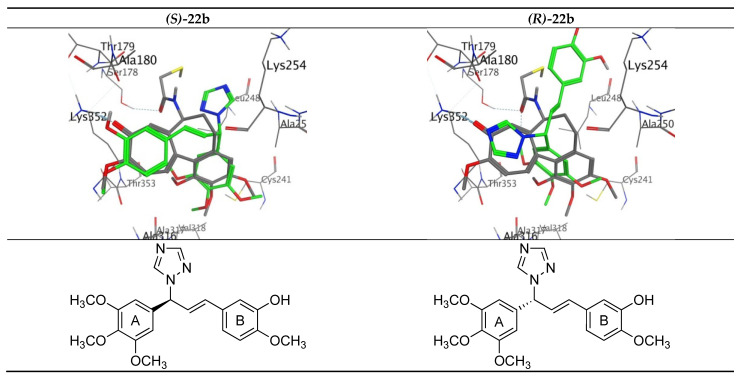
Docking of compounds **22b** in the colchicine binding site of tubulin. Overlay of the X-ray structure of tubulin co-crystallized with DAMA-colchicine (PDB entry 1SA0, [116]) on the best-ranked docked poses of *(S)-***22b** and *(R)-***22b**. Ligands are rendered as tubes and amino acids as lines. Tubulin amino acids and DAMA-colchicine are colored by atom type; the novel compounds are colored green. The atoms are colored by element type, carbon = grey, hydrogen = white, oxygen = red, nitrogen = blue, sulfur = yellow. Key amino acid residues are labeled, and multiple residues are hidden to enable a clearer view.

Compound **22b** was examined in tubulin molecular docking experiments. Compound **22b** was obtained in the synthetic study as a racemate. As it was of interest to examine the effect of stereochemistry on potential tubulin binding, both *R* and *S* enantiomers were docked in the crystallized tubulin structure 1SA0 [116]; docking calculations were undertaken using MOE 2016.0802 [117] (Figure 14). The co-crystallized tubulin DAMA-colchicine structure 1SA0 was used for this study as it has been reported that both CA4 and phenstatin interact with tubulin at the colchicine binding site [118]. Compound *(S)-***22b** overlays the B-ring on the C-ring of DAMA-colchicine (forming HBA interactions with Lys352); the compound co-locates the 3,4,5-trimethoxyphenyl substituted A-ring and positions the heterocycle in an open region of the tubulin binding site. A similar alignment is not observed for *(R)-***22b**, recapitulating the interactions of the colchicine core but is unable to make an HBA with Ser178. The predicted affinity ranking is *(S)-***22b** > *(R)-***22b** (docking scores: −8.75 vs. −8.31). *(S)-***22b** maintains the typical colchicine mapping binding pose with the triazole sidechain directed toward the Ser178/Leu248 pocket. However, the best-ranked docked pose of the *(R)-***22b** enantiomer maps positions the triazole ring on the C-ring of colchicine as shown in Figure 14. (See also Supplementary Information, Figure S19 for overlay of imidazole-chalcones with letrozole and phenstatin). This result provides confirmation of the observed biochemical experiments in which cell cycle and tubulin binding were demonstrated and indicates that these novel compounds are pro-apoptotic and inhibit tubulin polymerization. Further studies to provide enantiomerically pure compounds will allow the identification of the more potent enantiomer and investigation of the stereoselective effects of the compounds in breast cancer cells.

## 4. Materials and Methods

### 4.1. Chemistry

Melting points were measured on a Gallenkamp SMP 11 melting point apparatus and were uncorrected. Infra-red (IR) spectra were recorded as a thin film on NaCl plates, or as potassium bromide discs on a Perkin Elmer FT-IR Spectum 100 spectrometer (Waltham, MA, USA). ^1^H and ^13^C nuclear magnetic resonance (NMR) spectra were recorded at 27 °C on a Bruker Avance DPX 400 spectrometer (Billerica, MA, USA) (400.13 MHz, ^1^H; 100.61 MHz, ^13^C) at 20 °C in CDCl_3_ (internal standard tetramethylsilane TMS) or DMSO-*d*_6_. For CDCl_3_, ^1^H-NMR spectra were assigned relative to the TMS peak at δ 0.00 and ^13^C-NMR spectra relative to the CDCl_3_ triplet (77.00 ppm). Electrospray ionization mass spectrometry (ESI-MS) was determined on a liquid chromatography time-of-flight (TOF) mass spectrometer (Micromass LCT, Waters Ltd., Manchester, UK) with the electrospray ionization (ES) interface operated in the positive ion mode. High Resolution Mass (HRMS) measurement accuracies are <±5 ppm. R_f_ values are for thin layer chromatography (TLC) on silica gel Merck F-254 plates. Flash column chromatography was performed on Merck Kieselgel 60 (particle size 0.040–0.063 mm) and on the Biotage SP4 instrument. All products isolated were homogenous on TLC. Analytical high-performance liquid chromatography (HPLC) for purity determination of products was performed using a Waters 2487 Dual Wavelength Absorbance detector, Waters 1525 binary HPLC pump, Waters In-Line Degasser AF, and Waters 717plus Autosampler and Varian Pursuit XRs C18 reverse phase 150 × 4.6 mm chromatography column with detection at 254 nm. Chalcones **20a–h**, **20j**, **20k**, **31a**, **31b,** alcohols **21a–c,** and indenol **25a** were prepared following the reported procedures [39,44,79,119,120,121,122,123,124], see Supplementary Information for details.

(*E*)-3-(4-Methoxy-3-nitrophenyl)-1-(3,4,5-trimethoxyphenyl)prop-2-en-1-one (20i). 3,4,5-trimethoxyacetophenone was added to a solution of 4-methoxy-3-nitrobenzaldehyde (1 eq, 7.14 mmol, 1.29 g) in methanol (20 mL) containing KOH (50%, 10 mL) (1 eq, 7.14 mmol, 1.5 g) (1 eq) while stirring at 20 °C. After 24 h, water and HCl (10%) were added to complete the precipitation. The precipitated product was filtered and recrystallized from methanol. Yield: 78%, 2.0 g, yellow solid, Mp. 147–149 °C. IR: ν_max_ (ATR) cm^−1^: 3279, 1650, 1577, 1528, 1458, 1351, 1271, 1117, 1002, 808. ^1^H NMR (400 MHz, CDCl_3_) δ 3.94 (s, 3 H, OCH_3_), 3.96 (s, 6 H, 2×OCH_3_), 4.02 (s, 3 H, OCH_3_), 7.14 (d, *J* = 8.7 Hz, 1 H, Ar-H), 7.27 (s, 2 H, Ar-H), 7.43 (d, *J* = 15.8 Hz, 1 H, CH=CH), 7.75 (d, *J* = 15.8 Hz, 1 H, CH=CH), 7.79 (d, *J* = 2.1 Hz, 1 H, Ar-H), 8.16 (d, *J* = 2.5 Hz, 1 H, Ar-H). ^13^C NMR (101 MHz, CDCl_3_) 56.45 (2×OCH_3_), 56.76 (OCH_3_), 60.99 (OCH_3_), 106.13 (2×CH), 113.84 (CH), 121.91 (CH=CH, CH), 124.65 (C), 127.62 (C), 133.10 (CH), 134.49 (C-NO_2_), 141.61 (CH=CH), 142.79 (C-O), 153.20 (C-O), 154.11 (2×C-O), 188.40 (C=O). HRMS (EI): Found 396.1062 [M+Na]^+^; C_19_H_19_NNaO_7_ requires 396.1059.

#### 4.1.1. General Method I: Preparation of (*E*)-1,3-Diarylprop-2-en-1-ols (**21a–i**)

To a solution of the appropriate chalcone (1 eq) in methanol (25 mL), a suspension of sodium borohydride NaBH_4_ (1 eq) in methanol (10 mL) and THF (10 mL) was slowly added. The reaction mixture was stirred (0–20 °C) and monitored by TLC until the reaction was complete. NaHCO_3_ (sat., 5 mL) was then added and the reaction mixture was concentrated. The reaction residue was extracted with ethyl acetate, washed with water and brine, and dried over sodium sulfate. No further purification was required.

**(*E*)-3-(3,4-Dimethoxyphenyl)-1-(3,4,5-trimethoxyphenyl)prop-2-en-1-ol (21d):** As per general method I, a solution of (*E*)-3-(3,4-dimethoxyphenyl)-1-(3,4,5-trimethoxyphenyl)prop-2-en-1-one (**20d**) (1 eq, 2.79 mmol, 1.0 g) in methanol (25 mL) was treated with a suspension of NaBH_4_ (2 eq, 5.58 mmol, 0.21 g) in methanol (10 mL) and THF (10 mL). The product was isolated as a yellow solid, yield: 97%, 0.98 g, Mp. 50–53 °C. IR: ν_max_ (ATR) cm^−1^: 2936, 2835, 1583, 1506, 1458, 1416, 1261, 1230, 1121, 1023, 1002, 965, 807, 764, 700. ^1^H NMR (400 MHz, CDCl_3_) δ 3.86 (s, 3 H, OCH_3_), 3.89 (s, 6 H, 2×OCH_3_), 3.89 (s, 3 H, OCH_3_), 3.90 (s, 3 H, OCH_3_), 5.32 (dd, *J* = 6.6, 2.5 Hz, 1 H, CH-OH), 6.24 (dd, *J* = 15.8, 6.6 Hz, 1 H, CH=CH), 6.63 (d, *J* = 15.8 Hz, 1 H, CH=CH), 6.68 (s, 2 H, Ar-H), 6.83 (d, *J* = 8.7 Hz, 1 H, Ar-H), 6.93–6.95 (m, 1 H, Ar-H), 6.95–6.97 (m, 1 H, Ar-H). ^13^C NMR (101 MHz, CDCl_3_) 55.84 (OCH_3_), 55.92 (OCH_3_), 56.16 (2×OCH_3_), 64.12 (OCH_3_), 75.38 (CH-OH), 103.13 (2×CH), 108.92 (CH), 111.07 (CH), 119.97 (CH), 129.30 (C), 129.44 (CH=CH), 130.68 (CH=CH), 133.83 (C), 138.67 (C-O), 153.39 (2×C-O) ppm. HRMS (EI): Found 343.1548 [M-OH]^+^; C_20_H_23_O_5_ requires 343.1546.

**(*E*)-3-(4-Ethoxyphenyl)-1-(3,4,5-trimethoxyphenyl)prop-2-en-1-ol (21e):** As per general method I (*E*)-3-(4-ethoxyphenyl)-1-(3,4,5-trimethoxyphenyl)prop-2-en-1-one (**20e**) (1 eq, 2.92 mmol, 1.0 g) was reacted with sodium borohydride (2 eq, 5.84 mmol, 0.22 g) in methanol (10 mL) and THF (10 mL). The product was isolated as a yellow oil, yield: 87%, 0.87 g. IR: ν_max_ (ATR) cm^−1^: 2993, 2936, 1581, 1505, 1450, 1416, 1230, 1119, 1043, 966, 823, 807. ^1^H NMR (400 MHz, CDCl_3_) δ 1.41 (t, *J* = 7.0 Hz, 3 H, CH_3_) 3.85 (s, 3 H, OCH_3_) 3.87 (s, 6 H, 2×OCH_3_) 4.03 (q, *J* = 7.1 Hz, 2 H, CH_2_) 5.29 (d, *J* = 6.2 Hz, 1 H, CH-OH) 6.23 (dd, *J* = 15.8, 7.1 Hz, 1 H, CH=CH) 6.62 (d, *J* = 16.2 Hz, 1 H, CH=CH) 6.66 (s, 2 H, Ar-H) 6.82–6.87 (m, 2 H, Ar-H) 7.30–7.35 (m, 2 H, Ar-H). ^13^C NMR (101 MHz, CDCl_3_) 14.76 (CH_3_) 56.08 (OCH_3_) 56.10 (OCH_3_) 60.78 (OCH_3_) 63.44 (CH_2_) 75.40 (CH-OH) 103.09 (2×CH) 114.52 (2×CH) 127.80 (2×CH, CH=CH) 128.98 (CH=CH) 130.41 (C) 137.29 (C) 138.78 (C-O) 153.32 (2×C-O) 158.77 (C-OEt) ppm. HRMS (EI): Found 343.1560 [M-H]^+^; C_20_H_22_O_5_ requires 343.1546.

**(*E*)-3-(4-Fluorophenyl)-1-(3,4,5-trimethoxyphenyl)prop-2-en-1-ol (21f):** As per general method I (*E*)-3-(4-fluorophenyl)-1-(3,4,5-trimethoxyphenyl)prop-2-en-1-one (**20f**) (1 eq, 3.1 mmol, 1.0 g) was reacted with sodium borohydride (2 eq, 6.3 mmol, 0.24 g) in methanol (10 mL) and THF (10 mL). The product was isolated as a yellow oil, yield: 100%, 0.98 g. IR: ν_max_ (ATR) cm^−1^: 2942, 2837, 1695, 1597, 1524, 1462, 1422, 1312, 1248, 1128, 1093, 1026, 1003, 954, 926, 854, 825, 813, 758, 688. ^1^H NMR (400 MHz, CDCl_3_) δ 3.85 (s, 3 H, OCH_3_), 3.88 (s, 6 H, 2×OCH_3_), 5.32 (dd, *J* = 6.2, 2.5 Hz, 1 H, CH-OH), 6.29 (dd, *J* = 15.8, 6.2 Hz, 1 H, CH=CH), 6.63–6.69 (m, 3 H, 2×CH, CH=CH), 6.98–7.04 (m, 2 H, Ar-H), 7.35–7.40 (m, 2 H, Ar-H). ^13^C NMR (101 MHz, CDCl_3_) 56.15 (2×OCH_3_), 60.83 (OCH_3_), 75.21 (CH-OH), 103.15 (2×CH), 115.40 (CH), 115.61 (CH), 128.10 (CH=CH), 128.19 (2×CH), 129.47 (CH=CH), 131.00 (C), 132.57 (C), 138.45 (C-O), 153.43 (2×C-O), 163.67 (C-F) ppm. HRMS (EI): Found 317.1198 [M-H]^+^; C_18_H_18_FO_4_ requires 317.1189.

**(*E*)-3-Phenyl-1-(3,4,5-trimethoxyphenyl)prop-2-en-1-ol (21g):** As per general method I (*E*)-3-phenyl-1-(3,4,5-trimethoxyphenyl)prop-2-en-1-one (**20g**) (1 eq, 3.35 mmol, 1.0 g) was reacted with sodium borohydride (2 eq, 6.7 mmol, 0.25 g) in methanol (25 mL) and THF (25 mL). The product was isolated as pale yellow solid, yield: 85% (0.85 g), Mp: 82–85 °C. IR: ν_max_ (ATR) cm^−1^: 3328, 2995, 2827, 1591, 1507, 1462, 1420, 1234, 1124, 1001, 962, 822, 757. ^1^H NMR (400 MHz, CDCl_3_) δ 7.32–7.28 (m, 5H, Ar-H), 6.68 (d, *J* = 16.0 Hz, 1H, CH=CH), 6.65 (d, *J* = 1.1 Hz, 2H, Ar-H), 6.50 (dd, *J* = 15.8, 3.9 Hz, 1H, CH=CH), 5.31 (d, *J* = 6.5 Hz, 1H, CH), 3.86 (s, 6H, 2×OCH_3_), 3.85 (s, 3H, OCH_3_). ^13^C NMR (101 MHz, CDCl_3_) 153.37 (2×C), 136.78 (C), 136.46 (C), 131.55 (C), 130.15 (CH=CH), 128.56 (2×CH), 127.84 (CH), 126.60 (2×CH), 103.93 (2×CH), 75.24 (CH), 60.81 (OCH_3_), 56.09 (2×OCH_3_) ppm.

**(*E*)-3-(4-Nitrophenyl)-1-(3,4,5-trimethoxyphenyl)prop-2-en-1-ol (21 h)**: As per general method I, (*E*)-3-(4-nitrophenyl)-1-(3,4,5-trimethoxyphenyl)prop-2-en-1-one (**20h**) (1 eq, 2.91 mmol, 1.0 g) was reacted with sodium borohydride (2 eq, 5.83 mmol, 0.22 g) in methanol (25 mL) and THF (25 mL). The product was isolated as a brown solid, yield: 91%, 0.910 g, Mp. 146–150 °C. IR: ν_max_ (ATR) cm^−1^: 3396, 2938, 2836, 1593, 1510, 1462, 1448, 1335, 1236, 1131, 1106, 1008, 864, 833, 820, 694. ^1^H NMR (400 MHz, CDCl_3_) δ 3.84 (s, 3 H, OCH_3_), 3.88 (s, 6 H, 2×OCH_3_), 5.36 (m, 1 H, CH-OH), 6.53 (dd, *J* = 16.2 Hz, 1 H, CH=CH), 6.64 (s, 2 H, Ar-H), 6.78 (d, *J* = 16.2 Hz, 1 H, CH=CH), 7.53 (m, *J* = 8.7 Hz, 2 H, Ar-H), 8.18 (m, *J* = 8.7 Hz, 2 H, Ar-H). ^13^C NMR (101 MHz, CDCl_3_) 56.19 (2×OCH_3_) 60.84 (OCH_3_), 74.85 (CH), 103.25 (2×CH), 124.00 (2×CH), 127.12 (CH, CH=CH), 127.91 (CH, CH=CH), 135.96 (C), 137.74 (C-O), 143.02 (C), 147.04 (C-NO_2_), 153.57 (2×C-O) ppm. HRMS (EI): Found 344.1137 [M-H]^+^; C_18_H_18_NO_6_ requires 344.1134.

**(*E*)-3-(4-Methoxy-3-nitrophenyl)-1-(3,4,5-trimethoxyphenyl)prop-2-en-1-ol (21i)**: As per general method I, (*E*)-3-(4-methoxy-3-nitrophenyl)-1-(3,4,5-trimethoxyphenyl)prop-2-en-1-one (**20i**) (1 eq, 2.68 mmol, 1 g) was reacted with sodium borohydride (2 eq, 5.36 mmol, 0.203 g) in methanol (25 mL) and THF (25 mL). The product was isolated as a brown oil, yield: 94%, 0.941 g,. IR: ν_max_ (ATR) cm^−1^: 3404, 2940, 2840, 1618, 1591, 1527, 1502, 1417, 1350, 1265, 1231, 1121, 1005, 966, 814, 733, 700, 664. ^1^H NMR (400 MHz, CDCl_3_) δ 3.84 (s, 3 H, OCH_3_), 3.87 (s, 6 H, 2×OCH_3_), 3.95 (s, 3 H, OCH_3_), 5.31 (d, *J* = 7.5 Hz, 1 H, CH-OH), 6.31 (dd, *J* = 15.8, 6.2 Hz, 1 H, CH=CH), 6.58 (d, *J* = 18.2 Hz, 1 H, CH=CH), 6.63 (s, 2 H, Ar-H), 7.03 (d, *J* = 8.7 Hz, 1 H, Ar-H), 7.54 (dd, *J* = 8.7, 2.1 Hz, 1 H, Ar-H), 7.86 (d, *J* = 2.1 Hz, 1 H, Ar-H). ^13^C NMR (101 MHz, CDCl_3_) 56.10 (2×OCH_3_), 56.59 (OCH_3_), 60.79 (OCH_3_), 74.88 (CH-OH), 103.09 (2×CH), 113.60 (CH), 123.41 (CH), 125.28 (CH=CH), 127.46 (C), 129.43 (CH=CH), 132.00 (C), 132.24 (CH), 137.88 (C-O), 138.22 (C-NO_2_), 152.26 (C-O), 153.42 (2×C-O) ppm. HRMS (EI): Found 374.1245 [M-H]^+^; C_19_H_20_NO_7_ requires 374.1240.

**(*E*)-3-(4-Chlorophenyl)-1-(3,4,5-trimethoxyphenyl)prop-2-en-1-ol (21j):** As per general method I, (*E*)-3-(4-chlorophenyl)-1-(3,4,5-trimethoxyphenyl)prop-2-en-1-one (**20l**) (0.9 mmol, 300 mg) was treated with sodium borohydride (2 equiv) in MeOH:THF (1:1) and allowed to stir for 1 h to afford the pure product as a white powder (90%) [125] ^1^H NMR (400 MHz, DMSO-*d*_6_): δ 7.46 (d, *J* = 8.5 Hz, 2 H), 7.35 (d, *J* = 8.5 Hz, 2 H), 6.69 (s, 2H), 6.62 (d, *J* = 15.8 Hz, 1 H), 6.43 (dd, *J* = 15.8, 6.1 Hz, 1 H), 5.62 (d, *J* = 4.2 Hz, 1 H), 5.17 (t, 1 H), 3.76 (s, 6 H), 3.62 (s, 3 H). ^13^C NMR (400 MHz, DMSO-*d*_6_): 152.72, 140.01, 136.32, 135.65, 134.54, 131.65, 128.52, 128.01, 126.64, 103.26, 73.13, 59.94, 55.78 ppm.

#### 4.1.2. General Method II: Preparation of Series 1 (*E*)-1-(1,3-Diarylallyl)-1*H*-1,2,4-Triazoles (**22a–g**)

1,2,4-triazole (3 eq) and *p*-toluenesulfonic acid (200 mg, 0.61 eq) were added to a solution of the appropriate (*E*)-1,3-diarylprop-2-en-1-ol (**21a–21g**) (1 eq) in toluene (60 mL). The reaction mixture was heated at reflux for 4 h in a Biotage open vessel microwave reactor (90–250 W) equipped with a Dean-Stark trap. When the reaction was complete, the toluene was evaporated. The crude product was then dissolved in ethyl acetate (30 mL) and washed with water (20 mL) and brine (10 mL). The solution was dried over sodium sulfate, filtered, and concentrated under reduced pressure. The crude product was purified by flash chromatography over silica gel to give the desired product.

**(*E*)-1-(3-(4-Methoxyphenyl)-1-(3,4,5-trimethoxyphenyl)allyl)-1*H*-1,2,4-triazole (22a):** As per general method II, (*E*)-3-(4-methoxyphenyl)-1-(3,4,5-trimethoxyphenyl)prop-2-en-1-ol (**21a**) (1 eq, 1.5 mmol, 0.5 g) was reacted with 1,2,4-triazole and *p*-TSA in toluene. The crude product was purified via flash chromatography (eluent: ethyl acetate/*n*-hexane/methanol 10:1:2) over silica gel to afford the desired product as a yellow oil. Yield: 37%, 0.212 g. IR: ν_max_ (ATR) cm^−1^: 3117, 2937, 2837, 1605, 1592, 1583, 1507, 1461, 1417, 1330, 1273, 1242, 1176, 1122, 1004, 957, 860, 823, 796, 776, 667, 663. ^1^H NMR (600 MHz, CDCl_3_) δ 3.82 (br. s., 3 H, OCH_3_), 3.85 (s, 3 H, OCH_3_), 3.87 (s, 6 H, 2×OCH_3_), 6.15 (d, *J* = 6.8 Hz, 1 H, CH-N-R), 6.36 (d, *J* = 15.8 Hz, 1 H, CH=CH), 6.56 (dd, *J* = 15.8, 6.4 Hz, 1 H, CH=CH), 6.61 (s, 2 H, Ar-H), 6.93–6.96 (m, 2 H, Ar-H), 7.34–7.37 (m, 2 H, Ar-H), 8.09 (s, 1 H, CH-N), 8.18 (s, 1 H, CH-N). ^13^C NMR (101 MHz, CDCl_3_) 55.31 (OCH_3_), 56.19 (2×OCH_3_), 60.92 (OCH_3_), 65.49 (CH-N-R), 104.50 (2×CH), 114.48 (2×CH), 123.03 (CH=CH), 128.97 (C), 133.54 (CH=CH), 134.06 (2×CH), 134.36 (C), 138.18 (C), 142.63 (CH-N), 152.16 (CH-N), 153.68 (2×C-O), 159.90 (C-O) ppm. HRMS (EI): Found 416.1373 [M+Cl]^+^; C_21_H_23_^35^ClN_3_O_4_ requires 416.1377.

**(*E*)-5-(3-(1*H*-1,2,4-Triazol-1-yl)-3-(3,4,5-trimethoxyphenyl)prop-1-en-1-yl)-2-methoxyphenol (22b):** As per general method II, (*E*)-5-(3-hydroxy-3-(3,4,5-trimethoxyphenyl)prop-1-en-1-yl)-2-methoxyphenol (**21b**) (1 eq, 0.75 mmol, 0.26 g) was reacted with 1,2,4-triazole and *p*-TSA in toluene. The crude product was purified via flash chromatography (eluent: ethyl acetate/methanol 10:0.5) over silica gel to afford the desired product as an orange resin; yield: 34%, 0.1 g, IR: ν_max_ (ATR) cm^−1^: 2999, 2937, 2838, 1583, 1504, 1459, 1418, 1329, 1272, 1237, 1122, 1003, 860, 802, 762, 731, 670. ^1^H NMR (400 MHz, CDCl_3_) δ 8.15 (s, 1H, CH-N), 8.09 (s, 1 H, CH-N), 7.18 (s, 1 H, Ar-H), 7.02 (m, *J* = 2.7 Hz, 1 H, Ar-H), 6.83 (s, 1 H, Ar-H), 6.58 (s, 2 H, Ar-H), 6.52 (dd, *J* = 8.5, 4.5 Hz, 1 H, CH), 6.38 (d, *J* = 3.4 Hz, 1 H, CH), 6.05 (s, 1 H, CH-N-R), 3.88 (s, 3 H, OCH_3_), 3.84 (s, 6 H, 2×OCH_3_), 3.79 (s, 3 H, OCH_3_). ^13^C NMR (101 MHz, CDCl_3_) 153.33 (2×C-O), 151.92 (CH), 148.45 (C-O), 146.95 (C-OH), 142.54 (CH), 138.48 (C-O), 134.19 (C), 133.42 (C), 131.21 (CH=CH), 125.18 (CH=CH), 119.41 (CH), 113.86 (CH), 112.13 (CH), 103.91 (2×CH), 65.63 (CH-N-R), 60.91 (OCH_3_), 56.16 (OCH_3_), 56.12 (2×OCH_3_) ppm. HRMS (EI): Found 396.1565 [M-H]^+^; C_21_H_22_N_3_O_5_ requires 396.1560.

**(*E*)-1-(1,3-Bis(3,4,5-trimethoxyphenyl)allyl)-1*H*-1,2,4-triazole (22c):** As per general method II (*E*)-1,3-bis(3,4,5-trimethoxyphenyl)prop-2-en-1-ol (**21c**) (1 eq, 0.76 mmol, 0.3 g) in toluene (60 mL) was reacted with 1,2,4-triazole and *p*-TSA in toluene. The crude product was purified via flash chromatography (eluent: ethyl acetate/*n*-hexane 9:1) over silica gel to afford the desired product as a white solid. Yield: 30%, 0.098 g, Mp. 174–177 °C. IR: ν_max_ (ATR) cm^−1^: 2942, 1582, 1456, 1421, 1332, 1241, 1203, 1122, 1000, 969, 819, 683, 665. ^1^H NMR (400 MHz, CDCl_3_) δ 3.84 (s, 6 H, 2×OCH_3_), 3.86 (s, 3 H, OCH_3_), 3.86 (s, 3 H, OCH_3_), 3.88 (s, 6 H, 2×OCH_3_), 6.11 (d, *J* = 6.6 Hz, 1 H, CH-N-R), 6.41 (d, *J* = 16.0 Hz, 1 H, CH=CH), 6.49 (s, 2 H, Ar-H), 6.56 (dd, *J* = 16.0, 4.0 Hz, 1 H, CH=CH), 6.62 (s, 2 H, Ar-H), 8.06 (s, 1 H, CH-N), 8.18 (s, 1 H, CH-N). ^13^C NMR (101 MHz, CDCl_3_) 56.15 (2×OCH_3_), 56.20 (2×OCH_3_), 60.81 (OCH_3_), 60.91 (OCH_3_), 66.11 (CH-N-R), 103.99 (2×CH), 104.57 (2×CH), 124.81 (CH=CH), 131.03 (CH=CH), 133.16 (C), 134.64 (C), 138.23 (C-O), 138.64 (C-O), 142.71 (CH), 152.19 (CH), 153.38 (2×C-O), 153.68 (2×C-O) ppm. HRMS (EI): Found 440.1855 [M-H]^+^; C_23_H_26_N_3_O_6_ requires 440.1822.

**(*E*)-1-(3-(3,4-Dimethoxyphenyl)-1-(3,4,5-trimethoxyphenyl)allyl)-1*H*-1,2,4-triazole (22d):** As per general method II (*E*)-3-(3,4-dimethoxyphenyl)-1-(3,4,5-trimethoxyphenyl)prop-2-en-1-ol (**21d**) (1 eq, 1.23 mmol, 0.44 g) was reacted with 1,2,4-triazole and *p*-TSA in toluene. The crude product was purified via flash chromatography (eluent: ethyl acetate/*n*-hexane 9:1) over silica gel to afford the desired product as a yellow oil. Yield: 46%, 0.23g. IR: ν_max_ (ATR) cm^−1^: 2937, 2836, 1583, 1505, 1460, 1418, 1329, 1262, 1236, 1122, 1023, 1005, 803, 766, 677. ^1^H NMR (400 MHz, CDCl_3_) δ 3.87 (s, 6 H, 2×OCH_3_), 3.89 (s, 3 H, OCH_3_), 3.90 (s, 6 H, 2×OCH_3_), 6.10 (d, *J* = 6.2 Hz, 1 H, CH-N-R), 6.36 (dd, *J* = 15.8, 1.2 Hz, 1 H, CH=CH), 6.47 (s, 1 H, Ar-H), 6.53–6.60 (m, 1 H, CH=CH), 6.61 (s, 2 H, Ar-H), 6.80 (d, *J* = 2.1 Hz, 1 H, Ar-H), 6.88 (d, *J* = 2.1 Hz, 1 H, Ar-H), 8.12 (s, 1 H, CH-N), 8.18 (s, 1 H, CH-N). ^13^C NMR (101 MHz, CDCl_3_) 56.16 (4×OCH_3_), 60.88 (OCH_3_), 66.29 (CH-N-R), 104.48 (2×CH), 110.68 (CH), 111.29 (CH), 120.22 (CH), 123.26 (CH=CH), 131.15 (CH=CH), 133.42 (2×C), 134.55 (C-O), 142.61 (CH-N), 149.38 (C-O), 149.58 (C-O), 152.09 (CH-N), 153.64 (2×C-O) ppm. HRMS (EI): Found 412.1830 [M+H]^+^; C_22_H_26_N_3_O_5_ requires 412.1872.

**(*E*)-1-(3-(4-Fluorophenyl)-1-(3,4,5-trimethoxyphenyl)allyl)-1*H*-1,2,4-triazole (22e):** As per general method II (*E*)-3-(4-fluorophenyl)-1-(3,4,5-trimethoxyphenyl)prop-2-en-1-ol (**21f**) (1 eq, 1.44 mmol, 0.46 g) was reacted with 1,2,4-triazole and *p*-TSA in toluene. The crude product was purified via flash chromatography (eluent: ethyl acetate/*n*-hexane 7:3) over silica gel to afford the desired product as a yellow oil. Yield: 68% (0.36 g). IR: ν_max_ (ATR) cm^−1^: 2937, 2836, 1703, 1599, 1467, 1417, 1309, 1124, 1094, 1040, 958, 922, 842, 805, 763, 699. ^1^H NMR (400 MHz, CDCl_3_) δ 3.83 (s, 3 H, OCH_3_), 3.84 (s, 6 H, 2×OCH_3_), 6.16 (d, *J* = 6.6 Hz, 1 H, CH-N-R), 6.34–6.40 (m, 1 H, CH=CH), 6.47 (s, 2 H, Ar-H), 6.54 (d, *J* = 6.2 Hz, 1 H, CH=CH), 7.06–7.11 (m, 2 H, Ar-H), 7.34–7.38 (m, 2 H, Ar-H), 8.02 (s, 1 H, CH-N), 8.13 (s, 1 H, CH-N). ^13^C NMR (101 MHz, CDCl_3_) 56.15 (2×OCH_3_), 66.14 (OCH_3_), 60.87 (CH-N-R), 104.52 (2×CH), 115.55 (CH), 115.93 (CH), 124.75 (CH=CH), 128.45 (2×CH), 130.92 (CH=CH), 133.07 (2×C), 138.60 (C-O), 142.62 (CH-N), 152.05 (CH-N), 153.65 (2×C-O), 164.01 (C-F) ppm. HRMS (EI): Found 368.1419 [M-H]^+^; C_20_H_19_FN_3_O_3_ requires 368.1411.

**(*E*)-1-(3-Phenyl-1-(3,4,5-trimethoxyphenyl)allyl)-1*H*-1,2,4-triazole (22f):** As per general method II (*E*)-3-phenyl-1-(3,4,5-trimethoxyphenyl)prop-2-en-1-ol (**21g**) (1 eq, 1.45 mmol, 0.44 g) was reacted with 1,2,4-triazole and *p*-TSA in toluene. The crude product was purified via flash chromatography (eluent: ethyl acetate/*n*-hexane 9:1) over silica gel to afford the desired product as a yellow oil. Yield: 76%, 0.39 g,. IR: ν_max_ (ATR) cm^−1^: 3116, 3061, 2938, 2838, 1583, 1502, 1453, 1418, 1329, 1273, 1238, 1122, 1003, 755, 677, 600, 556. ^1^H NMR (400 MHz, CDCl_3_) δ 3.84 (s, 3 H, OCH_3_), 3.86 (s, 6 H, 2×OCH_3_), 6.2 (d, *J* = 7.1 Hz, 1 H, CH-N-R), 6.38–6.43 (m, 1 H, CH=CH), 6.48 (s, 2 H, Ar-H), 6.62–6.68 (m, 1 H, CH=CH), 7.39–7.43 (m, 5 H, Ar-H), 8.13 (s, 1 H, CH-N), 8.15 (s, 1 H, CH-N). ^13^C NMR (101 MHz, CDCl_3_) 56.19 (2×OCH_3_), 60.91 (OCH_3_), 66.24 (CH-N-R), 104.54 (2×CH), 125.07 (CH=CH), 127.49 (2×CH), 128.71 (CH), 129.11 (2×CH), 131.12 (CH=CH), 133.23 (C), 135.43 (C), 138.23 (C-O), 142.67 (CH), 152.17 (CH), 153.37 (2×C-O) ppm. HRMS (EI): Found 368.1610 [M+OH]^+^; C_20_H_22_N_3_O_4_ requires 368.1610.

**(*E*)-1-(3-(4-Nitrophenyl)-1-(3,4,5-trimethoxyphenyl)allyl)-1*H*-1,2,4-triazole (22g):** As per general method II (*E*)-3-(4-nitrophenyl)-1-(3,4,5-trimethoxyphenyl)prop-2-en-1-ol (**21 h**) (1 eq, 1.16 mmol, 0.40 g) was reacted with 1,2,4-triazole and *p*-TSA in toluene. The crude product was purified via flash chromatography (eluent: ethyl acetate/*n*-hexane 9:1) over silica gel to afford the desired product as a yellow oil. Yield: 50%, 0.22 g,. IR: ν_max_ (ATR) cm^−1^: 3447, 3110, 3003, 2938, 2838, 1591, 1582, 1503, 1417, 1325, 1274, 1243, 1120, 995, 973, 826, 740, 728, 618. ^1^H NMR (400 MHz, CDCl_3_) δ 3.83 (s, 6 H, 2×OCH_3_), 3.85 (s, 3 H, OCH_3_), 6.13 (d, *J* = 5.8 Hz, 1 H, CH-N-R), 6.53 (s, 2 H, Ar-H), 6.54 (d, *J* = 16.0 Hz, 1 H, CH=CH), 6.85 (dd, *J* = 15.8, 6.6 Hz, 1 H, CH=CH), 7.52–7.56 (m, 2 H, Ar-H), 8.05 (s, 1 H, CH-N), 8.13 (s, 1 H, CH-N), 8.17–8.20 (m, 2 H, Ar-H). ^13^C NMR (101 MHz, CDCl_3_) 56.24 (2×OCH_3_), 60.84 (OCH_3_), 65.82 (CH-N-R), 104.78 (2×CH), 124.04 (2×CH), 127.41 (2×CH), 130.44 (CH=CH), 131.97 (CH=CH), 132.21 (C), 136.26 (C-O), 141.81 (C), 142.81 (CH-N), 147.48 (C-NO_2_), 152.33 (CH-N), 153.83 (2×C-O) ppm. HRMS (EI): Found 395.1359 [M-H]^+^; C_20_H_19_N_4_O_5_ requires 395.1356.

#### 4.1.3. General Method III: Preparation of Series 2 (E)-1-(1,3-Diarylallyl)-1*H*-Imidazoles (**23a–e**)

CDI (1,1′-Carbonyldiimidazole) (1.3 eq) was added to a solution of the appropriate (*E*)-1,3-diarylprop-2-en-1-ol (1 eq) in dry acetonitrile (60 mL). The reaction mixture was heated at reflux for 3 h under nitrogen. The solvent was evaporated, and the crude product was dissolved in DCM (30 mL) and washed with water (20 mL) and brine (10 mL). The product was dried over anhydrous sodium sulfate and concentrated under reduced pressure, and the crude product was purified by flash chromatography over silica gel to give the desired product.

**(*E*)-1-(3-(4-Methoxyphenyl)-1-(3,4,5-trimethoxyphenyl)allyl)-1*H*-imidazole (23a)**. As per general method III, (*E*)-3-(4-methoxyphenyl)-1-(3,4,5-trimethoxyphenyl)prop-2-en-1-ol (**21a)** (1 eq, 1.5 mmol, 0.5 g) was reacted with CDI in dry ACN at reflux for 3 h under nitrogen. The crude product was then purified via flash chromatography (ethyl acetate /*n*-hexane/ methanol: 10:1:2) to afford the desired product as a brown oil. Yield: 38%, 0.218 g,. IR: ν_max_ (ATR) cm^−1^: 2999, 2936, 2837, 1583, 1508, 1459, 1417, 1328, 1243, 1176, 1122, 1028, 972, 821, 774, 733. ^1^H NMR (400 MHz, CDCl_3_) δ 7.58 (s, 1 H, CH-N), 7.32 (d, *J* = 8.7 Hz, 2 H, Ar-H), 7.17 (d, *J* = 8.7 Hz, 2 H, Ar-H), 7.10 (s, 1 H, CH-N), 6.57 (s, 2 H, Ar-H), 6.43–6.37 (m, 3 H, Ar-H, CH=CH), 6.28 (d, *J* = 15.7 Hz, 1 H, CH=CH), 5.88 (d, *J* = 6.4 Hz, 1 H, CH-N-R), 3.84 (s, 6 H, 2×OCH_3_), 3.83 (s, 3 H, OCH_3_), 3.81 (s, 3 H, OCH_3_). ^13^C NMR (101 MHz, CDCl_3_) 159.76 (C-O), 153.39 (2×C-O), 138.46 (CH), 136.49 (C-O), 133.56 (C), 131.78 (CH=CH), 130.18 (2×CH), 128.87 (C), 126.44 (CH-N), 124.03 (CH=CH), 118.62 (CH-N), 114.41 (2×CH), 106.86 (2×CH), 63.67 (CH-N-R), 60.92 (OCH_3_), 56.13 (2×OCH_3_), 55.34 (OCH_3_) ppm. HRMS (EI): Found 415.1421 [M+Cl]^+^; C_22_H_24_^35^ClN_2_O_4_ requires 415.1425.

**(*E*)-5-(3-(1*H*-Imidazol-1-yl)-3-(3,4,5-trimethoxyphenyl)prop-1-en-1-yl)-2-methoxyphenol (23b).** As per general method III, (*E*)-5-(3-hydroxy-3-(3,4,5-trimethoxyphenyl)prop-1-en-1-yl)-2-methoxyphenol (**21b**) (1 eq, 1.7 mmol, 0.6 g) was reacted with CDI in ACN at reflux for 3 h under nitrogen. The crude product was then purified via flash chromatography (eluent: ethyl acetate/*n*-hexane/methanol: 1:1:1) to afford the desired product as a brown oil. Yield: 26% (0.176 g). IR: ν_max_ (ATR) cm^−1^: 3118, 2937, 2837, 1582, 1505, 1453, 1417, 1328, 1274, 1236, 1077, 1024, 968, 803, 731, 659. ^1^H NMR (400 MHz, CDCl_3_) δ 7.62 (s, 1 H, CH-N), 6.94 (s, 1 H, CH-N), 6.73 (dd, *J* = 8.4, 2.0 Hz, 1 H, Ar-H), 6.57 (s, 2 H, Ar-H), 6.47–6.43 (m, 1 H, CH=CH), 6.40 (s, 2 H, Ar-H), 6.36 (m, 1 H, CH=CH), 5.83 (d, *J* = 6.4 Hz, 1 H, CH-N-R), 3.89 (s, 3 H, OCH_3_), 3.85 (s, 6 H, 2×OCH_3_), 3.83 (s, 3 H, OCH_3_). ^13^C NMR (101 MHz, CDCl_3_) 153.67 (2×C-O), 147.17 (2×C-O), 134.00 (C), 133.85 (C), 131.10 (CH=CH), 128.54 (CH), 123.11 (CH=CH), 120.95 (CH), 119.18 (CH), 113.83 (CH), 110.93 (CH), 103.84 (2×CH), 62.98 (CH), 60.92 (OCH_3_), 56.20 (OCH_3_), 56.14 (2×OCH_3_) ppm. HRMS (EI): Found 395.1613 [M-H]^+^; C_22_H_23_N_2_O_5_ requires 395.1607.

**(*E*)-1-(1,3-Bis(3,4,5-trimethoxyphenyl)allyl)-1*H*-imidazole (23c):** As per general method III, (*E*)-1,3-bis(3,4,5-trimethoxyphenyl)prop-2-en-1-ol (**21c**) (1 eq, 0.97 mmol, 0.379 g) was reacted with CDI in dry ACN at reflux for 3 h under nitrogen. The crude product was then purified via flash chromatography (eluent: ethyl acetate/*n*-hexane: 9:1) to afford the desired product as a brown oil. Yield: 30%, 0.126 g. IR: ν_max_ (ATR) cm^−1^: 2936, 2838, 1583, 1504, 1459, 1418, 1328, 1237, 1121, 1001, 823, 779, 727, 691, 662. ^1^H NMR (400 MHz, CDCl_3_) δ 3.82 (s, 6 H, 2×OCH_3_), 3.86 (s, 3 H, OCH_3_), 3.87 (s, 3 H, OCH_3_), 3.88 (s, 6 H, 2×OCH_3_), 5.87 (d, *J* = 6.2 Hz, 1 H, CH-N-R), 6.33–6.38 (m, 1 H, CH=CH), 6.44 (s, 3 H, Ar-H, CH=CH), 6.61 (s, 2 H, Ar-H), 6.98 (br. s., 1 H, CH-N), 7.16 (br. s., 1 H, CH-N), 7.67 (br. s., 1 H, CH-N). ^13^C NMR (101 MHz, CDCl_3_) 56.14 (2×OCH_3_), 56.21 (2×OCH_3_), 60.84 (OCH_3_), 60.89 (OCH_3_), 63.37 (CH-N-R), 103.90 (2×CH), 104.57 (2×CH), 118.65 (CH-N), 120.92 (CH=CH), 125.78 (CH-N), 131.07 (CH=CH), 133.87 (C), 134.13 (C), 136.56 (C), 138.13 (CH-N), 138.58 (C-O), 153.40 (2×C-O), 153.67 (2×C-O) ppm. HRMS (EI): Found 441.2006 [M+H]^+^; C_24_H_29_N_2_O_6_ requires 441.2025.

**(*E*)-1-(3-(3,4-Dimethoxyphenyl)-1-(3,4,5-trimethoxyphenyl)allyl)-1*H*-imidazole (23d):** As per general method III, (*E*)-3-(3,4-dimethoxyphenyl)-1-(3,4,5-trimethoxyphenyl)prop-2-en-1-ol (**21d**) (1 eq, 1.1 mmol, 0.44 g) was reacted with CDI in dry ACN (50 mL) at reflux for 3 h under nitrogen. The crude product was then purified via flash chromatography (eluent: ethyl acetate/*n*-hexane/methanol: 9:1:1) to afford the desired product as a dark brown oil. Yield: 45%, 0.20 g. IR: ν_max_ (ATR) cm^−1^: 3117, 2999, 2937, 2836, 1584, 1507, 1262, 1232, 1185, 1022, 971, 920, 855, 810, 764, 740. ^1^H NMR (400 MHz, CDCl_3_) δ 3.82 (s, 3 H, OCH_3_), 3.85 (s, 3 H, OCH_3_), 3.87 (s, 6 H, 2×OCH_3_), 3.90 (s, 3 H, OCH_3_), 5.90 (d, *J* = 6.2 Hz, 1 H, CH-N-R), 6.28–6.33 (m, 1 H, CH=CH), 6.40 (d, *J* = 2.9 Hz, 1 H, Ar-H), 6.43 (s, 1 H, CH=CH), 6.44–6.50 (m, 1 H, Ar-H), 6.60 (s, 2 H, Ar-H), 6.73 (d, *J* = 2.1 Hz, 1 H, Ar-H), 6.88 (s, 1 H, CH-N), 7.12–7.14 (m, 1 H, CH-N), 7.59 (s, 1 H, CH-N). ^13^C NMR (101 MHz, CDCl_3_) 153.41 (2×C-O), 149.43 (C-O), 149.31 (C-O), 136.56 (C-O, CH-N), 133.73 (2×C), 131.19 (CH=CH), 128.82 (CH-N), 124.30 (CH=CH), 120.00 (CH-N), 118.66 (CH), 111.28 (CH), 110.63 (CH), 109.16 (2×CH), 62.97 (CH-N-R), 60.92 (OCH_3_), 56.22 (OCH_3_), 56.15 (2×OCH_3_), 55.96 (OCH_3_) ppm. HRMS (EI): Found 409.1769 [M-H]^+^; C_23_H_25_N_2_O_5_ requires 409.1764.

**(*E*)-1-(3-(4-Fluorophenyl)-1-(3,4,5-trimethoxyphenyl)allyl)-1*H*-imidazole (23e):** As per general method III, (*E*)-3-(4-fluorophenyl)-1-(3,4,5-trimethoxyphenyl)prop-2-en-1-ol (**21f**) (1 eq, 1.25 mmol, 0.40 g) was reacted with CDI in dry ACN at reflux for 3 h under nitrogen. The crude product was then purified via flash chromatography (eluent: ethyl acetate/*n*-hexane: 9:1) to afford the desired product as a brown oil. Yield: 45%, 0.20 g. IR: ν_max_ (ATR) cm^−1^: 2940, 2838, 1590, 1506, 1459, 1418, 1327, 1223, 1157, 1122, 1001, 803, 777, 733. ^1^H NMR (400 MHz, CDCl_3_) δ 3.82 (s, 6 H, 2×OCH_3_), 3.87 (s, 3 H, OCH_3_), 5.85 (d, *J* = 5.0 Hz, 1 H, CH-N-R), 6.32 (d, *J* = 16.0 Hz, 1 H, CH=CH), 6.43 (s, 2 H, Ar-H), 6.97 (br. s., 1 H, Ar-H), 7.02–7.06 (m, 2 H, Ar-H), 7.14 (br. s., 1 H, Ar-H), 7.21–7.26 (m, 1 H, CH=CH), 7.36–7.40 (m, 2 H, Ar-H), 7.62 (br. s., 1 H, Ar-H). ^13^C NMR (101 MHz, CDCl_3_) 56.22 (2×OCH_3_), 60.88 (OCH_3_), 77.20 (CH-N-R), 104.51 (2×CH), 115.65 (2×CH), 116.18 (CH-N), 126.26 (CH-N), 128.35 (2×CH), 128.85 (CH=CH), 129.23 (2×C), 133.02 (C-O), 134.20 (CH-N), 153.71 (2×C-O), 159.79 (C-F) ppm. HRMS (EI): Found 367.1460 [M-H]^+^; C_21_H_20_FN_2_O_3_ requires 367.1458.

**(*E*)-1-(3-(4-Chlorophenyl)-1-(3,4,5-trimethoxyphenyl)allyl)-1*H*-imidazole 23f:** General method III was followed using (*E*)-3-(4-chlorophenyl)-1-(3,4,5-trimethoxyphenyl)prop-2-en-1-ol **21j** (1 equiv; 1.19 mmol, 400 mg) and stirred for 3 h, before purification using n-hexane:AcOEt:MeOH (7:3:1 gradient) to afford the pure product as a brown oil (27%). ^1^H NMR (400 MHz, CDCl_3_): δ 7.53 (s, 1 H), 7.32 (d, *J* = 8.4 Hz, 1 H), 7.26 (d, *J* = 3.1 Hz, 3 H), 7.12 (d, *J* = 8.4 Hz, 1 H), 7.06 (s, 1 H), 6.91 (s, 1 H), 6.55 (s, 1 H), 6.46 (d, *J* = 6.6 Hz, 1 H), 6.38 (s, 2 H), 5.81 (d, *J* = 6.6 Hz, 1 H), 3.80 (s, 3 H, OCH_3_), 3.76 (s, 6 H, 2×OCH_3_). ^13^C NMR (101 MHz, CDCl_3_): 153.81, 134.59, 134.33, 134.15, 133.99, 132.96, 129.36, 129.03, 128.08, 127.34, 125.69, 104.65, 104.02, 63.40, 60.97, 56.32 ppm.

#### 4.1.4. General Method IV: Preparation of Indanones (**24a–i**)

The appropriate chalcone (**20b–j**) (1 eq) was reacted with an excess of trifluoroacetic acid (TFA) in a microwave tube for 10 min at 120 °C. Once the reaction was complete, the reaction mixture was dissolved in ethyl acetate (20 mL), extracted with sodium bicarbonate (10%, 10 mL), washed with water (10 mL) and brine (5 mL), and dried over sodium sulfate. The solution was filtered and concentrated using a rotary evaporator. The crude product was then purified by flash column chromatography over silica gel.

**3-(3-Hydroxy-4-methoxyphenyl)-4,5,6-trimethoxy-2,3-dihydro-1*H*-inden-1-one (24a):** As per general method IV, (*E*)-3-(3-hydroxy-4-methoxyphenyl)-1-(3,4,5-trimethoxyphenyl)prop-2-en-1-one (**20b**) (1 eq, 1.76 mmol, 0.61 g) was reacted with TFA (3 mL). The crude product was purified via flash column chromatography (eluent: *n*-hexane/ethyl acetate 3:7) to afford the pure product as a white solid. Yield: 68%, 0.41 g, Mp. 122–124 °C [85]. IR: ν_max_ (ATR) cm^−1^: 3240, 2957, 2937, 2835, 1691, 1586, 1509, 1462, 1319, 1271, 1210, 1099, 1025, 1005, 955, 844, 807, 660, 591. ^1^H NMR (400 MHz, DMSO-*d*_6_) δ 2.29 (dd, *J* = 19.1, 2.49 Hz, 1 H, CH_2_), 3.12 (dd, *J* = 19.1, 7.9 Hz, 1 H, CH_2_), 3.36 (s, 3 H, OCH_3_), 3.67 (s, 3 H, OCH_3_), 3.76 (s, 3 H, OCH_3_), 3.84 (s, 3 H, OCH_3_), 4.46 (dd, *J* = 7.9, 2.1 Hz, 1 H, CH), 6.37 (d, *J* = 2.1 Hz, 1 H, Ar-H), 6.46 (dd, *J* = 8.3, 2.1 Hz, 1 H, Ar-H), 6.77 (d, *J* = 8.3 Hz, 1 H, Ar-H), 7.01 (s, 1 H, Ar-H), 8.82 (s, 1 H, OH). ^13^C NMR (101 MHz, DMSO-*d*_6_) 40.22 (CH), 47.04 (CH_2_), 55.59 (OCH_3_), 56.10 (OCH_3_), 59.82 (OCH_3_), 60.51 (OCH_3_), 100.17 (CH), 112.25 (CH), 113.98 (CH), 117.66 (CH), 131.69 (C), 137.03 (C), 146.16 (C), 146.50 (2×C), 148.02 (C), 149.94 (C), 154.41 (C), 204.24 (C=O) ppm. LRMS (EI): Found 343.23 [M-H]^+^; C_19_H_19_O_6_ requires 343.12.

**4,5,6-Trimethoxy-3-(3,4,5-trimethoxyphenyl)-2,3-dihydro-1*H*-inden-1-one (24b)**: As per general method IV, (*E*)-1,3-bis(3,4,5-trimethoxyphenyl)prop-2-en-1-one (**20c**) (1eq, 1.54 mmol, 0.6 g) was reacted with trifluoroacetic acid (2 mL) in a sealed tube at 120 °C. On completion, the contents of the tube were poured into cold water and extracted with ethyl acetate (30 mL). The crude indanone product was then purified via flash column chromatography (eluent: *n*-hexane/ethyl acetate 3:7) to afford the desired product as a brown oil. Yield: 76%, 0.46 g, brown oil [126]. IR: ν_max_ (ATR) cm^−1^: 3301, 2938, 2838, 1703, 1588, 1460, 1415, 1329, 1312, 1218, 1158, 1120, 1095, 1001, 955, 923, 846, 779. ^1^H NMR (400 MHz, CDCl_3_) δ 2.63 (dd, *J* = 19.1, 2.5 Hz, 1 H, CH_2_), 3.19 (dd, *J* = 19.3, 8.1 Hz, 1 H, CH_2_), 3.43 (s, 3 H, OCH_3_), 3.78 (s, 6 H, 2×OCH_3_), 3.81 (s, 3 H, OCH_3_), 3.92 (s, 3 H, OCH_3_), 3.93 (s, 3 H, OCH_3_), 4.52 (dd, *J* = 8.3, 2.5 Hz, 1 H, CH), 6.30 (s, 2 H, Ar-H), 7.10 (s, 1 H, Ar-H). ^13^C NMR (101 MHz, CDCl_3_) 41.96 (C), 47.04 (CH_2_), 56.12 (2×OCH_3_), 56.21 (OCH_3_), 60.15 (OCH_3_), 60.88 (2×OCH_3_), 100.39 (CH), 104.21 (2×CH), 132.03 (C), 136.64 (C-O), 140.00 (C), 144.42 (C), 148.92 (C-O), 150.34 (C-O), 153.28 (2×C-O), 154.96 (C-O), 205.78 (C=O) ppm. HRMS (EI): Found 389.1595 [M+H]^+^; C_21_H_25_O_7_ requires 389.1600.

**3-(3,4-Dimethoxyphenyl)-4,5,6-trimethoxy-2,3-dihydro-1*H*-inden-1-one (24c):** As per general method IV, (*E*)-3-(3,4-dimethoxyphenyl)-1-(3,4,5-trimethoxyphenyl)prop-2-en-1-one (**20d**) (1 eq, 3.34 mmol, 1.2 g) was reacted with TFA (6 mL). The crude product was purified via flash column chromatography (eluent: *n*-hexane/ethyl acetate 3:7) to afford the desired product as a brown oil. Yield: 79%, 0.95 g, brown oil. IR: ν_max_ (ATR) cm^−1^: 2973, 2938, 2842, 1747, 1588, 1505, 1449, 1278, 1226, 1123, 1021, 999, 985, 831, 808, 734, 704, 629. ^1^H NMR (400 MHz, CDCl_3_) δ 2.60 (dd, *J* = 19.1, 2.5 Hz, 1 H, CH_2_), 3.17 (dd, *J* = 19.1, 8.3 Hz, 1 H, CH_2_), 3.38 (s, 3 H, OCH_3_), 3.80 (s, 3 H, OCH_3_) 3.84 (s, 3 H, OCH_3_), 3.90 (s, 3 H, OCH_3_), 3.92 (s, 3 H, OCH_3_), 4.53 (dd, *J* = 7.8, 2.9 Hz, 1 H, CH), 6.60–6.66 (m, 2 H, Ar-H), 6.74–6.81 (m, 1 H, Ar-H), 7.08 (s, 1 H, Ar-H). ^13^C NMR (101 MHz, CDCl_3_) 41.35 (C), 47.26 (CH_2_), 55.88 (OCH_3_), 55.92 (OCH_3_), 56.24 (OCH_3_), 60.18 (OCH_3_), 60.91 (OCH_3_), 100.40 (CH), 110.49 (CH), 111.27 (CH), 119.23 (CH), 131.95 (C), 136.76 (C), 147.72 (2×C-O), 149.05 (2×C-O), 154.91 (C-O), 206.25 (C=O) ppm. HRMS (EI): Found 359.1500 [M+H]^+^; C_20_H_23_N_2_O_6_ requires 359.1494.

**3-(4-Ethoxyphenyl)-4,5,6-trimethoxy-2,3-dihydro-1*H*-inden-1-one (24d):** As per general method IV, (*E*)-3-(4-ethoxyphenyl)-1-(3,4,5-trimethoxyphenyl)prop-2-en-1-one (**20e**) (1 eq, 1.75 mmol, 0.6 g) was reacted with TFA (3 mL). The crude product was purified via flash column chromatography (eluent: *n*-hexane/ethyl acetate 3:7) to afford the desired product as a pale yellow solid. Yield: 44%, 0.26 g, Mp. 82–85 °C. IR: ν_max_ (ATR) cm^−1^: 2977, 2937, 2901, 1702, 1599, 1468, 1339, 1308, 1240, 1125, 1093, 1043, 922, 835, 727, 623, 597. ^1^H NMR (400 MHz, CDCl_3_) δ 1.37 (t, *J* = 7.1 Hz, 3 H, CH_3_), 2.56 (dd, *J* = 19.1, 2.5 Hz, 1 H, CH_2_), 3.15 (dd, *J* = 19.3, 8.1 Hz, 1 H, CH_2_), 3.34 (s, 3 H, OCH_3_), 3.88 (s, 3 H, OCH_3_), 3.90 (s, 3 H, OCH_3_), 3.97 (q, *J* = 7.1 Hz, 2 H, CH_2_), 4.52 (dd, *J* = 7.9, 2.5 Hz, 1 H, CH), 6.77–6.82 (m, 2 H, Ar-H) 6.96–7.02 (m, 2 H, Ar-H), 7.06 (s, 1 H, Ar-H). ^13^C NMR (101 MHz, CDCl_3_) 14.77 (CH_3_), 40.79 (CH), 47.27 (CH_2_), 56.16 (OCH_3_), 60.02 (OCH_3_), 60.80 (OCH_3_), 63.36 (CH_2_), 100.18 (CH), 114.48 (2×CH), 128.13 (2×CH), 132.06 (C), 136.22 (C), 144.87 (C), 148.76 (C-O), 150.34 (C-O), 154.76 (C-O), 157.57 (C-OEt), 205.46 (C=O) ppm. HRMS (EI): Found 365.1350 [M+Na]^+^; C_20_H_22_NaO_5_ requires 342.1467.

**3-(4-Fluorophenyl)-4,5,6-trimethoxy-2,3-dihydro-1*H*-inden-1-one (24e):** As per general method IV, (*E*)-3-(4-fluorophenyl)-1-(3,4,5-trimethoxyphenyl)prop-2-en-1-one (**20f**) (1 eq, 1.26 mmol, 0.4 g) was reacted with TFA (1.5 mL). The crude product was purified via flash column chromatography (eluent: *n*-hexane/ethyl acetate 3:7) to afford the desired product as a yellow oil. Yield: 96%, 0.38 g. IR: ν_max_ (ATR) cm^−1^: 2937, 1702, 1507, 1466, 1220, 1122, 1093, 1041, 1025, 1000, 961, 922, 834, 734. ^1^H NMR (400 MHz, CDCl_3_) δ 2.56 (dd, *J* = 19.1, 2.1 Hz, 1 H, CH_2_), 3.14–3.22 (m, 1 H, CH_2_), 3.38 (s, 3 H, OCH_3_), 3.90 (s, 3 H, OCH_3_), 3.92 (s, 3 H, OCH_3_), 4.57 (dd, *J* = 7.9, 2.5 Hz, 1 H, CH), 6.94–6.99 (m, 2 H, Ar-H), 7.04–7.08 (m, 2 H, Ar-H), 7.09 (s, 1 H, Ar-H). ^13^C NMR (101 MHz, CDCl_3_) 40.81 (CH), 47.11 (CH_2_), 56.21 (OCH_3_), 60.00 (OCH_3_), 60.84 (OCH_3_), 100.26 (CH), 115.27 (CH), 115.49 (CH), 128.60 (2×CH), 128.68 (CH), 132.09 (C), 140.08 (C), 150.27 (2×C-O), 155.00 (C-O), 162.73 (C-F), 204.93 (C=O) ppm. HRMS (EI): Found 339.1010 [M+Na]^+^; C_18_H_17_FNaO_4_ requires 339.1009.

**4,5,6-Trimethoxy-3-phenyl-2,3-dihydro-1*H*-inden-1-one (24f):** As per general method IV, (*E*)-3-phenyl-1-(3,4,5-trimethoxyphenyl)prop-2-en-1-one **(20g**) (1 eq, 1.34 mmol, 0.61 g) was reacted with TFA (3 mL). No further purification was required and afforded the desired product as a yellow oil. Yield: 65%, 0.39 g [80]. IR: ν_max_ (ATR) cm^−1^: 2937, 2836, 1703, 1599, 1467, 1417, 1330, 1309, 1124, 1094, 1040, 922, 842, 763, 699. ^1^H NMR (400 MHz, CDCl_3_) δ 2.62 (dd, *J* = 19.5, 2.5 Hz, 1 H, CH_2_), 3.19 (dd, *J* = 19.5, 7.9 Hz, 1 H, CH_2_), 3.31 (s, 3 H, OCH_3_), 3.90 (s, 3 H, OCH_3_), 3.89 (s, 3 H, OCH_3_), 4.56 (dd, *J* = 7.9, 2.5 Hz, 1 H, CH), 7.07–7.09 (m, 2 H, Ar-H), 7.10 (d, *J* = 1.2 Hz, 1 H, Ar-H), 7.16–7.20 (m, 1 H, Ar-H), 7.24–7.28 (m, 2 H, Ar-H). ^13^C NMR (101 MHz, CDCl_3_) 41.59 (CH), 47.05 (CH_2_), 56.13 (OCH_3_), 59.88 (OCH_3_), 60.76 (OCH_3_), 100.29 (C), 126.58 (C), 127.16 (2×CH), 128.52 (2×CH), 131.93 (C), 148.90 (C), 150.24 (2×C), 154.85 (C), 205.98 (C=O) ppm. HRMS (EI): Found 299.1269 [M+H]^+^; C_18_H_19_O_4_ requires 299.1283.

**4,5,6-Trimethoxy-3-(4-nitrophenyl)-2,3-dihydro-1*H*-inden-1-one (24g):** As per general method IV, (*E*)-3-(4-nitrophenyl)-1-(3,4,5-trimethoxyphenyl)prop-2-en-1-one (**20h**) (1 eq, 1.17 mmol, 0.405 g) was reacted with TFA (2.5 mL). The crude product was purified via flash column chromatography (*n*-hexane:ethyl acetate 3:7) to afford the desired product as a yellow solid. Yield: 60% (0.24 g) Mp. 104–107 °C, HPLC: 88%. IR: ν_max_ (ATR) cm^−1^: 3488, 2995, 2936, 1701, 1595, 1510, 1466, 1431, 1336, 1308, 1115, 1102, 1025, 860, 754, 703, 647, 630, 562, 576. ^1^H NMR (400 MHz, CDCl_3_) δ 2.55 (dd, *J* = 19.3, 2.7 Hz, 1 H, CH_2_), 3.21 (dd, *J* = 19.3, 8.1 Hz, 1 H, CH_2_), 3.43 (s, 3 H, OCH_3_), 3.87 (s, 3 H, OCH_3_), 3.91 (s, 3 H, OCH_3_), 4.66 (dd, *J* = 7.9, 2.5 Hz, 1 H, CH), 7.08 (s, 1 H, Ar-H), 7.24–7.28 (m, 2 H, Ar-H), 8.14 (d, *J* = 8.7 Hz, 2 H, Ar-H). ^13^C NMR (101 MHz, CDCl_3_) 41.30 (CH), 46.54 (CH_2_), 56.29 (OCH_3_), 60.07 (OCH_3_), 60.94 (OCH_3_), 100.42 (CH), 123.93 (2×CH), 125.38 (C), 128.10 (2×CH), 132.16 (C), 142.73 (C), 148.58 (C), 150.07 (2×C-O), 152.03 (C-O), 203.87 (C=O) ppm. HRMS (EI): Found 344.1140 [M+H]^+^; C_18_H_18_NO_6_ requires 344.1135.

**4,5,6-Trimethoxy-3-(4-methoxy-3-nitrophenyl)-2,3-dihydro-1*H*-inden-1-one (24h):** As per general method IV, (*E*)-3-(4-methoxy-3-nitrophenyl)-1-(3,4,5-trimethoxyphenyl)prop-2-en-1-one (**20i**) (1 eq, 2.84 mmol, 1.06 g) was reacted with TFA (6.5 mL). The crude product was purified via flash column chromatography (eluent: *n*-hexane/ethyl acetate 3:7) to afford the desired product as a brown solid. Yield: 94%, 2.7 g, Mp. 139–142 °C [85]. IR: ν_max_ (ATR) cm^−1^: 2941, 2836, 1696, 1623, 1597, 1524, 1460, 1421, 1343, 1311, 1251, 1128, 1093, 1026, 975, 825, 783, 711, 688. ^1^H NMR (400 MHz, CDCl_3_) δ 2.53–2.59 (m, 1 H, CH_2_), 3.21 (dd, *J* = 19.3, 8.1 Hz, 1 H, CH_2_), 3.52 (s, 3 H, OCH_3_), 3.91 (s, 3 H, OCH_3_), 3.93 (s, 3 H, OCH_3_), 3.94 (s, 3 H, OCH_3_), 4.59 (dd, *J* = 8.1, 2.7 Hz, 1 H, CH), 7.02 (d, *J* = 8.3 Hz, 1 H, Ar-H), 7.10 (s, 1 H, Ar-H), 7.25 (d, *J* = 2.1 Hz, 1 H, Ar-H), 7.65 (d, *J* = 2.5 Hz, 1 H, Ar-H). ^13^C NMR (101 MHz, CDCl_3_) 40.23 (CH), 46.59 (CH_2_), 56.19 (OCH_3_), 56.49 (OCH_3_), 60.10 (OCH_3_), 60.85 (OCH_3_), 100.32 (CH), 113.72 (CH), 124.32 (C, CH), 136.64 (C, CH), 142.79 (C), 150.07 (2×C), 151.53 (C), 155.25 (C), 204.06 (C=O) ppm. HRMS (EI): Found 396.1043 [M+Na]^+^; C_19_H_19_NNaO_7_ requires 396.1059.

**3-(4-Hydroxyphenyl)-4,5,6-trimethoxy-2,3-dihydro-1*H*-inden-1-one (24i):** As per general method IV, (*E*)-3-(4-hydroxyphenyl)-1-(3,4,5-trimethoxyphenyl)prop-2-en-1-one (**20j**) (1 eq, 0.95 mmol, 0.3 g) was reacted with TFA (2.5 mL). The crude product was purified via flash column chromatography (*n*-hexane: ethyl acetate 1:1) to afford the desired product as a white solid. Yield: 50% (0.15 g), Mp.: 137–139 °C. IR: ν_max_ (ATR) cm^−1^: 3255, 2988, 2942, 1703, 1676, 1596, 1586, 1463, 1450, 1343, 1327, 1222, 1126, 1096, 922, 827, 675, 652, 604. ^1^H NMR (400 MHz, CDCl_3_) δ 7.06 (s, 1 H, Ar-H), 6.95–6.91 (m, 2 H, Ar-H), 6.75–6.71 (m, 2 H, Ar-H), 4.51 (dd, *J* = 7.9, 2.4 Hz, 1 H, CH), 3.88 (s, 6 H, 2×OCH_3_), 3.34 (s, 3 H, OCH_3_), 3.15 (dd, *J* = 19.3, 7.9 Hz, 1 H, CH_2_), 2.55 (dd, *J* = 19.4, 2.4 Hz, 1 H, CH_2_). ^13^C NMR (101 MHz, CDCl_3_) 206.16 (C=O), 154.81 (C-OH), 154.47 (C-O), 150.28 (2×C-O), 145.09 (C), 136.15 (C), 131.94 (C), 128.32 (2×CH), 115.45 (2×CH), 100.32 (CH), 60.86 (OCH_3_), 60.08 (OCH_3_), 56.19 (OCH_3_), 47.31 (CH_2_), 40.84 (CH) ppm. HRMS (APCI): Found 315.1230 [M+H]^+^; C_18_H_19_O_5_ requires 315.1233.

#### 4.1.5. General Method V: Preparation of 3-aryl-4,5,6-Trimethoxy-2,3-Dihydro-1*H*-Inden-1-ols (**25a–i**)

To a solution of the appropriate 3-aryl-4,5,6-trimethoxy-2,3-dihydro-1*H*-inden-1-one (1 eq) in methanol (25 mL), a suspension of sodium borohydride NaBH_4_ (1 eq) in methanol (10 mL) and THF (10 mL) was slowly added. The reaction mixture was stirred (0–20 °C) and monitored by TLC until the reaction was complete. NaHCO_3_ (aqueous saturated solution, 5 mL) was then added, and the reaction mixture was concentrated. The reaction residue was extracted with ethyl acetate, and the organic solution was washed with water and brine and dried over anhydrous sodium sulfate. No further purification was required.

**3-(3-Hydroxy-4-methoxyphenyl)-4,5,6-trimethoxy-2,3-dihydro-1*H*-inden-1-ol (25a):** As per general method V, 3-(3-hydroxy-4-methoxyphenyl)-4,5,6-trimethoxy-2,3-dihydro-1*H*-inden-1-one (**24a**) (1 eq, 1.21 mmol, 0.42 g) was reacted with sodium borohydride (2 eq, 2.42 mmol, 0.10 g) in methanol and THF to afford the desired product as a brown resin. Yield: 86% (0.36 g) [124]. IR: ν_max_ (ATR) cm^−1^: 3405, 2969, 2936, 2988, 1691, 1594, 1465, 1431, 1414, 1507, 1265, 1049, 1113, 1023, 727, 761, 590. ^1^H NMR (400 MHz, CDCl_3_) δ 1.89–1.95 (m, 1 H, CH_2_), 2.95 (ddd, *J* = 13.8, 8.4, 7.3 Hz, 1 H, CH_2_), 3.46 (s, 3 H, OCH_3_), 3.82 (s, 3 H, OCH_3_), 3.86 (s, 3 H, OCH_3_), 3.90 (s, 3 H, OCH_3_), 4.25 (dd, *J* = 8.3, 5.4 Hz, 1 H, CH), 5.14 (dd, *J* = 7.3, 4.77 Hz, 1 H, CH-OH), 5.59 (s, 1 H, OH), 6.70 (d, *J* = 2.1 Hz, 1 H, Ar-H), 6.75 (s, 1 H, Ar-H), 6.80–6.82 (m, 2 H, Ar-H). ^13^C NMR (101 MHz, CDCl_3_) 46.21 (CH_2_), 46.42 (C), 55.93 (OCH_3_), 56.12 (2×OCH_3_), 60.79 (OCH_3_), 75.87 (CH-OH), 102.65 (CH), 110.45 (CH), 113.89 (CH), 118.82 (CH), 139.62 (C), 140.56 (C), 142.61 (C), 144.92 (C-O), 145.44 (2×C-O), 150.11 (C-O) 154.15 (C-O) ppm. HRMS (EI): Found 345.1334 [M-H]^+^; C_19_H_21_O_6_ requires 345.1338.

**4,5,6-Trimethoxy-3-(3,4,5-trimethoxyphenyl)-2,3-dihydro-1*H*-inden-1-ol (25b):** As per general method V, 4,5,6-trimethoxy-3-(3,4,5-trimethoxyphenyl)-2,3-dihydro-1*H*-inden-1-one (**24b**) (1 eq, 1.00 mmol, 0.39 g) was reacted with sodium borohydride (2 eq, 2.00 mmol, 0.07 g) of in methanol (10 mL) and THF (10 mL) to afford the desired product as a dark oil. Yield: 100% (0.39 g) dark oil. IR: ν_max_ (ATR) cm^−1^: 3514, 3207, 2987, 2972, 2941, 1590, 1456, 1414, 1334, 1234, 1112, 1052, 993, 977, 852, 702, 656, 598. ^1^H NMR (400 MHz, CDCl_3_) δ 1.98 (dt, *J* = 13.7, 5.2 Hz, 1 H, CH_2_), 2.98 (ddd, *J* = 13.8, 8.4, 7.3 Hz, 1 H, CH_2_), 3.46 (s, 3 H, OCH_3_), 3.81 (s, 6 H, 2×OCH_3_), 3.82 (s, 3 H, OCH_3_), 3.83 (s, 3 H, OCH_3_), 3.92 (s, 3 H, OCH_3_), 4.26 (dd, *J* = 8.3, 5.4 Hz, 1 H, CH), 5.19 (dd, *J* = 7.1, 4.6 Hz, 1 H, CH-OH), 6.49 (s, 2 H, Ar-H), 6.82 (s, 1 H, Ar-H). ^13^C NMR (101 MHz, CDCl_3_) 46.24 (CH_2_), 47.17 (CH), 56.08 (2×OCH_3_), 56.10 (OCH_3_), 60.13 (OCH_3_), 60.77 (OCH_3_), 60.85 (OCH_3_), 75.74 (CH-OH), 102.60 (CH), 104.68 (2×CH), 130.14 (C), 136.28 (C-O), 140.50 (C), 141.83 (C), 142.58 (C-O), 150.13 (C-O), 153.04 (C-O), 154.27 (2×C-O) ppm. HRMS (EI): Found 413.1578 [M+Na]^+^; C_21_H_26_NaO_7_ requires 413.1576.

**3-(3,4-Dimethoxyphenyl)-4,5,6-trimethoxy-2,3-dihydro-1*H*-inden-1-ol (25c):** As per general method V, 3-(3,4-dimethoxyphenyl)-4,5,6-trimethoxy-2,3-dihydro-1*H*-inden-1-one (**24c**) (1 eq, 2.66 mmol, 0.95 g) was reacted with sodium borohydride (2 eq, 5.33 mmol, 0.20 g) in methanol (20 mL) and THF (20 mL) to afford the desired product as an orange oil. Yield: 78% (0.747 g). IR: ν_max_ (ATR) cm^−1^: 3463, 2997, 2936, 2836, 1512, 1462, 1413, 1334, 1258, 1232, 1113, 1023, 1051, 812, 728, 642, 585, 576.3. ^1^H NMR (400 MHz, CDCl_3_) δ 1.92–1.99 (m, 1 H, CH_2_), 2.97 (ddd, *J* = 13.7, 8.3, 7.5 Hz, 1 H, CH_2_), 3.42 (s, 3 H, OCH_3_), 3.82 (s, 3 H, OCH_3_), 3.83 (s, 3 H, OCH_3_), 3.86 (s, 3 H, OCH_3_), 3.90 (s, 3 H, OCH_3_), 4.28 (dd, *J* = 8.3, 5.8 Hz, 1 H, CH), 5.17 (dd, *J* = 7.1, 5.0 Hz, 1 H, CH-OH), 6.77 (d, *J* = 2.1 Hz, 1 H, Ar-H), 6.78 (s, 1 H, Ar-H), 6.80–6.83 (m, 2 H, Ar-H). ^13^C NMR (101 MHz, CDCl_3_) 46.36 (C), 46.47 (CH_2_), 55.81 (OCH_3_), 55.83 (OCH_3_), 56.08 (OCH_3_), 60.07 (OCH_3_), 60.75 (OCH_3_), 75.75 (CH-OH), 102.60 (CH), 110.93 (CH), 111.05 (CH), 119.41 (C), 130.42 (CH), 138.75 (C), 140.46 (C), 142.58 (C-O), 147.30 (C-O), 148.78 (C-O), 150.12 (C-O), 154.15 (C-O) ppm. HRMS (EI): Found 383.1499 [M+Na]^+^; C_20_H_24_NaO_6_ requires 383.1471.

**3-(4-Ethoxyphenyl)-4,5,6-trimethoxy-2,3-dihydro-1*H*-inden-1-ol (25d):** As per general method V, 3-(4-ethoxyphenyl)-4,5,6-trimethoxy-2,3-dihydro-1*H*-inden-1-one (**24d**) (1 eq, 1.46 mmol, 0.5 g) was reacted with sodium borohydride (2 eq, 2.9 mmol, 0.11 g) in methanol (10 mL) and THF (10 mL) to afford the desired product as a clear oil. Yield: 96% (0.47 g). IR: ν_max_ (ATR) cm^−1^: 3488, 2976, 2919, 2831, 1601, 1583, 1511, 1465, 1428, 1330, 1244, 1231, 1175, 1111, 1088, 1045, 953, 835, 668. ^1^H NMR (400 MHz, CDCl_3_) δ 1.40 (t, *J* = 7.1 Hz, 3 H, CH_3_), 1.93 (dt, *J* = 13.7, 5.2 Hz, 1 H, CH_2_), 2.93–3.02 (m, 1 H, CH_2_), 3.38 (s, 3 H, OCH_3_), 3.82 (s, 3 H, OCH_3_), 3.90 (s, 3 H, OCH_3_), 4.01 (q, *J* = 6.1 Hz, 2 H, CH_2_), 4.28 (dd, *J* = 8.3, 5.4 Hz, 1 H, CH), 5.17 (dd, *J* = 7.1, 5.0 Hz, 1 H, CH-OH), 6.81–6.84 (m, 3 H, Ar-H), 7.12–7.17 (m, 2 H, Ar-H). ^13^C NMR (101 MHz, CDCl_3_) 14.86 (CH_3_) 46.04 (CH_2_), 46.49 (C), 56.11 (OCH_3_), 59.99 (OCH_3_), 60.76 (OCH_3_), 63.36 (CH_2_), 75.85 (CH-OH), 102.58 (CH), 114.26 (2×CH), 128.53 (2×CH), 130.68 (C), 138.10 (C), 140.45 (C), 142.65 (C-O), 150.12 (C-O), 154.13 (C-O), 157.29 (C-OEt) ppm. HRMS (EI): Found 367.1520 [M+Na]^+^; C_20_H_24_NaO_5_ requires 367.1521.

**3-(4-Fluorophenyl)-4,5,6-trimethoxy-2,3-dihydro-1*H*-inden-1-ol (25e):** As per general method V, 3-(4-fluorophenyl)-4,5,6-trimethoxy-2,3-dihydro-1*H*-inden-1-one (**24e**) (1 eq, 2.8 mmol, 0.9 g) was reacted with sodium borohydride (2 eq, 5.7 mmol, 0.22 g) in methanol (20 mL) and THF (20 mL) to afford the desired product as a yellow oil. Yield: 94% (0.83 g). IR: ν_max_ (ATR) cm^−1^: 3239, 2980, 2958, 1691, 1597, 1508, 1462, 1416, 1338, 1271, 1210, 1184, 1122, 1043, 981, 843, 808, 760, 660, 591. ^1^H NMR (400 MHz, CDCl_3_) δ 1.92 (dt, *J* = 14.0, 5.2 Hz, 1 H, CH_2_), 2.99 (ddd, *J* = 13.9, 8.5, 7.1 Hz, 1 H, CH_2_), 3.39 (s, 3 H, OCH_3_), 3.81 (s, 3 H, OCH_3_), 3.90 (s, 3 H, OCH_3_), 4.30 (dd, *J* = 8.5, 5.6 Hz, 1 H, CH), 5.19 (dd, *J* = 7.1, 5.0 Hz, 1 H, CH-OH), 6.81 (s, 1 H, Ar-H) 6.94–7.00 (m, 2 H, Ar-H), 7.19–7.24 (m, 2 H, Ar-H). ^13^C NMR (101 MHz, CDCl_3_) 46.12 (CH_2_), 46.28 (C), 56.12 (OCH_3_), 59.93 (OCH_3_), 60.76 (OCH_3_), 75.74 (CH-OH), 102.58 (CH), 114.87 (2×CH), 129.10 (2×CH), 130.28 (C), 140.39 (C), 141.69 (C), 142.63 (C-O), 150.01 (C-O), 154.33 (C-O), 162.54 (C-F) ppm. LRMS (EI): Found 317.24 (M-H)^+^; C_18_H_18_FO_4_ requires 318.13.

**4,5,6-Trimethoxy-3-phenyl-2,3-dihydro-1*H*-inden-1-ol (25f):** As per general method V, 4,5,6-trimethoxy-3-phenyl-2,3-dihydro-1*H*-inden-1-one (**24f**) (1 eq, 2.74 mmol, 0.82 g) was reacted with sodium borohydride (2 eq, 5.48 mmol, 0.24 g) in methanol (20 mL) and THF (20 mL) to afford the desired product as a yellow oil. Yield: 43% (0.35 g). IR: ν_max_ (ATR) cm^−1^: 3276, 2965, 2937, 1601, 1463, 1410, 1331, 1190, 1112, 1041, 1016, 991, 969, 832, 749, 749, 703, 670, 553. ^1^H NMR (400 MHz, CDCl_3_) δ 1.90–1.97 (m, 1 H, CH_2_), 2.96 (ddd, *J* = 13.7, 8.3, 7.5 Hz, 1 H, CH_2_), 3.32 (s, 3 H, OCH_3_), 3.79 (s, 3 H, OCH_3_), 3.86 (s, 3 H, OCH_3_), 4.28 (dd, *J* = 8.5, 6.0 Hz, 1 H, CH), 5.15 (t, *J* = 6.2 Hz, 1 H, CH-OH), 6.81 (s, 1 H, Ar-H), 7.14–7.19 (m, 1 H, Ar-H), 7.21–7.29 (m, 4 H, Ar-H). ^13^C NMR (101 MHz, CDCl_3_) 46.22 (CH_2_), 46.7 (CH), 55.94 (OCH_3_), 59.68 (OCH_3_), 60.60 (OCH_3_), 75.46 (CH-OH), 102.54 (CH), 127.04 (CH), 127.54 (2×CH), 128.10 (2×CH), 130.24 (C), 140.61 (C), 142.35 (C), 145.89 (C-O), 149.87 (C-O), 153.97 (C-O) ppm. HRMS (EI): Found 323.1251 [M+Na]^+^; C_18_H_20_NaO_4_ requires 323.1259.

**4,5,6-Trimethoxy-3-(4-nitrophenyl)-2,3-dihydro-1*H*-inden-1-ol (25g):** As per general method V, 4,5,6-trimethoxy-3-(4-nitrophenyl)-2,3-dihydro-1*H*-inden-1-one (**24g**) (1 eq, 2.74 mmol, 0.82 g) was reacted with sodium borohydride (2 eq, 5.48 mmol, 0.24 g) of in methanol (20 mL) and THF (20 mL) to afford the desired product as an orange resin. Yield: 93% (0.79 g). IR: ν_max_ (ATR) cm^−1^: 3462, 2969, 2938, 2901, 1595, 1513, 1479, 1338, 1233, 1112, 1049, 1022, 853, 746, 593, 630, 616, 593. ^1^H NMR (400 MHz, CDCl_3_) δ 1.94 (dt, *J* = 14.1, 5.0 Hz, 1 H, CH_2_), 2.99–3.07 (m, 1 H, CH_2_), 3.43 (s, 3 H, OCH_3_), 3.80 (s, 3 H, OCH_3_), 3.91 (s, 3 H, OCH_3_), 4.41 (dd, *J* = 8.7, 5.4 Hz, 1 H, CH), 5.23–5.29 (m, 1 H, CH-OH), 6.82 (s, 1 H, Ar-H), 7.44 (d, *J* = 8.7 Hz, 2 H, Ar-H), 8.15 (d, *J* = 8.3 Hz, 2 H, Ar-H). ^13^C NMR (101 MHz, CDCl_3_) 154.78 (C-O), 153.76 (C-O), 149.78 (C), 146.38 (C-NO_2_), 142.55 (C-O), 140.39 (C), 128.58 (2×CH), 123.98 (2×CH), 123.58 (C), 102.59 (CH), 75.70 (CH-OH), 60.79 (OCH_3_), 59.94 (OCH_3_), 56.14 (OCH_3_), 46.71 (CH), 45.68 (CH_2_) ppm. HRMS (EI): Found 344.3214 [M-H]^+^; C_18_H_28_NO_6_ requires 344.1134.

**4,5,6-Trimethoxy-3-(4-methoxy-3-nitrophenyl)-2,3-dihydro-1*H*-inden-1-ol (25h):** As per general method V, 4,5,6-trimethoxy-3-(4-methoxy-3-nitrophenyl)-2,3-dihydro-1*H*-inden-1-one (**24h**) (1 eq, 2.66 mmol, 0.99 g) was reacted with sodium borohydride (2 eq, 5.32 mmol, 0.20 g) in methanol (20 mL) and THF (20 mL) to afford the desired product as a brown oil. Yield: 94% (0.93 g) HPLC 86%. IR: ν_max_ (ATR) cm^−1^: 3448, 2929, 2852, 1620, 1526, 1500, 1478, 1464, 1413, 1235, 1088, 1050, 975, 907, 836, 820, 729, 680, 582. ^1^H NMR (400 MHz, CDCl_3_) δ 1.85–1.93 (m, 1 H, CH_2_), 2.96 (ddd, *J* = 13.9, 8.5, 7.1 Hz, 1 H, CH_2_), 3.46 (s, 3 H, OCH_3_), 3.78 (s, 3 H, OCH_3_), 3.87 (s, br, 3 H, OCH_3_), 3.91 (s, 3 H, OCH_3_), 4.28 (dd, *J* = 8.3, 5.4 Hz, 1 H, CH), 5.19 (br. s., 1 H, CH-OH), 6.78 (s, 1 H, Ar-H), 6.98 (d, *J* = 8.7 Hz, 1 H, Ar-H), 7.43 (dd, *J* = 8.5, 2.3 Hz, 1 H, Ar-H), 7.77 (d, *J* = 2.5 Hz, 1 H, Ar-H). ^13^C NMR (101 MHz, CDCl_3_) 45.70 (CH_2_), 45.73 (CH), 56.14 (OCH_3_), 56.52 (OCH_3_), 60.06 (OCH_3_), 60.81 (OCH_3_), 75.55 (CH), 102.65 (CH), 113.35 (CH), 124.85 (CH), 129.34 (C), 133.45 (CH), 138.41 (C), 140.34 (C-NO_2_), 142.57 (C-O), 149.89 (C), 151.28 (2×C-O), 154.62 (C-O) ppm. HRMS (EI): Found 398.1203 [M+Na]^+^; C_19_H_21_NNaO_7_ requires 398.1216.

**3-(4-Hydroxyphenyl)-4,5,6-trimethoxy-2,3-dihydro-1*H*-inden-1-ol (25i):** As per general method V, 3-(4-hydroxyphenyl)-4,5,6-trimethoxy-2,3-dihydro-1*H*-inden-1-one (**24i**) (1 eq, 0.31 mmol, 0.1 g) was reacted with sodium borohydride (2 eq, 0.62 mmol, 0.02 g) in methanol (10 mL) and THF (10 mL) to afford the desired product as a clear oil. Yield: 60% (0.054 g). IR: ν_max_ (ATR) cm^−1^: 3514, 3207, 2987, 2972, 2941, 1590, 1456, 1414, 1334, 1234, 1112, 1052, 993, 977, 852, 702, 656, 598. ^1^H NMR (400 MHz, CDCl_3_) δ 7.05–6.98 (m, 2 H, Ar-H), 6.78 (s, 1 H, Ar-H), 6.69–6.64 (m, 2 H, Ar-H), 5.14 (dd, *J* = 6.9, 5.0 Hz, 1 H, CH), 4.23 (dd, *J* = 8.3, 5.6 Hz, 1 H, CH-OH), 3.84 (s, 3 H, OCH_3_), 3.78 (s, 3 H, OCH_3_), 3.35 (s, 3 H, OCH_3_), 2.97–2.87 (m, 1 H, CH_2_), 1.88 (dt, *J* = 13.8, 5.2 Hz, 1 H, CH_2_). ^13^C NMR (101 MHz, CDCl_3_) 154.10 (C-OH), 154.03 (C-O), 150.02 (C-O), 142.58 (C-O), 140.37 (C), 138.08 (C), 130.64 (C), 128.68 (2×CH), 115.13 (2×CH), 102.66 (CH), 75.84 (CH-OH), 60.79 (OCH_3_), 59.99 (OCH_3_), 56.10 (OCH_3_), 46.37 (CH), 46.02 (CH_2_) ppm. LRMS (EI): Found 315.29 (M-H)^+^; C_18_H_19_O_5_ requires 315.12.

#### 4.1.6. General Method VI: Preparation of Series 3 1-(3-(aryl)-4,5,6-Trimethoxy-2,3-Dihydro-1*H*-Inden-1-yl)-1*H*-1,2,4-Triazoles (**26a–e**)

To a solution of the appropriate 3-aryl-4,5,6-trimethoxy-2,3-dihydro-1*H*-inden-1-ol (1 eq) in toluene (60 mL), 1,2,4-triazole (3 eq) and *p*-toluenesulfonic acid (200 mg, 0.61 eq) were added. The reaction mixture was heated at reflux for 4 h in a Biotage open vessel microwave reactor (90–250 W) equipped with a Dean-Stark trap. On completion of the reaction, the toluene was evaporated and the crude product was then dissolved in ethyl acetate (30 mL), followed by washing with water (20 mL) and brine (10 mL). The final solution was then dried with anhydrous sodium sulfate, the solution was filtered, and then concentrated. Purification of the crude product by flash chromatography (*n*-hexane/ethyl acetate, 1:1) over silica gel gave the desired product.

**5-(5,6,7-Trimethoxy-3-(1*H*-1,2,4-triazol-1-yl)-2,3-dihydro-1*H*-inden-1-yl)phenol (26a):** As per general method VI, 3-(3-hydroxy-4-methoxyphenyl)-4,5,6-trimethoxy-2,3-dihydro-1*H*-inden-1-ol (**25a**) (1 eq, 1.36 mmol, 0.47 g) was reacted with 1,2,4-triazole (3 eq, 4.08 mmol, 0.28 g) and *p*-TSA (0.15 g) of in toluene (60 mL). The reaction was carried out in an open vessel microwave reactor heated to 130 °C, under reflux, for 4 h. When the reaction was complete by tlc, the reaction mixture was cooled to room temperature and treated as outlined in general method VI. Purification of the crude product with flash column chromatography required a mobile phase of *n*-hexane/ethyl acetate 1:9 to afford the desired product as a yellow oil. Yield: 30% (0.16 g) (HPLC: 94%). IR: ν_max_ (ATR) cm^−1^: 3118, 2939, 1597, 1504, 1465, 1413, 1338, 1237, 1216, 1116, 1025, 983, 925, 802, 761, 676 ^1^H NMR (400 MHz, CDCl_3_) δ 2.38 (dt, *J* = 13.9, 6.3 Hz, 1 H, CH_2_), 2.88 (ddd, *J* = 13.5, 8.5, 7.1 Hz, 1 H, CH_2_), 3.54 (s, 3 H, OCH_3_), 3.78 (s, 3 H, OCH_3_), 3.82 (s, 3 H, OCH_3_), 3.84 (s, 3 H, OCH_3_), 4.40 (dd, *J* = 8.5, 6.4 Hz, 1 H, CH), 5.83 (dd, *J* = 8.5, 6.4 Hz, 1 H, CH-O-N), 6.40 (s, 1 H, Ar-H), 6.64 (d, *J* = 2.5 Hz, 1 H, Ar-H), 6.75 (d, *J* = 4.6 Hz, 1 H, Ar-H), 6.81 (d, *J* = 2.1 Hz, 1 H, Ar-H), 7.98 (s, 1 H, CH-N), 8.06 (s, 1 H, CH-N). ^13^C NMR (101 MHz, CDCl_3_) 154.58 (C-O), 152.00 (CH-N), 150.28 (C-O), 145.64 (C-O), 145.21 (C-O), 143.07 (CH-N), 142.18 (C-O), 137.92 (C), 134.93 (C), 131.40 (C), 118.61 (CH), 113.20 (CH), 110.56 (CH), 102.38 (CH), 64.25 (CH), 60.80 (OCH_3_), 60.26 (OCH_3_), 56.17 (2×OCH_3_), 46.03 (CH), 44.09 (CH_2_) ppm. HRMS (EI): Found 396.1557 [M-H]^+^; C_21_H_22_N_3_O_5_ requires 396.1565.

**1-(4,5,6-Trimethoxy-3-(3,4,5-trimethoxyphenyl)-2,3-dihydro-1*H*-inden-1-yl)-1*H*-1,2,4-triazole (26b):** As per general method VI, 4,5,6-trimethoxy-3-(3,4,5-trimethoxyphenyl)-2,3-dihydro-1*H*-inden-1-ol (**25b**) (1 eq, 1.00 mmol, 0.39 g) was reacted with 1,2,4-triazole (3 eq, 3.00 mmol, 0.21 g) and *p*-TSA (0.15 g) in toluene (60 mL). The reaction was carried out in an open vessel microwave reactor heated to 130 °C, under reflux, for 4 h. Upon completion, the reaction mixture was cooled to room temperature and treated as outlined in general method VI. Purification of the crude product via flash column chromatography required a mobile phase of *n*-hexane/ethyl acetate 1:9 to afford the desired product as a red oil. Yield: 40% (0.18 g). IR: ν_max_ (ATR) cm^−1^: 2937, 2837, 1589, 1504, 1460, 1412, 1273, 1235, 1114, 1066, 1043, 956, 790, 702, 663. ^1^H NMR (400 MHz, CDCl_3_) δ 2.48 (d, *J* = 14.1 Hz, 1 H, CH_2_), 3.25 (s, 1 H, CH_2_), 3.57 (s, 3 H, OCH_3_), 3.80 (s, 6 H, 2×OCH_3_), 3.81 (s, 6 H, 2×OCH_3_), 3.85 (s, 3 H, OCH_3_), 4.67 (s, 1 H, CH), 6.02 (s, 1 H, CH-N-R), 6.46 (s, 1 H, Ar-H), 6.48 (s, 2 H, Ar-H), 8.01 (s, 1 H, CH-N), 8.15 (s, 1 H, CH-N). ^13^C NMR (101 MHz, CDCl_3_) 44.04 (CH_2_), 47.54 (C), 56.06 (3×OCH_3_), 60.26 (OCH_3_), 60.78 (2×OCH_3_), 64.17 (CH-N-R), 102.43 (CH), 104.67 (2×CH), 131.40 (C), 135.15 (C), 136.62 (C-O), 140.22 (CH-N), 143.01 (C-O), 150.31 (C-O), 152.18 (CH-N), 153.18 (C-O), 154.62 (2×C-O) ppm. HRMS (EI): Found 464.1780 [M+Na]^+^; C_23_H_27_N_3_NaO_6_ requires 464.1798.

**1-(3-(3,4-Dimethoxyphenyl)-4,5,6-trimethoxy-2,3-dihydro-1*H*-inden-1-yl)-1*H*-1,2,4-triazole (26c):** As per general method VI, 3-(3,4-dimethoxyphenyl)-4,5,6-trimethoxy-2,3-dihydro-1*H*-inden-1-ol (**25c**) (1 eq, 1.11 mmol, 0.40 g) was reacted with 1,2,4-triazole (3 eq, 3.33 mmol, 0.23 g) and *p*-TSA (0.15 g) in toluene (60 mL). The reaction was carried out in an open vessel microwave reactor heated to 130 °C, under reflux, for 4 h. Upon completion, the reaction mixture was cooled to room temperature and treated as outlined in general method VI. Purification of the crude product via flash column chromatography required a mobile phase of *n*-hexane/ethyl acetate 3:7 to afford the desired product as an orange resin. Yield: 54% (0.25 g) (HPLC: 93%). IR: ν_max_ (ATR) cm^−1^: 3061, 2962, 2937, 2840, 1747, 1588, 1504, 1461, 1412, 1233, 1116, 984, 835, 742, 664, 553. ^1^H NMR (400 MHz, CDCl_3_) δ 2.63 (ddd, *J* = 13.5, 7.7, 4.2 Hz, 1 H, CH_2_), 3.24 (dt, *J* = 14.1, 8.7 Hz, 1 H, CH_2_), 3.51 (s, 3 H, OCH_3_), 3.79 (s, 3 H, OCH_3_), 3.82 (s, 3 H, OCH_3_), 3.83 (s, 3 H, OCH_3_), 3.85 (s, 3 H, OCH_3_), 4.66 (dd, *J* = 8.3, 4.2 Hz, 1 H, CH), 6.02 (t, *J* = 7.1 Hz, 1 H, CH-N-R), 6.44 (s, 1 H, Ar-H), 6.60 (dd, *J* = 8.3, 2.1 Hz, 1 H, Ar-H), 6.67 (d, *J* = 1.7 Hz, 1 H, Ar-H), 6.79 (s, 1 H, Ar-H), 7.99 (s, 1 H, CH-N), 8.11 (s, 1 H, CH-N). ^13^C NMR (101 MHz, CDCl_3_) 44.13 (CH_2_), 46.82 (CH), 55.81 (3×OCH_3_), 60.08 (OCH_3_), 60.76 (OCH_3_), 64.19 (CH-N-R), 102.43 (CH), 110.72 (CH), 111.14 (CH), 119.51 (C), 131.38 (CH), 131.53 (C), 134.74 (C), 143.07 (C-O), 143.25 (CH-N), 147.65 (2×C-O), 150.31 (C-O), 152.23 (CH-N), 154.56 (C-O) ppm. HRMS (EI): Found 412.1869 [M+H]^+^; C_22_H_26_N_3_O_5_ requires 412.1872.

**1-(3-(4-Fluorophenyl)-4,5,6-trimethoxy-2,3-dihydro-1*H*-inden-1-yl)-1*H*-1,2,4-triazole (26d):** As per general method VI, 3-(4-fluorophenyl)-4,5,6-trimethoxy-2,3-dihydro-1*H*-inden-1-ol (**25e**) (1 eq, 2.61 mmol, 0.83 g) was reacted with 1,2,4-triazole (3 eq, 7.83 mmol, 0.55 g) and *p*-TSA (0.15 g) in toluene (60 mL). The reaction was carried out in an open vessel microwave reactor heated to 130 °C, under reflux, for 4 h. Upon completion, the reaction mixture was cooled to room temperature and treated as outlined in general method VI. Purification of the crude product via flash column chromatography (eluant: *n*-hexane/ethyl acetate 1:9) to afford the desired product as a yellow resin. Yield: 37% (0.36 g) (HPLC: 86%). IR: ν_max_ (ATR) cm^−1^: 2937, 2837, 1600, 1579, 1504, 1461, 1432, 1236, 1118, 1004, 904, 891, 833, 700, 675. ^1^H NMR (400 MHz, CDCl_3_) δ 2.41 (dt, *J* = 14.0, 6.9 Hz, 1 H, CH_2_), 2.88–2.95 (m, 1 H, CH_2_), 3.39 (s, 3 H, OCH_3_), 3.79 (s, 3 H, OCH_3_), 3.82 (s, 3 H, OCH_3_), 4.46 (dd, *J* = 8.3, 7.5 Hz, 1 H, CH), 5.84–5.90 (m, 1 H, CH-N-R), 6.45 (s, 1 H, Ar-H), 6.96–6.98 (m, 2 H, Ar-H), 7.06–7.11 (m, 2 H, Ar-H), 8.01 (s, 1 H, CH-N), 8.15 (s, 1 H, CH-N). ^13^C NMR (101 MHz, CDCl_3_) 43.45 (CH_2_), 46.34 (CH), 56.17 (OCH_3_), 60.15 (OCH_3_), 60.76 (OCH_3_), 64.46 (CH-N-R), 102.48 (CH), 115.20 (CH), 115.42 (CH), 128.53 (CH), 128.60 (CH), 134.95 (C), 140.27 (C), 142.10 (C-O), 143.16 (CH-N), 150.24 (C-O), 152.26 (CH-N), 154.78 (C-O), 160.31 (C-F) ppm. HRMS (EI): Found 370.1562 [M+H]^+^; C_20_H_21_FN_3_O_3_ requires 370.1569.

**1-(4,5,6-Trimethoxy-3-phenyl-2,3-dihydro-1*H*-inden-1-yl)-1*H*-1,2,4-triazole (26e):** As per general method VI, 4,5,6-trimethoxy-3-phenyl-2,3-dihydro-1*H*-inden-1-ol (**25f**) (1 eq, 1.22 mmol, 0.37 g) was reacted with 1,2,4-triazole (3 eq, 3.67 mmol, 0.25 g) and *p*-TSA (0.15 g) in toluene (60 mL). The reaction was carried out in an open vessel microwave reactor heated to 130 °C, under reflux, for 4 h. Upon completion, the reaction mixture was cooled to room temperature and treated as outlined in general method VI. Purification of the crude product via flash column chromatography (eluant: *n*-hexane/ethyl acetate 1:9) to afford the desired product as a yellow resin. Yield: 48% (0.2 g). IR: ν_max_ (ATR) cm^−1^: 2935, 2836, 1599, 1579, 1460, 1451, 1432, 1329, 1236, 1122, 1006, 955, 908, 834, 760, 729, 699, 660. ^1^H NMR (400 MHz, CDCl_3_) δ 2.42 (dt, *J* = 14.0, 6.9 Hz, 1 H, CH_2_), 2.90 (ddd, *J* = 13.7, 8.1, 6.2 Hz, 1 H, CH_2_), 3.44 (s, 3 H, OCH_3_), 3.77 (s, 3 H, OCH_3_), 3.81 (s, 3 H, OCH_3_), 4.69 (dd, *J* = 8.7, 4.2 Hz, 1 H, CH), 6.02 (t, *J* = 7.1 Hz, 1 H, CH-N-R), 6.42 (s, 1 H, Ar-H), 7.08 (s, 1 H, Ar-H), 7.16–7.26 (m, 5 H, Ar-H), 8.03 (s, 1 H, CH-N), 8.10 (s, 1 H, CH-N). ^13^C NMR (101 MHz, CDCl_3_) 44.00 (CH_2_), 47.08 (CH), 56.16 (OCH_3_), 60.10 (OCH_3_), 60.77 (OCH_3_), 64.57 (CH), 102.43 (CH), 126.56 (CH), 127.16 (2×CH), 128.53 (2×CH), 131.52 (C), 135.10 (C), 142.13 (C), 143.13 (CH-N), 144.59 (C-O), 150.31 (C-O), 152.24 (CH-N), 154.65 (C-O) ppm. HRMS (EI): Found 352.1656 [M+H]^+^; C_20_H_22_N_3_O_3_ requires 352.1661.

#### 4.1.7. General Method VII: Preparation of Series 4 1-(3-aryl-4,5,6-Trimethoxy-2,3-Dihydro-1*H*-Inden-1-yl)-1*H*-Imidazoles (**27a–i**)

CDI (1,1′-Carbonyldiimidazole) (1.3 eq) was added to a solution of the appropriate 3-aryl- 4,5,6-trimethoxy-2,3-dihydro-1*H*-inden-1-ol (1 eq) in dry acetonitrile (60 mL). The reaction mixture was heated at reflux for 3 h under nitrogen as described above. Following evaporation of the solvent, the crude product was dissolved in DCM (30 mL) and washed with water (20 mL) and brine (10 mL). The final solution was dried (anhydrous sodium sulfate) and concentrated. Purification of the crude product by flash chromatography over silica gel (eluent: *n*-hexane/ethyl acetate 1:1) gave the desired product.

**5-(3-(1*H*-Imidazol-1-yl)-5,6,7-trimethoxy-2,3-dihydro-1*H*-inden-1-yl)-2-methoxyphenol (27a):** As per general method VII, 3-(3-hydroxy-4-methoxyphenyl)-4,5,6-trimethoxy-2,3-dihydro-1*H*-inden-1-ol (**25a**) (1 eq, 1.04 mmol, 0.36 g) was reacted with CDI (1.3 eq, 1.35 mmol, 0.22 g) in dry ACN (30 mL) at reflux (75 °C), under nitrogen. Upon completion, the reaction mixture was cooled to room temperature and treated as outlined in general method VII. The crude product was purified via flash column chromatography with a mobile phase of *n*-hexane/ethyl acetate 1:9 to afford the desired product as a brown oil. Yield: 21% (0.08 g) (HPLC: 86%). IR: ν_max_ (ATR) cm^−1^: 3113, 2937, 2837, 2720, 1594, 1480, 1464, 1433, 1336, 1267, 1218, 1115, 1064, 1043, 909, 866, 801, 760, 660, 643. ^1^H NMR (400 MHz, CDCl_3_) δ 2.57–2.64 (m, 2 H, CH_2_), 3.57 (s, 3 H, OCH_3_), 3.77 (s, 3 H, OCH_3_), 3.84 (s, 3 H, OCH_3_), 3.85 (s, 3 H, OCH_3_), 4.59 (dd, *J* = 7.5, 3.7 Hz, 1 H, CH), 5.78 (t, *J* = 7.7 Hz, 1 H, CH-N-R), 6.36 (s, 1 H, Ar-H), 6.57 (dd, *J* = 8.1, 2.3 Hz, 1 H, Ar-H), 6.62 (d, *J* = 2.1 Hz, 1 H, Ar-H), 6.77 (d, *J* = 8.3 Hz, 1 H, Ar-H), 6.88 (br. s., 1 H, CH-N), 7.10 (br. s., 1 H, CH-N), 7.58 (br. s., 1 H, CH-N). ^13^C NMR (101 MHz, CDCl_3_) 45.71 (CH_2_), 45.76 (CH), 55.90 (OCH_3_), 56.16 (OCH_3_), 60.31 (2×OCH_3_), 60.79 (CH-N-R), 102.36 (CH), 110.70 (CH), 113.35 (CH), 118.35 (C, 2×CH), 130.80 (C, CH-N), 136.37 (C), 137.81 (CH-N), 142.72 (C-O), 145.43 (C-O), 145.88 (C-OH), 150.06 (C-O), 154.55 (C-O). HRMS (EI): Found 395.1613 [M-H]^+^; C_22_H_23_N_2_O_5_ requires 396.1607.

**1-(4,5,6-Trimethoxy-3-(3,4,5-trimethoxyphenyl)-2,3-dihydro-1*H*-inden-1-yl)-1*H*-imidazole (27b):** As per general method VII, 4,5,6-trimethoxy-3-(3,4,5-trimethoxyphenyl)-2,3-dihydro-1*H*-inden-1-ol (**25b**) (1 eq, (0.51 mmol, 0.2 g) was reacted with CDI (1.3 eq, 0.66 mmol, 0.1 g) in dry ACN (30 mL) at reflux (75 °C), under nitrogen. Upon completion, the reaction mixture was cooled to room temperature and treated as outlined in general method VII. The crude product was purified via flash column chromatography (eluent: *n*-hexane/ethyl acetate, 1:9) to afford the desired product as a red oil. Yield: 31% (0.07 g). IR: ν_max_ (ATR) cm^−1^: 2938, 1585, 1504, 1459, 1415, 1331, 1185, 1121, 1004, 829, 773, 699, 677, 662. ^1^H NMR (400 MHz, CDCl_3_) δ 2.19 (d, *J* = 7.1 Hz, 1 H, CH_2_), 3.22 (s, 1 H, CH_2_), 3.79–3.80 (m, 12 H, 4×OCH_3_), 3.82–3.84 (m, 6 H, 2×OCH_3,_) 4.37 (s, 1 H, CH), 5.61 (s, 1 H, CH-N-R), 6.39 (s, 1 H, Ar-H), 6.41 (s, 1 H, Ar-H), 6.43 (s, 1 H, CH-N), 6.87 (s, 1 H, CH-N), 7.11 (d, *J* = 3.7 Hz, 1 H, Ar-H), 7.56 (s, 1 H, CH-N). ^13^C NMR (101 MHz, CDCl_3_) 45.56 (CH), 45.88 (CH_2_), 56.25 (3×OCH_3_), 60.28 (OCH_3_), 60.83 (OCH_3_), 63.05 (OCH_3_), 63.50 (CH-N-R), 102.26 (3×CH), 114.57 (C, CH-N), 127.98 (CH-N), 131.81 (C), 134.08 (C-O), 135.89 (C, CH-N), 143.56 (C-O), 150.34 (C-O), 154.96 (C-O), 157.76 (2×C-O) ppm. HRMS (EI): Found 441.2015 [M+H]^+^; C_24_H_29_N_2_O_6_ requires 441.2025.

**1-(3-(3,4-Dimethoxyphenyl)-4,5,6-trimethoxy-2,3-dihydro-1*H*-inden-1-yl)-1*H*-imidazole (27c):** As per general method VII, 3-(3,4-dimethoxyphenyl)-4,5,6-trimethoxy-2,3-dihydro-1*H*-inden-1-ol (**25c**) (1 eq, 1.46 mmol, 0.53 g) was reacted with CDI (1.3 eq, 1.89 mmol, 0.31 g) in dry ACN (30 mL) at reflux (75 °C), under nitrogen. Upon completion, the reaction mixture was cooled to room temperature and treated as outlined in general method VII. The crude product was purified via flash column chromatography (eluant: *n*-hexane/ethyl acetate 3:7) to afford the desired product as a brown oil. Yield: 21% (0.13 g) (HPLC: 88%). ^1^H NMR (400 MHz, CDCl_3_) δ 2.13–2.21 (m, 1 H, CH_2_), 3.22 (dt, *J* = 13.9, 8.4 Hz, 1 H, CH_2_), 3.56 (s, 3 H, OCH_3_), 3.82 (s, 3 H, OCH_3_), 3.84 (s, 3 H, OCH_3_), 3.85 (s, 6 H, 2×OCH_3_), 4.64 (dd, *J* = 7.7, 3.9 Hz, 1 H, CH), 5.77 (t, *J* = 7.5 Hz, 1 H, CH-N-R), 6.40 (s, 1 H, CH-N), 6.55 (dd, *J* = 8.3, 2.1 Hz, 1 H, Ar-H), 6.66 (d, *J* = 2.1 Hz, 1 H, Ar-H), 6.79 (d, *J* = 7.1 Hz, 1 H, Ar-H), 6.87 (s, 1 H, Ar-H), 7.10 (br. s., 2 H, CH-N), 7.56 (s, 1 H, CH-N). ^13^C NMR (101 MHz, CDCl_3_) 45.84 (CH_2_), 46.75 (CH), 55.88 (2×OCH_3_), 56.17 (OCH_3_), 60.36 (OCH_3_), 60.83 (OCH_3_), 61.58 (CH-N-R), 102.43 (CH), 110.79 (CH), 111.15 (CH), 117.44 (CH), 118.70 (CH-N), 119.38 (C) 129.81 (CH-N), 130.71 (C), 136.44 (C), 137.60 (CH-N), 142.76 (C-O), 147.60 (C-O), 148.98 (C-O), 150.14 (C-O), 154.58 (C-O) ppm. HRMS (EI): Found 411.1919 [M+H]^+^; C_23_H_27_N_2_O_5_ requires 411.1914.

**1-(3-(4-Ethoxyphenyl)-4,5,6-trimethoxy-2,3-dihydro-1*H*-inden-1-yl)-1*H*-imidazole (27d):** As per general method VII, 3-(4-ethoxyphenyl)-4,5,6-trimethoxy-2,3-dihydro-1*H*-inden-1-ol (**25d**) (1 eq, 1.31 mmol, 0.45 g) was reacted with CDI (1.3 eq, 1.7 mmol, 0.27 g) in dry ACN (30 mL) at reflux (75 °C), under nitrogen. Upon completion, the reaction mixture was cooled to room temperature and treated as outlined in general method VII. The crude product was purified via flash column chromatography (ethyl acetate: methanol 9:1) to afford the desired product as an orange resin. Yield: 53% (0.27 g). IR: ν_max_ (ATR) cm^−1^: 2979, 2940, 1671, 1510, 1467, 1412, 1339, 1197, 1174, 1113, 1044, 980, 824, 797, 718. ^1^H NMR (400 MHz, CDCl_3_) δ 1.39–1.42 (m, 3 H, CH_3_), 2.16 (dt, *J* = 13.9, 6.7 Hz, 1 H, CH_2_), 3.30 (dt, *J* = 14.1, 8.5 Hz, 1 H, CH_2_), 3.50 (s, 3 H, OCH_3_), 3.81 (s, 3 H, OCH_3_), 3.86 (s, 3 H, OCH_3_), 4.01 (dd, *J* = 7.1, 2.1 Hz, 2 H, CH_2_), 4.65 (dd, *J* = 7.9, 4.6 Hz, 1 H, CH), 5.92 (t, *J* = 7.1 Hz, 1 H, CH-N-R), 6.42 (s, 1 H, CH-N), 6.82–6.86 (m, 3 H, Ar-H), 6.98 (d, *J* = 8.7 Hz, 2 H, Ar-H), 7.07 (d, *J* = 8.3 Hz, 1 H, Ar-H), 8.28 (s, 1 H, CH-N). ^13^C NMR (101 MHz, CDCl_3_) 14.81 (CH_3_), 45.53 (CH_2_), 45.91 (CH), 56.26 (OCH_3_), 60.28 (OCH_3_), 60.83 (OCH_3_), 63.43 (CH_2_), 63.63 (CH-N-R), 102.28 (CH), 114.57 (2×CH), 118.51 (C, CH-N), 127.99 (3×CH), 131.87 (C), 133.98 (C), 136.07 (CH-N), 143.58 (C-O), 150.34 (C-O), 154.99 (C-O), 157.76 (C-OEt) ppm. HRMS (EI): Found 395.1970 [M+H]^+^; C_23_H_27_N_2_O_4_ requires 395.1971.

**1-(3-(4-Fluorophenyl)-4,5,6-trimethoxy-2,3-dihydro-1*H*-inden-1-yl)-1*H*-imidazole (27e):** As per general method VII, 3-(4-fluorophenyl)-4,5,6-trimethoxy-2,3-dihydro-1*H*-inden-1-ol (**25e**) (1 eq, 1.1 mmol, 0.35 g) was reacted with CDI (1.3 eq, 1.43 mmol, 0.23 g) in dry ACN (30 mL) at reflux (75 °C), under nitrogen. Upon completion, the reaction mixture was cooled to room temperature and treated as outlined in general method VII. The crude product was purified via flash column chromatography (eluant: dichloromethane/ethyl acetate, 2:1) to afford the desired product as an orange resin. Yield: 24% (0.09 g). IR: ν_max_ (ATR) cm^−1^: 2968, 2840, 1597, 1507, 1464, 1414, 1332, 1276, 1224, 1116, 1019, 905, 834, 798, 766, 728, 691, 661. ^1^H NMR: (400 MHz, CDCl_3_) δ 2.09 (dt, *J* = 13.7, 7.9 Hz, 1 H, CH_2_), 3.14–3.23 (m, 1 H, CH_2_), 3.50 (s, 3 H, OCH_3_), 3.75 (s, 3 H, OCH_3_), 3.82 (s, 3 H, OCH_3_), 4.64 (dd, *J* = 8.3, 3.7 Hz, 1 H, CH), 5.75 (t, *J* = 7.5 Hz, 1 H, CH-N-R), 6.38 (s, 1 H, Ar-H), 6.84 (s, 1 H, CH-N), 6.95–6.97 (m, 2 H, Ar-H), 6.99–7.03 (m, 2 H, Ar-H), 7.06 (s, 1 H, CH-N), 7.53 (s, 1 H, CH-N). ^13^C NMR: (101 MHz, CDCl_3_) 45.71 (CH_2_), 46.20 (CH), 56.18 (OCH_3_), 59.86 (OCH_3_), 60.78 (OCH_3_), 61.57 (CH-N-R), 102.44 (CH), 115.17 (2×CH), 115.39 (C), 117.40 (CH-N), 128.49 (CH-N), 128.84 (2×CH), 130.56 (C), 136.21 (CH-N), 140.59 (C), 142.79 (C-O), 150.00 (C-O), 154.77 (C-O), 160.26 (C-F) ppm. HRMS (EI): Found 369.1605 [M+H]^+^; C_21_H_22_FN_2_O_5_ requires 369.1614.

**1-(4,5,6-Trimethoxy-3-phenyl-2,3-dihydro-1*H*-inden-1-yl)-1*H*-imidazole (27f):** As per general method VII, 4,5,6-trimethoxy-3-phenyl-2,3-dihydro-1*H*-inden-1-ol (**25f**) (1 eq, 1.33 mmol, 0.4 g) was reacted with CDI (1.3 eq, 1.73 mmol, 0.28 g) in dry ACN (30 mL) at reflux (75 °C), under nitrogen. Upon completion, the reaction mixture was cooled to room temperature and treated as outlined in general method VII. The crude product was purified via flash column chromatography (eluant: *n*-hexane/ethyl acetate, 3:7) to afford the desired product as a brown oil. Yield: 5% (0.02 g). IR: ν_max_ (ATR) cm^−1^: 3060, 2970, 2937, 1601, 1480, 1465, 1412, 1336, 1227, 1194, 1115, 1075, 1044, 985, 832, 800, 700. 662. ^1^H NMR (400 MHz, CDCl_3_) δ 2.61–2.67 (m, 2 H, CH_2_), 3.48 (s, 3 H, OCH_3_), 3.76 (s, 3 H, OCH_3_), 3.83 (s, 3 H, OCH_3_), 4.66 (dd, *J* = 7.9, 4.2 Hz, 1 H, CH), 5.78 (t, *J* = 7.5 Hz, 1 H, CH-N-R), 6.38 (s, 1 H, Ar-H), 6.87 (br. s., 1 H, CH-N), 7.05 (s, 1 H, CH-N), 7.06–7.10 (m, 2 H, Ar-H), 7.18–7.22 (m, 1 H, Ar-H), 7.24–7.26 (m, 1 H, Ar-H), 7.28–7.30 (m, 1 H, Ar-H), 7.55 (br. s., 1 H, CH-N). ^13^C NMR (101 MHz, CDCl_3_) 45.62 (CH_2_), 46.43 (CH), 56.16 (OCH_3_), 60.19 (2×OCH_3_), 61.68 (CH), 102.41 (CH), 126.53 (CH), 127.06 (2×CH, CH), 128.53 (2×CH), 130.79 (C), 136.43 (CH), 142.77 (C), 144.61 (C), 150.09 (C), 154.64 (C) ppm. HRMS (EI): Found 351.1710 [M+H]^+^; C_21_H_23_N_2_O_3_ requires 351.1708.

**1-(4,5,6-Trimethoxy-3-(4-nitrophenyl)-2,3-dihydro-1*H*-inden-1-yl)-1*H*-imidazole (27g):** As per general method VII, 4,5,6-trimethoxy-3-(4-nitrophenyl)-2,3-dihydro-1*H*-inden-1-ol (**25g**) (1 eq, 1.16 mmol, 0.4 g) was reacted with CDI (1.3 eq, 1.5 mmol, 0.24 g) in dry ACN (30 mL) at reflux (75 °C), under nitrogen. Upon completion, the reaction mixture was cooled to room temperature and treated as outlined in general method VII. The crude product was purified via flash column chromatography (eluant: ethyl acetate/methanol, 9:1) to afford the desired product as a brown oil. Yield: 4% (0.02 g). IR: ν_max_ (ATR) cm^−1^: 2993, 2970, 2935, 1631, 1597, 1503, 1436, 1419, 1330, 1277, 1244, 1173, 1153, 996, 916, 836, 766, 690, 663. ^1^H NMR (400 MHz, CDCl_3_) δ 2.56–2.63 (m, 1 H, CH_2_), 2.66–2.75 (m, 1 H, CH_2_), 3.54–3.55 (m, 3 H, OCH_3_), 3.78 (s, 3 H, OCH_3_), 3.81–3.82 (m, 3 H, OCH_3_), 4.71–4.77 (m, 1 H, CH), 5.79 (t, *J* = 7.1 Hz, 1 H, CH-N-R), 6.42 (s, 1 H, Ar-H), 6.96 (br. s., 1 H, CH-N), 7.15 (br. s., 1 H, CH-N), 7.35 (d, *J* = 8.7 Hz, 1 H, Ar-H), 7.52 (d, *J* = 8.7 Hz, 1 H, Ar-H), 7.57 (br. s., 1 H, CH-N), 8.12–8.16 (m, 2 H, Ar-H). ^13^C NMR (101 MHz, CDCl_3_) 45.14 (CH_2_), 46.44 (CH), 56.22 (OCH_3_), 60.23 (OCH_3_), 60.34 (OCH_3_), 60.87 (CH-N-R), 102.56 (CH), 123.81 (C), 123.87 (CH-N), 124.11 (2×CH), 127.94 (2×CH), 129.36 (CH-N), 133.04 (C), 136.21 (CH-N), 146.70 (C-NO_2_), 149.87 (C-O), 152.45 (C-O), 153.44 (C), 153.81 (C-O) ppm. HRMS (EI): Found 396.1561 [M+H]^+^; C_21_H_22_N_3_O_5_ requires 396.1560.

**1-(4,5,6-Trimethoxy-3-(4-methoxy-3-nitrophenyl)-2,3-dihydro-1*H*-inden-1-yl)-1*H*-imidazole (27h):** As per general method VII, 4,5,6-trimethoxy-3-(4-methoxy-3-nitrophenyl)-2,3-dihydro-1*H*-inden-1-ol (**25h**) (1 eq, 2.49 mmol, 0.93 g) was reacted with CDI (1.3 eq, 3.2 mmol, 0.52 g) in dry ACN (30 mL) at reflux (75 °C), under nitrogen. Upon completion, the reaction mixture was cooled to room temperature and treated as outlined in general method VII. No further purification was required to afford the desired product as a brown oil. Yield: 70% (0.74 g) IR: ν_max_ (ATR) cm^−1^: 2939, 2841, 1599, 1574, 1527, 1498, 1481, 1464, 1336, 1263, 1184, 1115, 1085, 1064, 982, 905, 819, 731, 698, 661. ^1^H NMR (400 MHz, CDCl_3_) δ 2.02–2.11 (m, 1 H, CH_2_), 3.20 (dt, *J* = 13.7, 8.1 Hz, 1 H, CH_2_), 3.47–3.50 (m, 3 H, OCH_3_), 3.80 (s, 3 H, OCH_3_), 3.82 (s, 3 H, OCH_3_), 3.93 (s, 3 H, OCH_3_), 4.39 (t, *J* = 8.1 Hz, 1 H, CH), 5.60 (t, *J* = 8.1 Hz, 1 H, CH-N-R), 6.39 (s, 1 H, Ar-H), 6.84 (s, 1 H, CH-N), 7.02 (d, *J* = 3.3 Hz, 1 H, Ar-H), 7.10 (s, 1 H, Ar-H), 7.24 (s, 1 H, CH-N), 7.30 (dd, *J* = 5.4, 2.07 Hz, 1 H, Ar-H), 7.61 (s, 1 H, CH-N). ^13^C NMR (101 MHz, CDCl_3_) 45.28 (CH_2_), 45.65 (CH), 56.18 (OCH_3_), 56.54 (OCH_3_), 60.31 (OCH_3_), 60.84 (OCH_3_), 61.43 (CH-N-R), 102.58 (CH), 113.61 (CH), 123.94 (C), 124.58 (CH-N), 128.15 (CH), 128.93 (CH), 132.85 (C), 136.20 (CH-N), 136.48 (C), 137.06 (CH-N), 139.51 (C-NO_2_), 142.73 (C-O), 149.88 (C-O), 150.18 (C-O), 151.54 (C-O) ppm. HRMS (EI): Found 426.1642 [M+H]^+^; C_21_H_24_N_3_O_6_ requires 426.1665.

**4-(3-(1*H*-Imidazol-1-yl)-5,6,7-trimethoxy-2,3-dihydro-1*H*-inden-1-yl)phenol (27i):** As per general method VII, 3-(4-hydroxyphenyl)-4,5,6-trimethoxy-2,3-dihydro-1H-inden-1-ol (**25i**) (1 eq, 0.94 mmol, 0.3 g) was reacted with CDI (1.3 eq, 1.23 mmol, 0.2 g) in dry ACN (30 mL) under reflux at 75 °C. The reaction was carried out under nitrogen. Upon completion, the reaction mixture was cooled to room temperature and treated as outlined in general method VII. The crude product was purified via flash column chromatography (eluent: dichloromethane/ethyl acetate, 2:1) to afford the desired product as a brown oil. Yield: 30% (0.104 g). IR: ν_max_ (ATR) cm^−1^: 2970, 2936, 2838, 1597, 1508, 1464, 1413, 1332, 1233, 1115, 1173, 1045, 997, 918, 833, 799, 691, 659. ^1^H NMR (400 MHz, CDCl_3_) δ 7.67 (s, 1 H, CH-N), 7.63 (s, 1 H, CH-N), 7.11 (s, 1 H, Ar-H), 6.96 (d, *J* = 8.5 Hz, 2 H, Ar-H), 6.88 (s, 2 H, Ar-H), 6.76 (s, 1 H, CH-N), 6.36 (s, 1 H, Ar-H), 5.77 (t, *J* = 7.4 Hz, 1 H, CH-N-R), 4.59 (dd, *J* = 7.1, 4.6 Hz, 1 H, CH), 3.82 (s, 3 H, OCH_3_), 3.80 (s, 3 H, OCH_3_), 3.76 (s, br, 3 H, OCH_3_), 3.20 (dt, *J* = 13.9, 8.5 Hz, 1 H, CH_2_), 2.62–2.58 (m, 1 H, CH_2_). ^13^C NMR (101 MHz, CDCl_3_) 155.46 (C), 154.55 (C), 150.13 (C), 143.18 (C), 135.93 (CH), 135.59 (C), 131.32 (CH), 128.07 (2×CH), 115.49 (2×CH), 102.40 (CH), 61.83 (CH), 60.80 (OCH_3_), 60.04 (OCH_3_), 56.19 (OCH_3_), 46.33 (CH), 45.66 (CH_2_) ppm. HRMS (EI): Found 367.1649 [M+H]^+^; C_21_H_23_N_2_O_4_ requires 367.1658.

**(1*E*,4*E*)-1,5-bis(3,4,5-trimethoxyphenyl)penta-1,4-dien-3-one (28):** 3,4,5-Trimethoxybenzaldehyde (2 equiv; 0.049 mol, 9.61 g) was dissolved in acetone (1 equiv; 0.245 mol, 1.8 mL). Half of this mixture was added to NaOH (10% aqueous) in H_2_O:EtOH (5:4; 90 mL) and left to stir for 15 min before the remainder of the aldehyde-ketone mixture was added and the mixture was stirred at 20 °C for a further 30 min. The resulting suspension was filtered and washed with water (3 × 100 mL) to remove any remaining NaOH. The crude product was then filtered, dried, and recrystallized from ethanol to afford the desired product as yellow crystals (68%), [127] Mp 135–138 °C. ^1^H NMR (400 MHz, CDCl_3_): δ 7.63 (d, *J* = 15.8 Hz, 2 H), 6.95 (d, *J* = 15.8 Hz, 2 H), 6.82 (s, 4 H), 3.89 (s, 12 H), 3.87 (s, 6 H). ^13^C NMR (101 MHz, CDCl_3_): 188.46, 153.44, 143.33, 140.39, 130.23, 124.74, 105.60, 60.97, 56.18 ppm.

**(1*E*,4*E*)-1,5-bis(3,4,5-trimethoxyphenyl)penta-1,4-dien-3-ol (29):** General method I was followed using (1*E*,4*E*)-1,5-bis(3,4,5-trimethoxyphenyl)penta-1,4-dien-3-one **28** (2.41 mmol, 1 g), NaBH_4_ (4 equiv) in MeOH (20 mL). The reaction mixture was stirred for 1 h. Purification by column chromatography afforded the desired product as an orange oil (92%). ^1^H NMR (400 MHz, CDCl_3_): δ 6.60 (s, 4 H), 6.55 (d, *J* = 15.6 Hz, 2 H), 6.19 (dd, *J* = 15.6, 6.4 Hz, 2 H), 4.95 (t, *J* = 6.4 Hz, 1 H), 3.84 (s, 12H), 3.82 (s, 6 H). ^13^C NMR (101 MHz, CDCl_3_): 153.29, 137.99, 132.21, 130.72, 129.90, 103.61, 73.44, 60.89, 56.06 ppm. LRMS (ESI): C_23_H_28_O_7_Na, found 439 [M+Na]^+^.

1-((1*E*,4*E*)-1,5-bis(3,4,5-trimethoxyphenyl)penta-1,4-dien-3-yl)-1*H*-imidazole (**30**):

General method III was followed using **29** (1 equiv; 0.12 mmol, 500 mg) and CDI (1,1′-carbonyldiimidazole) (1.3 eq) in dry acetonitrile (60 mL). The reaction mixture was stirred for 3 h at reflux, before purification using *n*-hexane:AcOEt:MeOH (4:6:1 gradient) to afford the desired product as an oil (27%). ^1^H NMR (400 MHz, CDCl_3_): δ 7.55 (s, 1 H), 7.09 (s, 1 H), 6.91 (s, 1 H), 6.73 (dd, *J* = 15.5, 10.0 Hz, 1 H), 6.59 (s, 2 H), 6.49 (d, *J* = 15.6 Hz, 1 H), 6.37 (s, 2 H), 6.19 (d, *J* = 10.1 Hz, 1 H), 6.13 (d, *J* = 6.5 Hz, 1 H), 5.76 (d, *J* = 6.5 Hz, 1 H), 3.84 (s, 6 H, 2×OCH_3_), 3.82 (s, 6 H, 2×OCH_3_), 3.79 (s, 6 H, 2×OCH_3_). ^13^C NMR (101 MHz, CDCl_3_): 153.61, 153.34, 134.76, 134.22, 132.20, 129.76, 126.42, 104.42, 103.61, 63.16, 60.83, 56.16 ppm. HRMS. Found: 467.2177, [M+H]^+^: C_26_H_31_N_2_O_6_ requires 467.2177.

(*E*)-3-(Anthracen-9-yl)-1-(4-iodophenyl)prop-2-en-1-ol (**32a**)

General method I was followed using **31a** (1.151 mmol, 500 mg), NaBH_4_ (4 equiv), MeOH (20 mL), and left to stir for 1 h to afford the desired product as a brown solid (78%), which was used in the following reaction without further purification. ^1^H NMR (400 MHz, CDCl_3_): δ 8.37 (s, 1H), 8.25–8.13 (m, 2 H), 8.00–7.93 (m, 2 H), 7.76 (d, *J* = 8.4 Hz, 2 H), 7.49–7.39 (m, 5 H), 7.33 (d, *J* = 8.4 Hz, 2 H), 6.21 (dd, *J* = 16.1, 6.2 Hz, 1 H), 5.66–5.57 (m, 1 H). ^13^C NMR (101 MHz, CDCl_3_): 142.43, 140.20, 139.52, 131.53, 131.35, 129.40, 128.68, 128.36, 126.93, 126.63, 125.58 (4C), 125.12, 93.42, 74.91 ppm.

(*E*)-3-(Anthracen-9-yl)-1-(pyridin-4-yl)prop-2-en-1-ol (**32b**)

General method I was followed using (*E*)-3-(anthracen-9-yl)-1-(pyridin-4-yl)prop-2-en-1-one (**31b**) (1.616 mmol, 500 mg), NaBH_4_ (2 equiv) in MeOH (30 mL). The reaction mixture was sonicated for 5 min and left to stir for 30 min to afford the desired product as an orange solid (98%). ^1^H NMR (400 MHz, DMSO-*d*_6_): δ 8.60 (d, *J* = 5.8 Hz, 2 H), 8.54 (s, 1 H), 8.25 (d, *J* = 5.0 Hz, 1 H), 8.24 (d, *J* = 9.9 Hz, 2 H), 8.08 (dd, *J* = 9.7 Hz, *J* = 5.1 Hz, 2 H), 7.58 (d, *J* = 5.9 Hz, 2 H), 7.51 (dd, *J* = 5.5, 4.3 Hz, 4 H), 6.16 (d, *J* = 4.6 Hz, 1 H), 6.10 (dd, *J* = 16.1, 6.4 Hz, 1 H), 5.61 (t, *J* = 5.3 Hz, 1 H). ^13^C NMR (101 MHz, DMSO-*d*_6_): 150.13, 141.14, 132.17, 131.41, 129.28, 129.06, 126.65, 126.22 (C), 125.90, 125.80, 125.70, 121.66, 72.70 ppm. LRMS (ESI): found 312 [M+H]^+^. HRMS; Found: 312.1388 [M+H]^+^; C_22_H_18_NO requires 312.1383.

(*E*)-1-(3-(Anthracen-9-yl)-1-(4-iodophenyl)allyl)-1*H*-imidazole (**33a**)

General method III was followed using **32a** (1 equiv; 0.458 mmol, 200 mg), and the reaction mixture was stirred for 3 h. Purification by column chromatography using *n*-hexane:AcOEt:MeOH (8:2:1 gradient) afforded the desired product **33a** as a yellow oil (58%). ^1^H NMR (400MHz, CDCl_3_): δ 8.54 (s, 1 H), 8.33 (d, J = 9.0 Hz, 1 H), 8.06 (d, *J* = 9.7 Hz, 4 H), 7.59 (d, *J* = 8.3 Hz, 1 H), 7.52 (d, *J* = 8.4 Hz, 1 H), 7.48 (d, *J* = 9.7 Hz, 4 H), 7.07 (d, *J* = 8.3 Hz, 2 H), 6.97 (d, *J* = 8.4 Hz, 1 H), 6.72 (d, *J* = 15.6 Hz, 1 H), 6.28 (d, *J* = 15.6 Hz, 1 H). ^13^C NMR (101 MHz, CDCl_3_): ppm 137.78, 137.45, 134.99, 134.65, 131.73, 130.50, 130.10, 129.84, 129.63, 128.75, 128.48, 127.84, 127.22, 126.35, 125.14, 124.66, 123.17, 60.37. HRMS: Found 487.0664 [M+H]^+^; C_26_H_20_IN_2_ requires 487.0666.

(*E*)-4-(3-(Anthracen-9-yl)-1-(1*H*-imidazol-1-yl)allyl)pyridine (**33b**)

General method E was followed using **32b** (1 equiv; 0.64 mmol, 200 mg) and the reaction mixture was stirred for 3 h to afford a black/red solution. Purification using *n*-hexane:AcOEt: MeOH (8:2:1 gradient) afforded the desired product **33b** as a brown/black oil (5%). ^1^H NMR (400 MHz, CDCl_3_): δ ppm 8.61 (s, 1 H), 8.29 (dd, *J* = 7.6, 2.0 Hz, 1 H), 7.83 (s, 1 H), 7.57–7.43 (m, 13 H), 7.38 (dd, *J* = 7.2, 1.8 Hz, 2 H), 7.25 (d, *J* = 16.1 Hz, 1 H), 6.19 (dt, *J* = 16.1, 6.8 Hz, 1 H). ^13^C NMR (101 MHz, CDCl_3_): 174.83, 149.33, 136.32, 135.02, 129.19, 128.49, 127.95, 126.01, 125.96, 125.28, 124.45, 124.24, 122.53, 60.37, 39.18, 21.09, 14.15. LRMS (ESI): found 362 [M+H]^+^ requires 362.1.

### 4.2. Biochemistry

#### 4.2.1. Materials

All the reagents and cell culture growth medium were purchased from BD Biosciences (Edmund Halley Road, Oxford, UK). Fluorescence for the AlamarBlue^®^ assay was read using the BMG-Labtech, FLUOstar Optima plate reader (Ortenberg, Germany) and the Gemini Spectramax plate reader (Molecular Devices, San Jose, CA, USA). All data points were analyzed using GraphPad PRISM (version 5) software (Graphpad Software Inc., San Diego, CA, USA). FACS analysis was carried out on BD Accuri (Beckman Coulter, BD Biosciences, San Jose, CA, USA) and FACSCalibur flow cytometer (BD Biosciences, San Jose, CA, USA) using the CellQuest Software (Becton-Dickinson, San Jose, CA, USA). The substrate DBF (dibenzylfluorescein) was obtained from Gentest Corporation (Woburn, MA, USA). All human recombinant cytochrome P450 enzymes were purchased from BD Biosciences, San Jose, CA. Human promyelocytic leukemia (HL-60) cells were purchased from American Type Culture Collection (ATCC) Manassas, VA, USA, and originally obtained from a Caucasian female with acute promyelocytic leukemia. HL-60 cells were cultured in Roswell Park Memorial Institute Media (RPMI-1640) with GlutaMAX™ completed with FBS (10%) and penicillin/streptomycin (1%). Human breast adenocarcinoma cell line (MCF-7) was purchased from the American Type Culture Collection (ATCC) Manassas, VA, USA. Normal breast cells (MCF-10A) (adherent) were obtained as a kind gift from Dr. Susan McDonnell, UCD School of Chemical and Bioprocess Engineering. Invasive ductal carcinomal cells (MDA-MB-231) were purchased from the American Type Culture Collection (ATCC) Manassas, VA, USA.

#### 4.2.2. Cell Culture

HL-60 cells were suspension cells and the seeding density for the viability assay was 25,000 cells/mL. MCF-7 cells (adherent) were cultured in Minimum Essential Media (MEM) with GlutaMAX™-I, supplemented with 1% (*v*/*v*) non-essential amino acids, 10% (*v*/*v*) fetal bovine serum (FBS), purchased from BD Biosciences (Edmund Halley Road, Oxford, UK), and 1% (*v*/*v*) penicillin/streptomycin 5000 U/mL. The MCF-7 cells used in the screening of the compounds during these experiments were mycoplasma-free. The seeding density of MCF-7 in the viability assay was 25,000 cells/mL and 50,000 cells/mL in the FACS assay. Normal breast cells (MCF-10A) (adherent) were cultured in Dulbecco’s Modified Eagle Medium: Nutrient Mixture F-12 (DMEM/F12; Gibco) supplemented with 5% horse serum (Invitrogen, Waltham, MA, USA), 20 ng/mL epidermal growth factor (Merck Millipore, Burlington, MA, USA), 0.5 μg/mL hydrocortisone (Sigma, Kanagawa, Japan), 100 ng/mL cholera toxin (Sigma), 10 μg/mL insulin (Sigma), and penicillin/streptomycin 5000 U/mL (1%) (Gibco, Waltham, MA, USA). Invasive ductal carcinoma cells (MDA-MB-231) (adherent cells) are metastatic triple-negative breast cancer cells and do not express the estrogen receptor, progesterone receptor, or the HER2 receptor. MDA-MB-231 cells were cultured in Dulbecco’s Modified Eagle Medium, which was supplemented with 10% (*v*/*v*) fetal bovine serum (FBS) and 1% (*v*/*v*) penicillin/streptomycin 5000 U/mL. When not in use, all cells were kept in liquid nitrogen and frozen in a freezing media made of 90% FBS and 10% DMSO. All cells were grown in an atmosphere of 5% CO_2_/95% air in T75 culture flasks. The media was changed every 2–3 days and media was always prewarmed to 37 °C. All were sub-cultured every 3–4 days by trypsinization using TrypLE™ Express enzyme when confluence was reached to allow growth, prevent excessive cell death, and minimize the risk of infections derived by over-confluence. For the cell viability assay, the number of cells per milliliter was 25,000 cells/mL while the FACS assay utilized 50,000 cells/mL. Cells were maintained at 37 °C in 5% CO_2_ in a humidified incubator.

#### 4.2.3. Cell Viability Assay (AlamarBlue)

The biochemical assays were performed in triplicate and on at least three independent occasions to facilitate the determination of mean values. For the viability assay, cells were grown until 80% confluent. Adherent cells such as MCF-7, MDA-MB-231, and MCF-10A were trypsinized to detach them from the flask, counted as previously described, and seeded in 96 well plates with a seeding density of 25,000 cells /mL (200 µL of suspension in each well so that the final number of cells per well was 5000) and 1 × 10^4^ cells/well seeding density for suspension HL-60 cells. Adherent cells were incubated for 24 h after being seeded in the 96 well plate and treated on the following day, while suspension cells were treated on the same day of the seeding. In both cases, the incubation time after the treatment with the drug was 72 h. After 68 h incubation, the AlamarBlue was added (20 µL in each well) and the incubation time was completed. After 72 h, the change in color was measured by spectrofluorimetry at an excitation wavelength of 544 nm and emission wavelength of 590 nm. AlamarBlue is a cell viability indicator containing a compound called resazurin. AlamarBlue is water soluble, stable in culture media, non-toxic, and is permeable through the cell membrane. When resazurin (blue) enters the live cells, it is reduced to the fluorescent molecule resorufin (pink).

#### 4.2.4. Cell Cycle Analysis: Flow Cytometry

Cells (MCF-7 and MDA-MD 231) were seeded at a density of 1 × 10^5^ cells/well in 6-well plates (volume of 3 mL per well) and treated with selected compound **22b** and phenstatin (**19c**) (1 μM), as previously reported [65]. The time points used were 24, 48, or 72 h. Ethanol was used as the vehicle. At each time point, the media was removed and then the well was carefully rinsed with PBS and TrypLE™ Express enzyme (200 µL) was added to detach the adherent cells. The cells were collected by trypsinization and were then centrifuged at 800× *g* for 15 min. Cells were washed with ice-cold phosphate-buffered saline (PBS) ×2 and fixed in ice-cold 70% ethanol for 14 h at −20 °C. Fixed cells were centrifuged at 800× *g* for 15 min. The samples were then treated with 12.5 µL of DNase-free RNAse A (10 mg/mL) together with 37.5 mL of PI (1 mg/mL) at 37 °C for 30 min, vortexed, and wrapped in tin foil. The DNA content of cells (10,000 cells/selected experimental group) was determined by flow cytometry at 488 nm with a FACSCalibur flow cytometer (BD Biosciences, San Jose, CA, USA) using the CellQuest Software (Becton-Dickinson, East Rutherford, NJ, USA). Each experiment was performed on three separate occasions.

#### 4.2.5. Annexin V/PI Apoptotic Assay

Apoptotic cell death was monitored by flow cytometry using Annexin V and propidium iodide (PI) to determine the Annexin V and PI negative cells (Q4, healthy cells), Annexin V positive and PI negative cells (Q3, early apoptosis), Annexin V and PI positive cells (Q2, late apoptosis), and Annexin V negative and PI-positive cells (Q1, necrosis) cells. MCF-7 and MDA-MB-231 cells for this experiment were seeded in 6-well plates at a density of 1 × 10^5^ cells/mL (3 mL). Following the protocol previously described [65], the cells were treated at 37 °C with either vehicle (0.1% (*v*/*v*) EtOH), phenstatin **19c**, (0.1 μM and 0.5 μM), or **22b** (0.1 μM, 0.5 μM and 1 μM) at the 48 h time point. Cells were harvested by centrifugation at 400× *g* using a temperature-controlled Sorvall centrifuge and then prepared for flow cytometric analysis. Cells were washed in Annexin V Binding Buffer 1X (binding buffer: 0.1 M 4-(2-hydroxyethyl)-1-piperazineethanesulfonic acid (HEPES), pH 7.4; 1.4 M NaCl; 25 mM CaCl_2_ diluted in dH_2_O, 0.5 mL), and incubated in the dark for 30 min on ice in Annexin V-containing binding buffer (1:100), 50 μL protected from light. Cells were then washed once in binding buffer and then re-suspended in a PI-containing binding buffer (1:1,000) (0.5 μg/mL, 500 μL) and immediately analyzed within 1 h to determine the populations produced. BD Accuri flow cytometer (BD Biosciences, 2350 Qume Dr, San Jose, CA, USA) and GraphPad Prism software were used for the analysis of the data (GraphPad Software, Inc., 2365 Northside Dr., Suite 560, San Diego, CA, USA).

#### 4.2.6. Immunofluorescence Microscopy

The effects of treatment with compound **22b** on the MCF-7 cytoskeleton were demonstrated using confocal microscopy following the protocol previously described [65]. Briefly, the MCF-7 cells were seeded at a density of 1 × 10^5^ cells/mL on eight chamber glass slides (BD Biosciences). The cells were then treated with vehicle (1% ethanol (*v*/*v*)), paclitaxel (1 μM), phenstatin (1 μM), compound **22b** (10 μM) for 16 h. The cells were then washed in PBS, fixed for 20 min with 4% paraformaldehyde in PBS, and permeabilized in 0.5% Triton X-100. The cells were washed in PBS containing 0.1% Tween (PBST) and blocked using 5% bovine serum albumin diluted in PBST ((phosphate-buffered saline with Tween 20). Cells were incubated with mouse monoclonal anti-tubulin-FITC antibody (clone DM1A) (Sigma) (1:100) for 2 h at room temperature. Following washes in PBS with Tween^®^20 (PBST), cells were incubated with Alexa Fluor 488 dye (1:500) for 1 h at room temperature. Following washing in PBST, the cells were mounted in Ultra Cruz Mounting Media (Santa Cruz Biotechnology, Santa Cruz, CA, USA) containing 4,6-diamino-2-phenolindol dihydrochloride (DAPI). Images of the cells were obtained using Leica SP8 confocal microscopy (Wetzlar, Germany) with Leica application suite X software (Wetzlar, Germany). Experiments were performed on three independent occasions.

#### 4.2.7. Tubulin Polymerization Assay

Paclitaxel was used as a control in the tubulin polymerization assay, which stabilizes tubulin in the polymerized form. The triazole **22b** was selected for evaluation in the tubulin polymerization assay. Following the protocol previously described [65], the polymerization of purified bovine tubulin was monitored using a tubulin polymerization assay kit, BK006, (Cytoskeleton Inc., Denver, CO, USA). The assay was carried out using the purified bovine brain tubulin. Tubulin polymerization was determined spectrophotometrically by monitoring the change in turbidity since light is scattered proportionally to the concentration of polymerized microtubules in the assay. Lyophilized tubulin [128] (Cytoskeleton, Denver, CO, USA) (>99%, 3 mg/mL) was re-suspended in ice-cold G-PEM buffer (80 mM PIPES pH 6.9, 0.5 mM MgCl_2_, 1 mM EGTA, 1 mM GTP, 10.2% (*v*/*v*) glycerol) and added to wells on a half volume 96-well plate containing the designated concentration of drug (10 or 30 μM). The tubulin was incubated at 37 °C in the presence of either vehicle (1% DMSO (*v*/*v*) ddH_2_O), paclitaxel (10 μM), phenstatin **19c** (10 μM), or triazole **22b** (10 μM and 30 μM). Samples were mixed well and the tubulin assembly was monitored at 340 nm at 30 s intervals for 60 min at 37 °C in a Spectramax 340PC spectrophotometer (Molecular Devices, San Jose, CA, USA).

#### 4.2.8. Cytochrome P450 Assays (CYP19 (Aromatase) and CYP1A1)

The human recombinant cytochrome P450 enzymes were purchased from BD Biosciences, San Jose, CA and the dibenzylfluorescein (DBF) substrate was purchased from Gentest Corporation (Woburn, MA, USA). Aromatase and CYP1A1 inhibition were quantified by monitoring the fluorescent intensity of fluorescein, which is the hydrolysis product of DBF by aromatase, as previously described [65,112,113]. Compound **22b** (10 μL) was pre-incubated with the NADPH regenerating system (90 μL of 2.6 mM NADP^+^, 7.6 mM glucose 6-phosphate, 0.8 U/mL glucose 6-phosphate dehydrogenase, 13.9 mM MgCl_2_, and 1 mg/mL albumin in 50 mM potassium phosphate, pH 7.4), for 10 min, at 37 °C, before 100 μL of the enzyme and substrate (E/S) mixture were added (4.0 pmol/well of CYP19/0.4 μM DBF; 5.0 pmol/well of CYP2C8/2.0 μM DBF; 5.0 pmol/well of CYP3A4/2.0 μM DBF and 0.5 pmol/well of CYP1A1/2.0 μM DBF). The reaction mixtures were incubated for 30 min (for CYP1A1, 25 min incubation) at 37 °C for generation of product. The reaction was quenched with 2 N NaOH (75 μL), shaken for 5 min, and incubated for 2 h at 37 °C. Fluorescence was measured at 485 nm (excitation) and 530 nm (emission). Three independent experiments were performed, each one in triplicate, with the average values used to construct dose–response curves. At least four concentrations of the test substance were used, and the IC_50_ value was calculated (*TablecurveTM2D,* AISN Software, EUA, 1996). Naringenin was used as a positive control, giving an IC_50_ value of 4.9 μM. Compound **22b** was dissolved in dimethyl sulfoxide (DMSO) and diluted to final concentrations. An equivalent volume of DMSO was added to control wells, and this had no measurable effect on cultured cells or enzymes. Compounds were considered for further experiments when showing inhibition greater than 90%.

#### 4.2.9. Computational Study: Molecular Docking

Docking calculations using Molecular Operating Environment (MOE) version 2022.02 [117] were undertaken on (*E*)-5-(3-(1*H*-1,2,4-triazol-1-yl)-3-(3,4,5-trimethoxyphenyl)prop-1-en-1-yl)-2-methoxyphenol (**22b**), *R* and *S* enantiomers. The 1SA0 X-ray structure of bovine tubulin co-crystallized with *N*-deacetyl-*N*-(2-mercaptoacetyl)colchicine (DAMA-colchicine) was used for the docking study and was downloaded from the PDB website [116]. Using a UniProt Align analysis, 100% sequence identity between human and bovine β tubulin was confirmed. The crystal structure was prepared using QuickPrep (minimized to a gradient of 0.001 kcal/mol/Å), Protonate 3D, Residue pKa, and Partial Charges protocols in MOE 2015 with the MMFF94x force field. For the docking study, compounds *(S)-***22b** and *(R)-***22b** were drawn in MOE, saved as mdb files, and processed in MOE. For each compound, MMFF94x partial charges were calculated and each was minimized to a gradient of 0.001 kcal/mol/Å. Default parameters were used for the docking study; however, 300 poses were sampled for each compound and the top 50 docked poses were retained for subsequent analysis.

## 5. Conclusions

Breast cancer is recognized as one of the leading causes of cancer-related deaths worldwide; hormone-dependent BC is the most common in post-menopausal women. The cancer drug development failure rate for small molecules is estimated to be in the region of 95%, although significant improvements in all aspects of the drug development process have been achieved in recent years [129,130]. Despite therapeutic advances, there is still a need for more precise and effective therapies [131,132,133,134]. The clinically used antimitotic drugs vinca alkaloids, epothilones, and taxanes are very effective anti-cancer therapeutics in the treatment of leukemias, lymphomas, ovarian, prostate, and triple-negative BC. However, resistance to anti-microtubule cancer drugs and dose-limiting side effects are significant clinical issues for these cancer drugs [135,136,137]. Triazole-containing hybrid compounds have a wide range of biological activities and many novel aromatase inhibitors based on 1,2,4-triazole and 1,2,3-triazole are reported [138,139]. While triazole-containing antimitotic molecules have been reported [140], we wished to identify possible dual aromatase-tubulin targeting compounds. Aromatase inhibitors are now the first-line treatment for hormone-dependent BC in postmenopausal women [12].

In the present work, the synthesis of phenstatin-letrozole hybrid compounds is now extended to include heterocyclic modifications of chalcones with the synthesis novel hybrid (*E*)-1-(1,3-diphenylallyl)-1*H*-1,2,4-triazoles and related compounds as dual aromatase-tubulin targeting compounds with activity in breast cancer. The objective of this strategy was the development of novel tubulin inhibitors in breast cancer cells with potential dual-targeting of tubulin and aromatase. The tubulin-targeting pharmacophore is contained in the chalcone-derived structure, while the aromatase-targeting activity is associated with the triazole. Drug resistance is a major challenge in conventional endocrine therapy for estrogen receptor-positive breast cancer. In this approach, simultaneous aromatase inhibition and tubulin polymerization inhibition by the hybrid compound may effectively block multiple oncogenic pathways and overcome resistance.

A preliminary evaluation of the novel compounds in ER+/PR+MCF-7 breast cancer cells identified compound **22b** as a potent antiproliferative compound (IC_50_ = 0.385 μM) in MCF-7 breast cancer cells (ER+/PR+) and 0.765 μM in triple-negative MDA-MB-231 breast cancer cells. Compound **22b** also demonstrated sub-micromolar activity over the NCI panel of 60 cancer cell lines including prostate, melanoma, colon, leukemia, and non-small cell lung cancers. The antimitotic action of compound **22b** was confirmed with G_2_/M phase cell cycle arrest, induction of apoptosis in MCF-7 cells, and inhibition of tubulin polymerization. Compound **22b** targeted tubulin and induced multinucleation in MCF-7 cells. Furthermore, the antiproliferative activity of the lead compound was demonstrated to be selective for cancer cells, as the compound did not show significant effects on MCF-10A normal breast cells. Computational docking studies were used to illustrate the potential binding conformations of **22b** in the colchicine binding site of tubulin. In addition, compound **22b** also selectively inhibited aromatase (CYP19). The structural modification developed in this work by the introduction of the heterocycle 1,2,4-triazole on the chalcones scaffold structure has identified lead compounds that exhibit promising anti-proliferative properties as tubulin targeting agents and aromatase inhibitors, which have potential application in the treatment of BC. Future developments will include the resolution of the enantiomers of the lead triazole compound **22b** and the determination of the selective potency of these enantiomers in breast and colon cancer cell lines. These novel compounds are identified as potential candidates for further investigation as antiproliferative microtubule-targeting agents for breast cancer and offer the potential for further development of this novel class of compounds.

## Figures and Tables

**Table 1:** Antitumor evaluation of compounds **22a**, **22b**, **23b, 27a**, and **30** (growth percent) in the NCI60 cell line in vitro one dose primary screen ^a,b^.

Cell line	22a	22b	23b	27a	30	Cell line	22a	22b	23b	27a	30
**Leukemia**						**Melanoma**					
CCRF-CEM	35.07	6.31	37.13	90.92	65.10	LOX IMVI	49.98	50.87	46.09	86.38	85.14
HL-60 (TB)	−17.94	−36.28	−36.59	83.57	70.60	MALME-3M	61.34	54.35	57.72	73.18	93.80
K-562	17.72	8.05	13.63	44.70	61.41	M14	17.86	−12.29	18.61	73.08	82.03
MOLT-4	39.43	21.84	31.88	90.04	74.07	MDA-MB-435	−9.62	−32.99	−25.56	5.00	45.63
RPMI-8226	50.84	19.89	15.14	94.65	60.48	SK-MEL-2	12.89	18.23	41.65	89.63	81.02
SR	18.93	5.67	6.18	35.42	31.50	SK-MEL-28	62.59	59.08	58.86	78.29	100.52
**Non-Small Cell Lung Cancer**						SK-MEL-5	33.03	23.87	16.98	53.15	62.09
A549/ATCC	35.48	40.13	29.07	55.83	78.93	UACC-257	46.44	55.61	34.19	58.46	75.61
EKVX	66.04	59.45	81.22	100.83	94.43	UACC-62	33.84	37.50	36.37	65.53	68.23
HOP-62	41.84	22.52	47.25	80.77	84.91	**Ovarian Cancer**					
HOP-92	40.32	47.98	30.49	59.58	56.24	IGROV1	64.61	50.48	55.74	82.43	78.02
NCI-H226	75.97	62.21	88.36	97.37	84.81	OVCAR-3	9.06	15.65	4.86	57.06	83.41
NCI-H23	67.01	41.50	55.69	91.50	81.16	OVCAR-4	96.82	83.51	78.81	100.34	92.69
NCI-H322M	61.24	54.65	45.84	101.51	96.68	OVCAR-5	90.16	86.55	102.8	106.81	129.83
NCI-H460	22.14	13.26	16.55	78.74	93.96	OVCAR-8	52.16	15.99	40.97	89.25	83.74
NCI-H522	28.37	13.04	24.65	50.13	64.75	NCI/ADR-RES	36.28	10.82	45.70	93.87	89.35
**Colon Cancer**						SK-OV-3	35.55	12.67	33.54	86.31	92.06
COLO 205	30.72	60.64	89.81	106.99	99.42	**Renal Cancer**					
HCC-2998	84.15	67.70	76.97	99.22	99.87	786-0	39.65	23.33	34.04	91.67	95.20
HCT-116	21.99	10.52	16.67	81.13	78.18	A498	36.38	19.81	67.46	88.34	104.55
HCT-15	31.14	16.53	24.91	59.92	77.23	ACHN	57.45	63.11	46.34	86.36	99.92
HT29	4.96	41.01	70.97	101.76	80.30	CAKI-1	51.61	34.50	60.21	74.50	nd
KM12	30.66	26.67	29.04	50.59	82.35	RXF 393	46.51	−16.94	33.07	70.77	88.15
SW-620	26.63	27.62	17.41	62.47	88.00	SN12C	62.02	49.61	57.15	104.36	84.52
**CNS Cancer**						TK-10	48.52	41.24	84.45	101.23	115.50
SF-268	57.07	54.09	56.10	89.96	91.93	UO-31	64.39	54.20	59.92	79.93	66.56
SF-295	22.52	1.43	16.59	57.51	95.73	**Prostate Cancer**					
SF-539	56.52	−4.58	23.09	89.80	93.55	PC-3	31.20	23.47	25.28	60.69	58.09
SNB-19	49.01	34.19	47.27	93.73	89.08	DU-145	61.25	21.00	37.76	102.37	100.17
SNB-75	51.27	40.99	20.04	90.16	54.99	**Breast Cancer**					
U251	31.77	20.72	27.12	80.36	81.64	MCF7	22.48	21.22	17.87	44.77	57.42
						MDA-MB-231/ATCC	50.05	45.54	52.97	81.01	76.21
						HS 578T	51.18	28.47	32.05	85.38	77.21
						BT-549	45.45	36.59	44.34	90.36	83.42
						T-47D	30.79	34.38	40.62	94.34	77.54
						MDA-MB-468	40.88	8.27	40.60	78.64	57.22

^a^ NCI in vitro human tumor cell screen 1 dose assay for compounds **22a** (NCI 788808), **22b** (NCI 788807), **23b** (NCI 788810), **27a** (NCI 788809), and **30** (NCI 792964). The compounds were evaluated at 10 µM concentration over the NCI 60 cell line panel, and incubations were carried out over 48 h exposures to the drug. ^b^ Mean growth percent 41.89%, 29.92%, 39.73%, 79.21%, and 81.29% for compounds **22a** (788808), **22b** (788807), **23b** (788810), **27a** (788809), and **30** (792964), respectively.

**Table 2:** Antiproliferative evaluation of compound **22b** against the NCI-60 cell line in vitro screen.

Cell Line	Compound 22b	Cell Line	Compound 22b
	GI_50_ (μM) ^b,c^		GI_50_ (μM) ^b,c^
**Leukemia**		**Colon Cancer**	
CCRF-CEM	0.0320	COLO 205	3.66
HL-60(TB)	0.0245	HCC-2998	3.00
K-562	0.0320	HCT-116	0.0410
MOLT-4	nd ^d^	HCT-15	0.0508
RPMI-8226	0.0370	HT29	3.68
SR	0.0248	KM12	0.0405
**Melanoma**		SW-620	0.0394
LOX IMVI	0.0511	**Renal Cancer**	
MALME-3M	>100 *	786-0	−5.05 *
M14	0.0287	A498	3.13
MDA-MB-435	0.0183	ACHN	0.0579
SK-MEL-2	0.0438	CAKI-1	2.83
SK-MEL-28	3.62	RXF 393	>100 *
SK-MEL-5	0.0458	SN12C	7.19
UACC-257	>100	TK-10	21.2
UACC-62	0.0476	UO-31	>100 *
**Lung Cancer**		**CNS Cancer**	
A549/ATCC	4.56	SF-268	0.0589
EKVX	6.07	SF-295	−5.43 *
HOP-62	−4.52 *	SF-539	0.0267
HOP-92	67.0	SNB-19	−4.59 *
NCI-H226	5.93	SNB-75	>100 *
NCI-H23	3.47	U251	0.0506
NCI-H322M	>100 *	**Prostate Cancer**	
NCI-H460	0.0423	PC-3	0.0360
NCI-H522	0.0223	DU-145	−5.18 *
**Ovarian Cancer**		**Breast Cancer**	
IGROV1	5.83	MCF7	0.0330
OVCAR-3	0.0260	MDA-MB-231/ATCC	3.02
OVCAR-4	7.05	HS 578T	>100 *
OVCAR-5	4.48	BT-549	0.0712
OVCAR-8	−5.44 *	T-47D	>100
NCI/ADR-RES	−5.28 *	MDA-MB-468	>100 *
SK-OV-3	>100 *	**MG-MID** ^e^	0.3715

^b^ GI_50_ is the molar concentration of the compound causing 50% inhibition of growth of the tumor cells; ^c^ NSC 788807; ^d^ Nd: Not determined; ^e^ MG-MID: the mean of GI_50_ values over all cell lines for the tested compound. * IC_50_ values.

## Data Availability

Data are contained within the article or supplementary material.

## Supplementary Materials

The following supporting information can be downloaded at https://www.mdpi.com/article/10.3390/ph18010118/s1: Experimental details for the preparation of chalcones **20a–h**, **20j**, **31a–c**, 1,3-diarylprop-2-en-1-ols **21a–c**; Bioavailability analysis for compounds **22a**, **22b**, **23a**, and **23b**; The BOILED-Egg evaluation of passive gastrointestinal absorption (HIA) and brain penetration (BBB) of compounds **22a**, **22b**, **23a** and **23b**; ^1^H NMR and ^13^C NMR spectra for (*E*)-1-(3-(4-methoxyphenyl)-1-(3,4,5-trimethoxyphenyl)allyl)-1*H*-1,2,4-triazoles and related compounds; Tier-1 profiling screen, physicochemical descriptors, Lipinski properties pharmacokinetic, ADMET and drug-likeness predictions for (*E*)-1-(3-(4-methoxyphenyl)-1-(3,4,5-trimethoxyphenyl)allyl)-1*H*-1,2,4-triazoles and related compounds; Overlay of imidazole-chalcones with letrozole and phenstatin; COMPARE analysis for compound **22b**.

## Abbreviations

The following abbreviations are used in this manuscript:

AI	Aromatase inhibitor
ADC	Antibody–drug conjugate
ATR	Attenuated total reflection
BC	Breast Cancer
CDI	1,1′-Carbonyldiimidazole
CTD	C-Terminal domain
CYP19	Cytochrome P450 family
DEPT	Distortionless Enhancement by Polarization Transfer
DMEM	Dulbecco’s Modified Eagle Medium
DMSO	Dimethyl sulfoxide
ECACC	European Collection of Animal Cell Cultures
EGFR	Epidermal growth factor receptor
ER	Estrogen receptor
FACS	Fluorescence activated cell sorting
FBS	Fetal bovine serum
GI50	50% Growth inhibitory concentration
HER2	Human epidermal growth factor receptor 2
HER/neu	Receptor tyrosine-protein kinase erbB-2, CD340
HDBC	Hormone-dependent breast cancer
HR	Hormone receptor
LC50	Median lethal concentration
MBC	Metastatic breast cancer
MDR	Multidrug resistance
MEM	Minimum essential media
NCI	National Cancer Institute
NMR	Nuclear magnetic resonance
PARP	Poly (ADP-ribose) polymerase
PBS	Phosphate buffered saline
PBST	PBS containing 0.1% Tween
PI	Propidium iodide
PIK3CA	Phosphatidylinositol-4,5-Bisphosphate 3-Kinase Catalytic Subunit Alpha
PR	Progesterone receptor
PROTAC	Proteolysis targeting chimeric
SERCA	Selective estrogen receptor covalent antagonist
SERM	Selective estrogen receptor modulator
SERD	selective estrogen receptor degraders (SERD)
STS	Steroid sulfatase
TGI	Total growth inhibitory concentration
TLC	Thin layer chromatography
TNBC	Triple-negative breast cancer
