# Supplementary material for: (E)-1-(3-(3-Hydroxy-4-Methoxyphenyl)-1-(3,4,5-Trimethoxyphenyl)allyl)-1H-1,2,4-Triazole and Related Compounds: Their Synthesis and Biological Evaluation as Novel Antimitotic Agents Targeting Breast Cancer
Source: Pharmaceuticals (Basel). 2025 Jan 17;18(1):118. doi: 10.3390/ph18010118 (PMC11769294; doi:10.3390/ph18010118)
Supplement: Supplementary file 1 [file pharmaceuticals-18-00118-s001.zip › pharmaceuticals-3361293-supplementary.pdf]

Supplementary Information

(*E*)-1-(3-(3-Hydroxy-4-Methoxyphenyl)-1-(3,4,5-Trimethoxyphenyl)allyl)-1*H*-1,2,4-Triazole and Related Compounds: Their Synthesis and Biological Evaluation as Novel Antimitotic Agents Targeting Breast Cancer

Gloria Ana[1], Azizah M. Malebari[2], Sara Noorani[1], Darren Fayne[3,4], Niamh M. O'Boyle[1], Daniela M Zisterer[5], Elisangela Flavia Pimentel[6], Denise Coutinho Endringer[6], Mary J. Meegan[1*]

[1] School of Pharmacy and Pharmaceutical Sciences, Panoz Institute, Trinity College Dublin, D02 PN40 Dublin, Ireland.

[2] Department of Pharmaceutical Chemistry, College of Pharmacy, King Abdulaziz University, Jeddah 21589, Saudi Arabia.

[3] Molecular Design Group, School of Chemical Sciences, Dublin City University, Glasnevin, D09 V209 Dublin, Ireland.

[4] DCU Life Sciences Institute, Dublin City University, Glasnevin, D09 V209 Dublin, Ireland.

[5] School of Biochemistry and Immunology, Trinity Biomedical Sciences Institute, Trinity College Dublin, 152-160 Pearse Street, D02R590 Dublin, Ireland.

[6] Department of Pharmaceutical Sciences, University Vila Velha, Av. Comissário José Dantas de Melo, n°21, Boa Vista, Vila Velha CEP 29102-920, Brazil.

* Correspondence: mmeegan@tcd.ie Tel.:+353-1-896-2798

Supplementary Information

Experimental Chemistry

Figure S1: Bioavailability Radar for **22a, 22b, 23a** and **23b**.

Figure S2: The BOILED-Egg evaluation of passive gastrointestinal absorption (HIA) and brain penetration (BBB) of compound **22a, 22b, 23a** and **23b** as function of the position of the molecule in the WLOGP-*versus*-TPSA map.

Figure S3-S18: ^{1}H-NMR and ^{13}C NMR spectra

Figure S19: Overlay of Imidazole-chalcones with phenstatin, letrozole and phenstatin

Table S1: Standard COMPARE Analysis of compound **22b**

Table S2: Physicochemical descriptors of selected (*E*)-1-(3-(4-methoxyphenyl)-1-(3,4,5-trimethoxyphenyl)allyl)-1*H*-1,2,4-triazoles and related compounds.

Table S3: Pharmacokinetic interaction estimations with P-gp and CYP 450 isozymes of selected compounds.

Table S4: Drug-likeness of representative compounds assessed via lead-likeness and reactivity/toxicity filters for selected compounds.

References

Materials and methods: Chemistry

Melting points were measured on a Gallenkamp SMP 11 melting point apparatus and are uncorrected. Infra-red (IR) spectra were recorded as thin film on NaCl plates, or as potassium bromide discs on a Perkin Elmer FT-IR Spectum 100 spectrometer. ^{1}H and ^{13}C nuclear magnetic resonance (NMR) spectra were recorded at 27°C on a Bruker Avance DPX 400 spectrometer (400.13 MHz, ^{1}H; 100.61 MHz, ^{13}C) at 20 °C in $CDCl_3$ (internal standard tetramethylsilane TMS) or DMSO-d_6. For $CDCl_3$, ^{1}H-NMR spectra were assigned relative to the TMS peak at 0.00 δ and ^{13}C-NMR spectra were assigned relative to the middle $CDCl_3$ triplet at 77.00 ppm. Electrospray ionisation mass spectrometry (ESI-MS) on a liquid chromatography time-of-flight (TOF) mass spectrometer (Micromass LCT, Waters Ltd., Manchester, UK) equipped with electrospray ionization (ES) interface operated in the positive ion mode with High Resolution Mass measurement accuracies of < ±5 ppm. R_f values are quoted for thin layer chromatography on silica gel Merck F-254 plates. Flash column chromatography was carried out on Merck Kieselgel 60 (particle size 0.040-0.063 mm) and also on Biotage SP4 instrument. All products isolated were homogenous on TLC. Analytical high-performance liquid chromatography (HPLC) for purity determination of products was performed using a Waters 2487 Dual Wavelength Absorbance detector, Waters 1525 binary HPLC pump, Waters In-Line Degasser AF and Waters 717plus Autosampler and Varian Pursuit XRs C18 reverse phase 150 x 4.6 mm chromatography column with detection at 254 nm.

General method I: Preparation of chalcones 20a-h, 20j.

To a solution of the appropriate aryl aldehyde (1 eq) in methanol (15-20 mL) containing KOH (50%) was added 1-(3,4,5-trimethoxyphenyl)ethan-1-one (1 eq) while stirring at ambient temperature for 24 h. After 24 h, water and HCl dil. were added to complete the precipitation. The solid product was then filtered and the product collected and recrystallised from methanol to afford the desired chalcone.

(*E*)-3-(4-Methoxyphenyl)-1-(3,4,5-trimethoxyphenyl)prop-2-en-1-one (20a). As per general method I, 3,4,5-trimethoxyacetophenone (1 eq, 4.7 mmol, 1 g) was reacted with 4-methoxybenzaldehyde (1 eq, 4.7 mmol, 0.64 g) and KOH 50% (10 mL) in methanol at RT for 24 h. The precipitate was filtered and recrystallised from methanol to afford the product, yield 67%, 1.04 g, white crystals, Mp. 96-100 °C [1]. IR: ν_{max} (ATR) cm^{-1}: 3227, 3005, 1654, 1591, 1579, 1457, 1408, 1318, 1245, 1155, 1120, 997, 973, 823, 664, 558. ^{1}H NMR (400 MHz, $CDCl_3$) δ 3.85 (s, 3 H, OCH_3), 3.92 (s, 3 H, OCH_3), 3.93 (s, 6 H, 2×OCH_3), 6.93 (d, *J*=8.71 Hz, 2 H, Ar-H), 7.25 (s, 2 H, Ar-H), 7.34 (d, *J*=15.34 Hz, 1 H, C<u>H</u>=CH), 7.60 (d,

J=8.71 Hz, 2 H, Ar-H), 7.78 (d, J=15.34 Hz, 1 H, CH=C<u>H</u>). ^{13}C NMR (101 MHz, $CDCl_3$) 55.41 (OCH_3), 56.38 (2×OCH_3), 60.96 (OCH), 105.99 (2×CH), 114.42 (2×CH), 119.42 (<u>C</u>H=CH), 127.60 (C), 130.21 (2×CH), 133.82 (C), 144.59 (C-O, CH=<u>C</u>H), 153.11 (2×C-O), 161.68 (C-O), 189.25 (C=O) ppm. HRMS (EI): Found 329.1374 $(M+H)^+$; $C_{19}H_{21}O_5$ requires 329.1389.

(*E*)-3-(3-Hydroxy-4-methoxyphenyl)-1-(3,4,5-trimethoxyphenyl)prop-2-en-1-one (20b). As per general method I, 3,4,5-trimethoxyacetophenone (1 eq, 7.13 mmol, 1.5 g) was reacted with 3-hydroxy-4-methoxybenzaldehyde (1 eq, 7.13 mmol, 1.08 g) and KOH 50% (10 mL) in methanol at RT for 24 h. The precipitate was filtered and recrystallised from methanol to afford the product, yield: 73%, 1.8 g, orange solid, Mp. 137-141 °C [2]. IR: ν_{max} (ATR) cm^{-1}: 3293, 2946, 2838, 1650, 1571, 1504, 1449, 1412, 1311, 1267, 1226, 1154, 1029, 976, 843, 745, 665. ^{1}H NMR (400 MHz, DMSO-d_6) δ 3.73 (s, 3 H, OCH_3), 3.81 (s, 3 H, OCH_3), 3.87 (s, 6 H, 2×OCH_3), 6.97 (d, J=8.29 Hz, 1 H, Ar-H), 7.27 (dd, J=8.29, 2.07 Hz, 1 H, Ar-H), 7.34 (d, J=2.07 Hz, 1 H, Ar-H), 7.37 (s, 2 H, Ar-H), 7.57 - 7.62 (m, 1 H, C<u>H</u>=CH), 7.65 - 7.70 (m, 1 H, CH=C<u>H</u>), 9.11 (s, 1 H, OH). ^{13}C NMR (101 MHz, DMSO-d_6) 55.67 (OCH_3), 56.17 (2×OCH_3), 60.16 (OCH_3), 105.99 (2×CH), 111.81 (CH), 114.86 (CH), 119.23 (CH), 122.30 (<u>CH</u>=CH), 127.67 (C), 133.29 (C), 141.72 (C-O), 144.38 (CH=<u>CH</u>), 146.69 (C-O), 150.29 (C-O), 152.86 (2×C-O), 187.71 (C=O) ppm. HRMS (EI): Found 343.1187 $(M-H)^+$; $C_{19}H_{19}O_6$ requires 343.1182.

(*E*)-1,3-Bis(3,4,5-trimethoxyphenyl)prop-2-en-1-one (20c). As per general method I, 3,4,5-trimethoxyacetophenone (1 eq, 7.1 mmol, 1.5 g) was reacted with 3,4,5-trimethoxybenzaldehyde (1 eq, 7.1 mmol, 1.39 g) and KOH 50% (10 mL) in methanol for 24 h. The precipitate was filtered and recrystallised from methanol, yield: 80%, 2.2 g, pale yellow crystals, Mp. 123-125°C [3]. IR: ν_{max} (ATR) cm^{-1}: 3385, 3001, 2919, 1660, 1578, 1501, 1466, 1324, 1266, 1225, 1148, 1120, 993, 971, 852, 830, 778, 725. ^{1}H NMR (400 MHz, $CDCl_3$) δ 3.89 (s, 3 H, OCH_3), 3.91 (s, 6 H, OCH_3), 3.93 (s, 3 H, OCH_3), 3.93 (s, 6 H, 2×OCH_3), 6.85 (s, 2 H, Ar-H), 7.24 (s, 2 H, Ar-H), 7.31 (d, J=15.34 Hz, 1 H, C<u>H</u>=CH), 7.70 (d, J=15.76 Hz, 1 H, CH=C<u>H</u>). ^{13}C NMR (101 MHz, $CDCl_3$) 56.29 (2×OCH_3), 56.51 (2×OCH_3), 60.99 (2x×OCH_3), 105.77 (2×CH), 106.28 (2×CH), 121.34 (<u>CH</u>=CH), 126.44 (C), 130.36 (C), 142.56 (C-O), 144.96 (C-O, CH=<u>CH</u>), 153.15 (2xC-O), 153.51 (2×C-O), 189.44 (C=O) ppm. HRMS (EI): Found 411.1420 $(M+Na)^+$ $C_{21}H_{24}NaO_7$ requires 411.1420.

(*E*)-3-(3,4-Dimethoxyphenyl)-1-(3,4,5-trimethoxyphenyl)prop-2-en-1-one (20d): As per general method I, 3,4,5-trimethoxyacetophenone (1 eq, 7.14 mmol, 1.5 g) was reacted with

3,4-dimethoxybenzaldehyde (1 eq, 7.14 mmol, 1.19 g) and KOH 50% (10 mL) in methanol for 24 h. The precipitate was filtered and recrystallised from methanol. Yield 90%, 2.3 g, yellow solid, Mp. 130-133 °C [1]. IR: ν_{max} (ATR) cm^{-1}: 3333, 2994, 2943, 2835, 1649, 1580, 1568, 1501, 1410, 1329, 1313, 1263, 1225, 1153, 1120, 1023, 997, 829, 806, 763, 698, 662. ^{1}H NMR (400 MHz, $CDCl_3$) δ 3.92 (s, 6 H, 2×OCH_3), 3.94 (s, 9 H, 3×OCH_3), 6.90 (d, *J*=8.29 Hz, 1 H, Ar-H), 7.14 (d, *J*=1.66 Hz, 1 H, Ar-H), 7.25 (d, *J*=1.66 Hz, 3 H, Ar-H), 7.31 (d, *J*=15.76 Hz, 1 H, CH=CH), 7.75 (d, *J*=15.76 Hz, 1 H, CH=CH). ^{13}C NMR (101 MHz, $CDCl_3$) 189.41 (C=O), 153.10 (2×C-O), 151.43 (C-O), 149.23 (C-O), 144.92 (CH=CH), 142.35 (C-O), 133.79 (C), 127.84 (C), 122.85 (CH), 119.85 (CH=CH), 111.15 (CH), 110.47 (CH), 106.13 (2×CH), 60.95 (OCH_3), 56.43 (2×OCH_3), 56.01 (OCH_3), 55.99 (OCH_3) ppm. HRMS (EI): Found 381.1314 $(M+Na)^+$; $C_{20}H_{22}NaO_6$ requires 381.1314.

(*E*)-3-(4-Ethoxyphenyl)-1-(3,4,5-trimethoxyphenyl)prop-2-en-1-one (20e): As per general method I, 3,4,5-trimethoxyacetophenone (1 eq, 4.7 mmol, 1.0 g) was reacted with 4-ethoxybenzaldehyde (1 eq, 4.7 mmol, 0.71 g, 0.67 mL) and KOH 50% (10 mL) in methanol for 24 h. The precipitate was filtered and recrystallised from methanol. Yield: 56%, 0.25 g, yellow solid, Mp. 73-77 °C [4] . IR: ν_{max} (ATR) cm^{-1}: 2998, 2933, 2820, 1678, 1586, 1567, 1407, 1355, 1329, 1215, 1174, 992, 887, 856, 750, 653. ^{1}H NMR (400 MHz, $CDCl_3$) δ 1.43 (t, *J*=7.05 Hz, 3 H, CH_3), 3.92 (s, 3 H, OCH_3), 3.94 (s, 6 H, 2×OCH_3), 4.07 (q, *J*=6.91 Hz, 2 H, CH_2), 6.92 (d, *J*=8.71 Hz, 2 H), 7.24 - 7.25 (m, 2 H, Ar-H), 7.34 (d, *J*=15.76 Hz, 1 H, CH=CH), 7.58 (d, *J*=8.71 Hz, 2 H), 7.78 (d, *J*=15.34 Hz, 1 H, CH=CH). ^{13}C NMR (101 MHz, $CDCl_3$) 14.71 (CH_3), 56.39 (2×OCH_3), 60.97 (OCH_3), 63.67 (CH_2), 106.00 (2×CH), 114.89 (2×CH), 119.28 (CH=CH), 127.42 (C), 130.23 (2×CH), 133.86 (C), 144.71 (C-O, CH=CH), 153.11 (2xC-O), 161.12 (C-OEt), 189.30 (C=O) ppm. HRMS (EI): Found 365.1346 $(M+Na)^+$; $C_{20}H_{22}NaO_5$ requires 365.1346.

(*E*)-3-(4-Fluorophenyl)-1-(3,4,5-trimethoxyphenyl)prop-2-en-1-one (20f): As per general method I, 3,4,5-trimethoxyacetophenone (1 eq, 7.14 mmol, 1.5 g) was reacted with 4-fluorobenzaldehyde (1 eq, 7.14 mmol, 0.89 g) and KOH 50% (10 mL) in methanol. The reaction was stirred at RT for 24 h. The precipitate was filtered and recrystallised from methanol. Yield: 81%, 1.8 g, yellow crystals, Mp. 127-130 °C [5]. IR: ν_{max} (ATR) cm^{-1}: 2947, 1692, 1625, 1600, 1572, 1525, 1424, 1346, 1314, 1277, 1199, 1088, 977, 815, 757. ^{1}H NMR (400 MHz, $CDCl_3$) δ 3.93 (s, 3 H, OCH_3), 3.94 (s, 6 H, 2×OCH_3), 7.11 (t, *J*=8.50 Hz, 2 H, Ar-H), 7.26 (s, 2 H, Ar-H), 7.39 (d, *J*=15.34 Hz, 1 H, CH=CH), 7.63 (dd, *J*=8.71, 5.39 Hz, 2 H, Ar-H), 7.77 (d, *J*=15.76 Hz, 1 H, CH=CH). ^{13}C NMR (101 MHz, $CDCl_3$) 56.43

(2×OCH_3), 60.98 (OCH_3), 106.11 (2×CH), 116.03 (CH), 116.25 (CH), 121.43 (<u>CH</u>=CH), 130.29 (C), 130.37 (2xCH), 131.13 (C), 143.41 (C-O, CH=<u>CH</u>), 153.17 (2xC-O), 165.31 (C-F), 188.97 (C=O) ppm. LRMS (EI): Found 339.21 $(M+Na)^+$; $C_{18}H_{17}FNaO_4$ requires 339.10.

(*E*)-3-Phenyl-1-(3,4,5-trimethoxyphenyl)prop-2-en-1-one (20g): As per general method I, 3,4,5-trimethoxyacetophenone (1 eq, 4.76 mmol, 1.0 g) was reacted with benzaldehyde (1 eq, 4.76 mmol, 0.505 g, 0.48 mL) and KOH 50% (10 mL) in methanol for 24 h. The precipitate was filtered and recrystallised from methanol. Yield: 86%, 1.22 g, yellow crystals, Mp. 80-84 °C [6]. IR: ν_{max} (ATR) cm^{-1}: 2941, 2838, 1656, 1571, 1450, 1412, 1335, 1228, 1155, 1121, 992, 844, 763, 634. ^1H NMR (400 MHz, $CDCl_3$) δ 3.93 (s, 3 H, OCH_3), 3.94 (s, 6 H, 2×OCH_3), 7.27 (s, 2 H, Ar-H), 7.40 - 7.44 (m, 3 H, Ar-H), 7.47 (d, *J*=15.76 Hz, 1 H, <u>CH</u>=CH), 7.64 (dd, *J*=6.84, 2.70 Hz, 2 H, Ar-H), 7.80 (d, *J*=15.34 Hz, 1 H, CH=<u>CH</u>). ^{13}C NMR (101 MHz, $CDCl_3$) 56.41 (2×OCH_3), 60.98 (OCH_3), 106.11 (2×CH), 121.80 (<u>CH</u>=CH), 128.42 (4×CH), 128.96 (CH), 130.55 (C), 133.49 (C), 144.74 (C-O, CH=<u>CH</u>), 153.15 (2×C-O), 189.25 (C=O) ppm. LRMS (EI): Found 321.29 $(M+Na)^+$; $C_{18}H_{18}NaO_4$ requires 321.11.

(*E*)-3-(4-Nitrophenyl)-1-(3,4,5-trimethoxyphenyl)prop-2-en-1-one (20h): As per general method I, 3,4,5-trimethoxyacetophenone (1 eq, 7.14 mmol, 1.5 g) was reacted with 4-nitrobenzaldehyde (1 eq, 7.14 mmol, 1.08 g) and KOH 50% (10 mL) in methanol at RT for 24 h. The precipitate was filtered and recrystallised from methanol. Yield: 83%, 2.02 g, yellow solid Mp. 197-200 °C [5]. IR: ν_{max} (ATR) cm^{-1}: 3294, 1526, 1294, 1221, 1085, 1031, 773. ^1H NMR (400 MHz, $CDCl_3$) δ 3.94 (s, 3 H, OCH_3), 3.95 (s, 6 H, 2×OCH_3), 7.27 (s, 2 H, Ar-H), 7.57 (d, *J*=15.76 Hz, 1 H, C<u>H</u>=CH), 7.78 (d, *J*=8.71 Hz, 2 H, Ar-H), 7.81 (d, *J*=15.76, 1 H, CH=C<u>H</u>), 8.28 (d, *J*=8.71 Hz, 2 H, Ar-H). ^{13}C NMR (101 MHz, $CDCl_3$) 188.27 (C=O), 153.27 (2×C-O), 148.54 (C-NO_2), 143.11 (C-O, CH=<u>CH</u>), 141.42 (C), 132.75 (C), 128.92 (2×CH), 125.39 (<u>CH</u>=CH), 124.21 (2×CH), 106.30 (2×CH), 61.03 (OCH_3), 56.48 (2×OCH_3) ppm. HRMS (EI): Found 344.1127 $(M+H)^+$; $C_{18}H_{18}NO_6$ requires 344.1134.

(*E*)-3-(4-Hydroxyphenyl)-1-(3,4,5-trimethoxyphenyl)prop-2-en-1-one (20j). As per general method I, 3,4,5-trimethoxyacetophenone (1 eq, 7.14 mmol, 1.5 g) was reacted with 4-hydroxybenzaldehyde (1 eq, 7.14 mmol, 0.87 g) and KOH 50% (10 mL) in methanol at RT for 24 h. The precipitate was filtered and recrystallised from methanol. Yield: 27%, 0.60 g, yellow solid, Mp. 200-204 °C [7]. IR: ν_{max} (ATR) cm^{-1}: 3142, 2945, 2833, 1638, 1586, 1551, 1438, 1342, 1306, 1275, 1231, 1168, 984, 824, 813, 702, 668. ^1H NMR (400 MHz, $CDCl_3$) δ 3.95 (s, 3 H, OCH_3) 3.96 (s, 6 H, 2×OCH_3) 6.88 - 6.93 (m, 2 H, Ar-H) 7.28 (s, 2 H, Ar-H) 7.36 (d, *J*=15.76 Hz, 1 H, <u>CH</u>=CH) 7.57 - 7.59 (m, 2 H, Ar-H) 7.79 (d, *J*=15.76 Hz, 1 H,

CH=C̲H̲). ^{13}C NMR (101 MHz, $CDCl_3$) 189.54 (C=O), 158.20 (C-OH), 153.08 (2×C-O), 144.81 (C-O, CH=C̲H̲), 133.72 (C), 130.46 (2×CH), 127.56 (C), 119.31 (C̲H̲=CH), 115.99 (2×CH), 106.04 (2×CH), 60.96 (OCH_3), 56.37 (2×OCH_3) ppm. HRMS (EI): Found 313.1083 $(M-H)^+$; $C_{18}H_{18}O_5$ requires 313.1076.

(E)-3-(4-Chlorophenyl)-1-(3,4,5-trimethoxyphenyl)prop-2-en-1-one 20k:

General Procedure I was followed using 1-(3,4,5-trimethoxyphenyl)ethan-1-one (1 equiv; 2.37 mmol; 500 mg), 4-chlorobenzaldehyde (1 equiv; 2.37 mmol; 334 mg), MeOH (30 mL) and 70 drops of 50% KOH solution to afford the pure product **20l** as a white powder (91%)[8]. ^{1}H NMR (400 MHz, $CDCl_3$): δ ppm 7.75 (d, J = 15.7 Hz, 1H), 7.57 (d, J = 8.5 Hz, 2H), 7.43 (d, J = 15.7 Hz, 1H), 7.39 (d, J = 8.5 Hz, 2H), 7.25 (s, 2H), 3.94 (s, 6H), 3.92 (s, 3H). ^{13}C NMR (400MHz, $CDCl_3$): ppm 188.91, 153.18, 143.26, 142.84, 136.32, 133.34, 133.25, 129.55, 129.34, 122.02, 106.10, 61.05, 56.48 ppm.

General method II: Preparation of (*E*)-1,3-Diarylprop-2-en-1-ols (21a-c)

To a solution of the appropriate chalcone (1 eq) in methanol (25 mL), a suspension of $NaBH_4$ (1 eq) in methanol (10 mL) and THF (10 mL) was slowly added. The reaction mixture was stirred (0-20 °C) and monitored by TLC until the reaction was complete. $NaHCO_3$ (sat., 5 mL) was then added and the reaction mixture was concentrated. The reaction residue was extracted with ethyl acetate, washed with water and brine and dried over sodium sulphate. No further purification was required.

(*E*)-3-(4-Methoxyphenyl)-1-(3,4,5-trimethoxyphenyl)prop-2-en-1-ol (21a): As per general method II (*E*)-3-(4-methoxyphenyl)-1-(3,4,5-trimethoxyphenyl)prop-2-en-1-one **(20a) (**1 eq, 1.5 mmol, 0.5 g) was treated with sodium borohydride (1 eq, 1.5 mmol, 0.06 g) in methanol (10 mL) and THF (10 mL). Yield: 100%, 1.45 g, white solid, Mp. 113-116 °C [1]. IR: ν_{max} (ATR) cm^{-1}: 3379, 2836, 1693, 1508, 1458, 1421, 1243, 1123, 1037, 1020, 848, 767. ^{1}H NMR (400 MHz, $CDCl_3$) δ 3.81 (s, 3 H, OCH_3), 3.84 (s, 3 H, OCH_3), 3.87 (s, 6 H, 2×OCH_3), 5.30 (d, *J*=9.54 Hz, 1 H, CH-OH), 6.23 (dd, *J*=15.76, 7.05 Hz, 1 H, CH̲=CH), 6.59 (d, *J*=16.59 Hz, 1 H, CH=CH̲), 6.66 (s, 2 H, Ar-H), 6.83 - 6.87 (m, 2 H, Ar-H), 7.32 - 7.36 (m, 2 H, Ar-H).^{13}C NMR (101 MHz, $CDCl_3$) 55.29 (OCH_3), 56.13 (2×OCH_3), 60.82 (OCH_3), 75.44 (CH-OH), 103.12 (2×CH), 114.01 (2×CH), 127.85 (C̲H=CH), 129.07 (C), 129.14 (CH=C̲H), 130.42 (2×CH), 135.76 (C), 138.73 (C-O), 153.38 (2×C-O), 159.44 (C-O) ppm. HRMS (EI): Found 329.1383 $(M-H)^{+;}$ $C_{19}H_{21}O_5$ requires 329.1389.

(*E*)-5-(3-Hydroxy-3-(3,4,5-trimethoxyphenyl)prop-1-en-1-yl)-2-methoxyphenol (21b): As per general method II (*E*)-3-(3-hydroxy-4-methoxyphenyl)-1-(3,4,5-trimethoxyphenyl)prop-2-en-1-one **(20b)** (1 eq, 5.22 mmol, 1.9 g) was treated with sodium borohydride (1 eq, 5.22 mmol, 0.2 g) in methanol (10 mL) and THF (10 mL). Yield: 100%, 1.8 g, yellow oil [1]. IR: ν_{max} (ATR) cm^{-1}: 3448, 2988, 2971, 2939, 1582, 1503, 1454, 1416, 1327, 1233, 1120, 1044, 911, 829, 598, 561. ^{1}H NMR (400 MHz, $CDCl_3$) δ 3.82 (s, 3 H, OCH_3), 3.85 (s, 6 H, 2×OCH_3), 3.87 (s, 3 H, OCH_3), 5.27 (d, *J*=6.63 Hz, 1 H, CH-OH), 6.20 (dd, *J*=15.76, 6.63 Hz, 1 H, CH=CH), 6.54 - 6.60 (m, 1 H, CH=CH), 6.64 (s, 2 H, Ar-H), 6.78 (d, *J*=8.29 Hz, 1 H, Ar-H), 6.86 (dd, *J*=8.29, 2.07 Hz, 1 H, Ar-H), 7.01 (d, *J*=2.07 Hz, 1 H, Ar-H). ^{13}C NMR (101 MHz, $CDCl_3$) 153.09 (2xC-O), 148.80 (C-O), 144.64 (C-OH), 142.29 (C-O), 131.67 (C), 128.53 (C), 124.46 (CH=CH), 122.98 (CH=CH), 119.83 (CH), 112.69 (CH), 110.55 (CH), 106.71 (CH), 105.93 (CH), 68.04 (CH-OH), 60.98 (OCH_3), 56.35 (OCH_3), 56.26 (OCH_3), 56.21 (OCH_3) ppm. HRMS (APCI): Found 343.1185 $(M-3H)^+$ $C_{19}H_{19}O_6$ requires 343.1182.

(*E*)-1,3-Bis(3,4,5-trimethoxyphenyl)prop-2-en-1-ol (21c): As per general method II (*E*)-1,3-bis(3,4,5-trimethoxyphenyl)prop-2-en-1-one **(20c)** (1 eq, 1.28 mmol, 0.5 g) was reacted with sodium borohydride (2 eq, 2.57 mmol, 0.1 g) in methanol and THF. Yield: 93%, 0.46 g, yellow solid, Mp. 58-62 °C [9]. IR: ν_{max} (ATR) cm^{-1}: 3494, 2938, 2836, 1582, 1503, 1453, 1416, 1325, 1231, 1119, 1001, 818, 778, 731, 700, 663. ^{1}H NMR (400 MHz, $CDCl_3$) δ 6.60 (s, 2 H, Ar-H), 6.59 (s, 2 H, Ar-H), 6.51 (d, *J* = 16.6 Hz, 1 H, CH=CH), 6.14 (dd, *J* = 15.8, 6.8 Hz, 1 H, CH=CH), 4.69 (d, *J* = 6.8 Hz, 1 H, CH-OH), 3.86 (s, 6 H, 2×OCH_3), 3.84 (s, 6 H, OCH_3), 3.82 (s, 3 H, OCH_3), 3.81 (s, 3 H, OCH_3). ^{13}C NMR (101 MHz, $CDCl_3$) 153.37 (2×C-O), 153.26 (2×C-O), 137.98 (C-O), 137.37 (C-O), 136.65 (C), 132.13 (C), 131.46 (CH=CH), 129.31 (CH=CH), 103.68 (2xCH), 103.57 (2×CH), 84.30 (CH-OH), 60.88 (OCH_3), 60.80 (OCH_3), 56.13 (2×OCH_3), 56.07 (2×OCH_3) ppm. HRMS (EI): Found 413.1556 $(M+Na)^+$; $C_{21}H_{26}NaO_7$ requires 413.1576.

3-(3-Hydroxy-4-methoxyphenyl)-4,5,6-trimethoxy-2,3-dihydro-1*H*-inden-1-ol (25a): As per general method II above, 3-(3-hydroxy-4-methoxyphenyl)-4,5,6-trimethoxy-2,3-dihydro-1*H*-inden-1-one **(24a) (**1 eq, 1.21 mmol, 0.42 g) was reacted with sodium borohydride (2 eq, 2.42 mmol, 0.10 g) in methanol and THF. Yield: 86% (0.36 g) brown resin [10]. IR: ν_{max} (ATR) cm^{-1}: 3405, 2969, 2936, 2988, 1691, 1594, 1465, 1431, 1414, 1507, 1265, 1049, 1113, 1023, 727, 761, 590. ^{1}H NMR (400 MHz, $CDCl_3$) δ 1.89 - 1.95 (m, 1 H, CH_2), 2.95 (ddd, *J*=13.79, 8.40, 7.26 Hz, 1 H, CH_2), 3.46 (s, 3 H, OCH_3), 3.82 (s, 3 H, OCH_3), 3.86 (s, 3 H,

OCH_3), 3.90 (s, 3 H, OCH_3), 4.25 (dd, *J*=8.29, 5.39 Hz, 1 H, CH), 5.14 (dd, *J*=7.26, 4.77 Hz, 1 H, CH-OH), 5.59 (s, 1 H, OH), 6.70 (d, *J*=2.07 Hz, 1 H, Ar-H), 6.75 (s, 1 H, Ar-H), 6.80 - 6.82 (m, 2 H, Ar-H). ^{13}C NMR (101 MHz, $CDCl_3$) 46.21 (CH_2), 46.42 (C), 55.93 (OCH_3), 56.12 (2×OCH_3), 60.79 (OCH_3), 75.87 (CH-OH), 102.65 (CH), 110.45 (CH), 113.89 (CH), 118.82 (CH), 139.62 (C), 140.56 (C), 142.61 (C), 144.92 (C-O), 145.44 (2xC-O), 150.11 (C-O) 154.15 (C-O) ppm. HRMS (EI): Found 345.1334 $(M-H)^+$; $C_{19}H_{21}O_6$ requires 345.1338.

General method III synthesis of anthracene-base chalcones 31a, 31b

NaOH (1.3 equiv; 6 mmol, 240 mg) was dissolved in 50% aqueous ethanol (20 mL). The required ketone (5.02 mmol) was dissolved EtOH (20 mL) and then added before adding a solution of anthracene-9-carbaldehyde (5.02 mmol; 1.035 g) dissolved EtOH (40 mL) . The mixture was left to stir at RT for 24 h. The resulting suspension was filtered, washed with minimal EtOH to afford the crude product before recrystallization from minimal hot EtOH to afford the pure product .

(*E*)-3-(anthracen-9-yl)-1-(4-iodophenyl)prop-2-en-1-one 31a

General procedure III was followed using 1-(4-iodophenyl)ethan-1-one (1 equiv; 5.02 mmol; 1.235 g) to afford the product as yellow needle-like crystals (66%) Mp. 167-173 ºC. ^1H NMR (400 MHz, $CDCl_3$) δ 7.44 - 7.54 (m, 5 H, 4×ArH, 1xC=CH), 7.75 - 7.80 (m, 2 H, ArH), 7.84 - 7.89 (m, 2 H, ArH), 7.99 - 8.05 (m, 2 H, ArH), 8.24 - 8.29 (m, 2 H, ArH), 8.46 (br.s, 1 H, ArH), 8.80 (d, *J*=16.17 Hz, 1 H, C=CH). ^{13}C NMR (101 MHz, $CDCl_3$) ppm 101.06, 125.17, 125.46, 126.54, 128.65, 128.96, 129.63, 130.06, 129.86, 130.33, 131.28, 137.12, 138.05, 142.46, 188.80 (C=O). IR$_{Vmax}$ (ATR): 3045.07 (Ar C-H), 1593.90 (C=O), 1618.99 (*trans* C=C) 1658.74, 1577.36, 1515.35, 1439.81 (Ar C=C), 669.23 (C-I) cm^{-1}.

(*E*)-3-(Anthracen-9-yl)-1-(pyridin-4-yl)prop-2-en-1-one 31b

Preparation from 4-acetylpyridine (5.02 mmol, 0.55 mL) and 9-anthracenecarboxaldehyde (5.02 mmol, 1.035 g), according to the general procedure II above; orange crystals (49%), Mp. 170-173 ºC. ^1H NMR (400 MHz, $CDCl_3$) δ 7.44 - 7.48 (d, 1H, CH=C), 7.49 - 7.56 (m, 3H, ArH), 7.80 - 7.85 (m, 2H, ArH), 7.98 - 8.05 (m, 2H, ArH), 8.21 - 8.28 (m, 2H, Ar H), 8.46 (br.s, 1H), 8.80 - 8.87 (m, 3H, ArH, C=CH). ^{13}C NMR (101 MHz, $CDCl_3$) ppm 121.57, 124.92, 125.49, 126.75, 129.03 129.09, 129.64, 129.84, 131.23, 143.86, 143.90, 150.94, 188.94 (C=O). IR *v* max (KBr): 3047.23, 2971.86 (Ar C-H), 1587 (C=O), 1621.69 (*trans* C=C), 1661. 62, 1597.45, 1518.42, 1441.76 (Ar C=C), 1266.30 (C-N) cm^{-1}.

22a

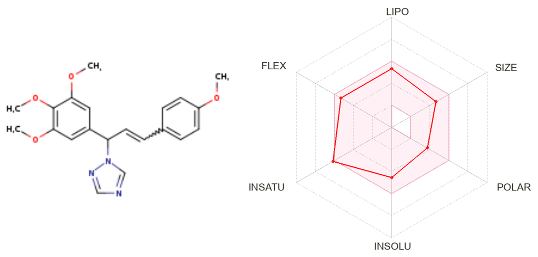

22b

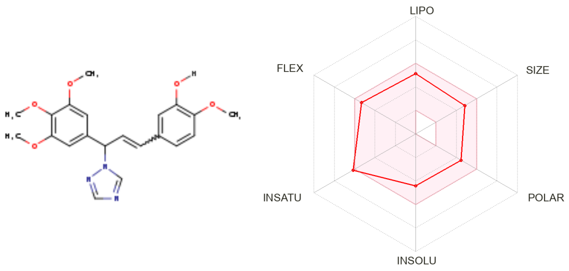

23a

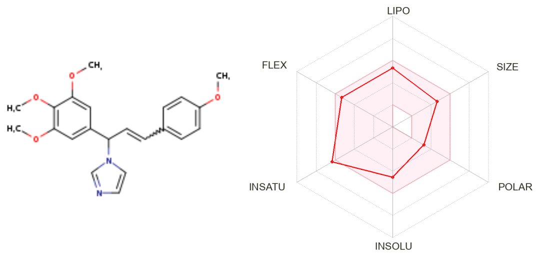

23b

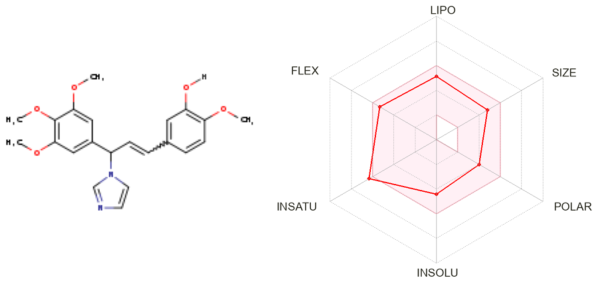

Figure S1: Bioavailability Rader for **22a, 22b, 23a** and **23b**.

The following six physicochemical properties are considered for analysis of drug-likeness: lipophilicity (XLOGP3), size (MW), polarity (TPSA, topological polar surface area), solubility (logS), saturation (fsp^3) and flexibility (number of rotatable bonds). A physicochemical range on each axis is depicted as a pink area in which the radar plot of the molecule has to fall entirely to be considered drug-like. The pink area represents the optimal range for each properties (lipophilicity: XLOGP3 between −0.7 and +5.0, size: MW between 150 and 500 g/mol, polarity: TPSA between 20 and 130 Å^2, solubility: log *S* not higher than 6, saturation: fraction of carbons in the sp^3 hybridization not less than 0.25, and flexibility: no more than 9 rotatable bonds. (SwissADME http://www.swissadme.ch).

22a

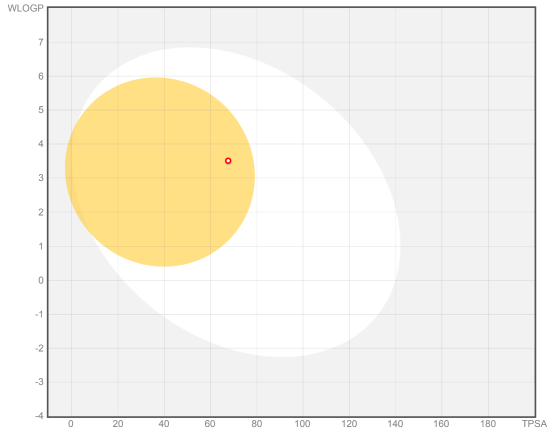

22b

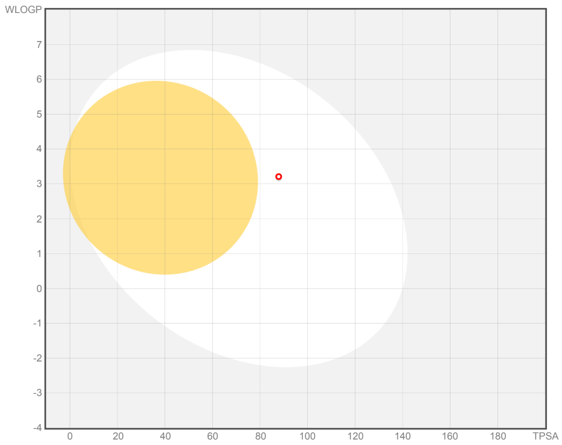

23a

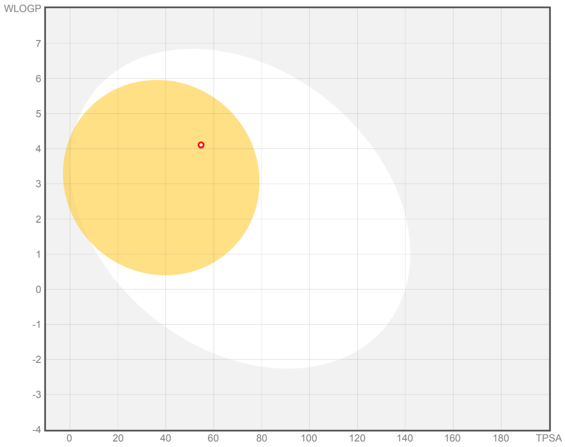

23b

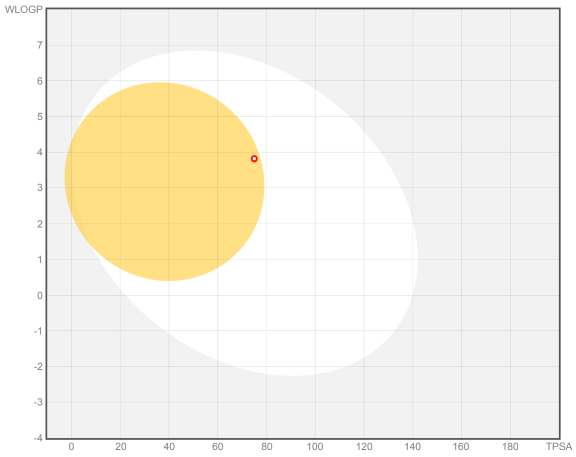

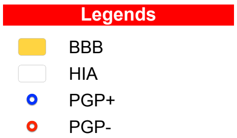

Figure S2: The BOILED-Egg allows evaluation of passive gastrointestinal absorption (HIA) and brain penetration (BBB) of compound **22a, 22b, 23a** and **23b** as function of the position of the molecule in the WLOGP-*versus*-TPSA map.

Boiled-Egg is a visual prediction model for the evaluation of passive gastrointestinal absorption (HIA) and blood-brain-barrier (BBB) penetration. The data for compound **22b** is visually analysed on the same 2D map with WLOGP and TPSA plot. (SwissADME http://www.swissadme.ch).

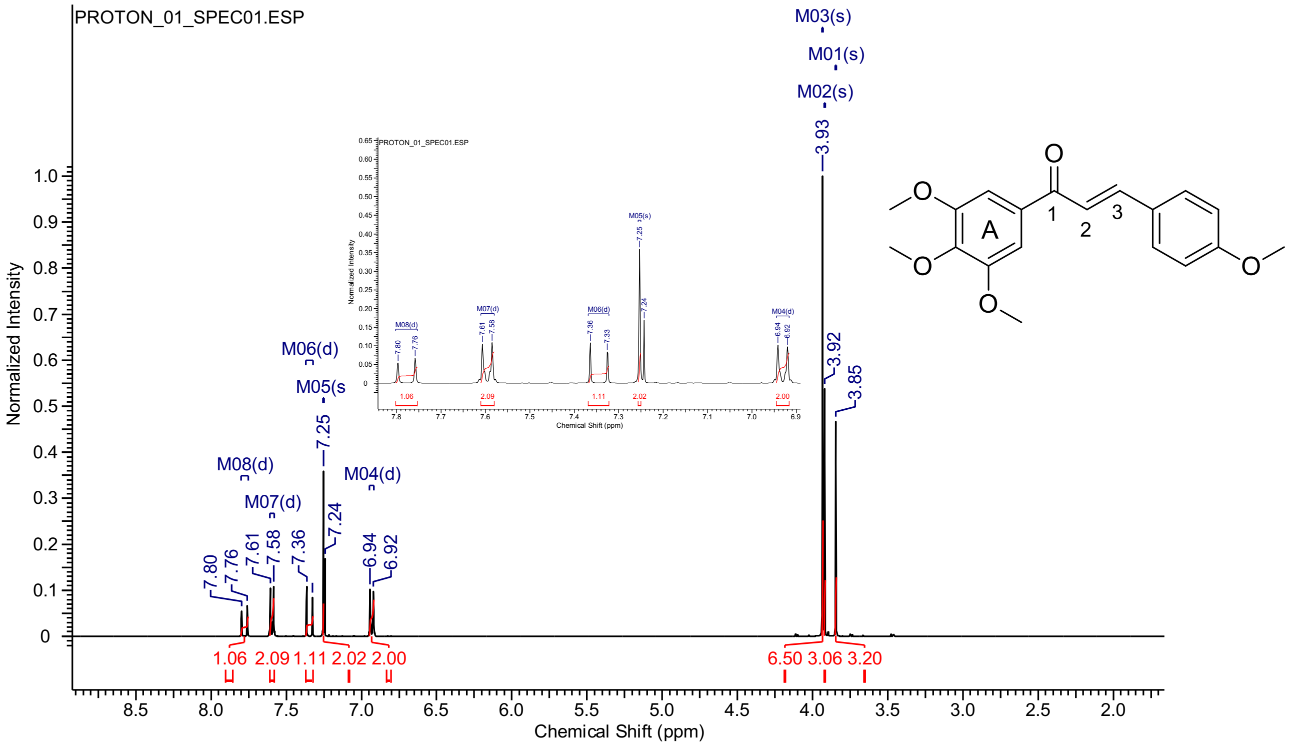

Figure S3: ^{1}H-NMR spectrum of compound 20a ($CDCl_3$)

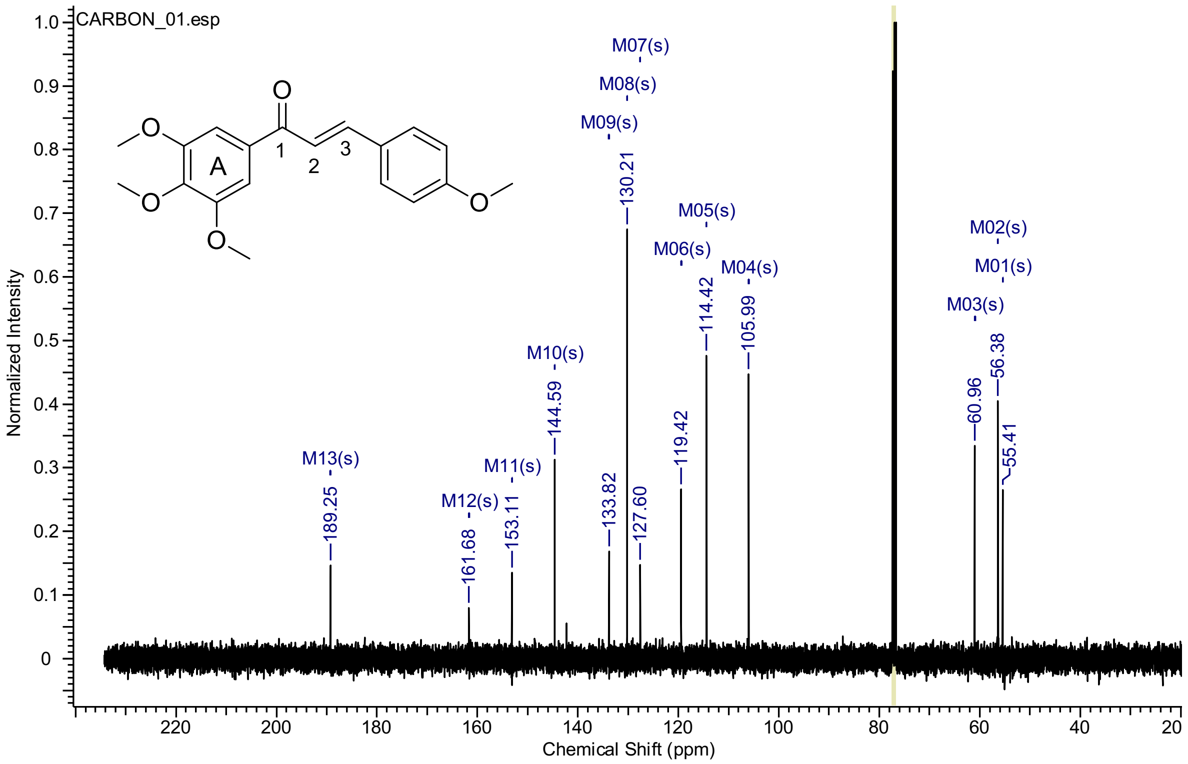

Figure S4: ^{13}C-NMR spectrum of compound 20a ($CDCl_3$)

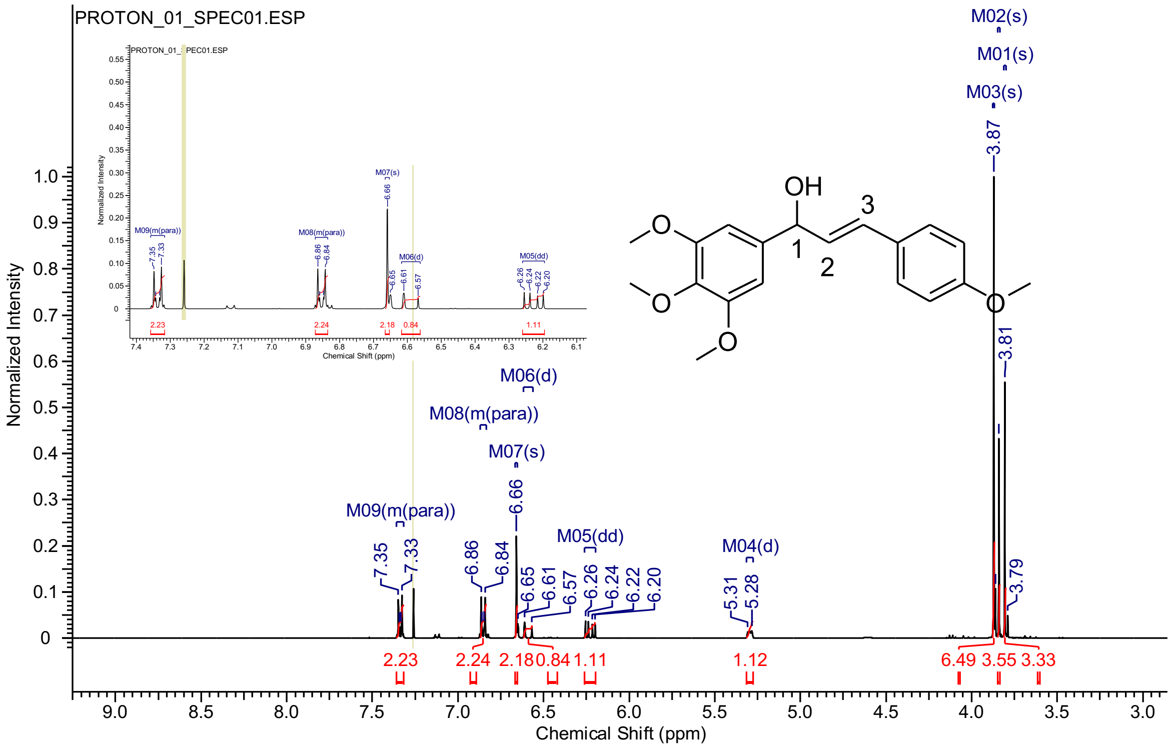

Figure S5: ^{1}H-NMR spectrum of compound 21a ($CDCl_3$)

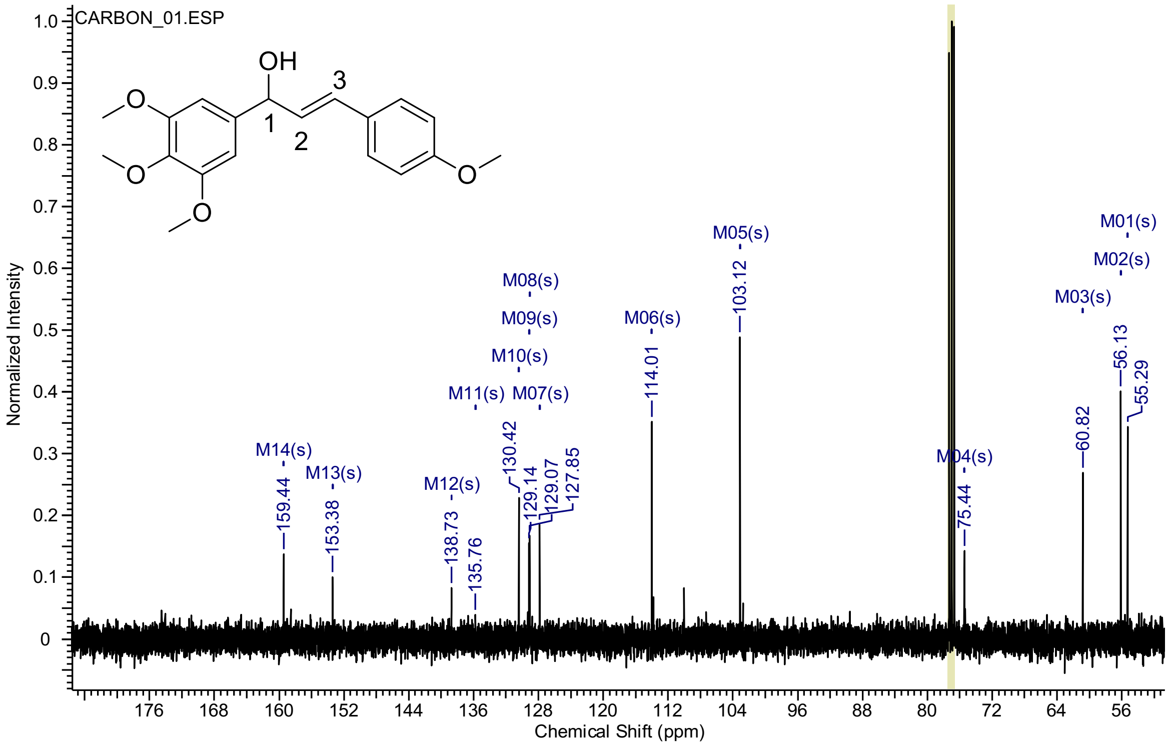

Figure S6: ^{13}C-NMR spectrum of compound 21a ($CDCl_3$)

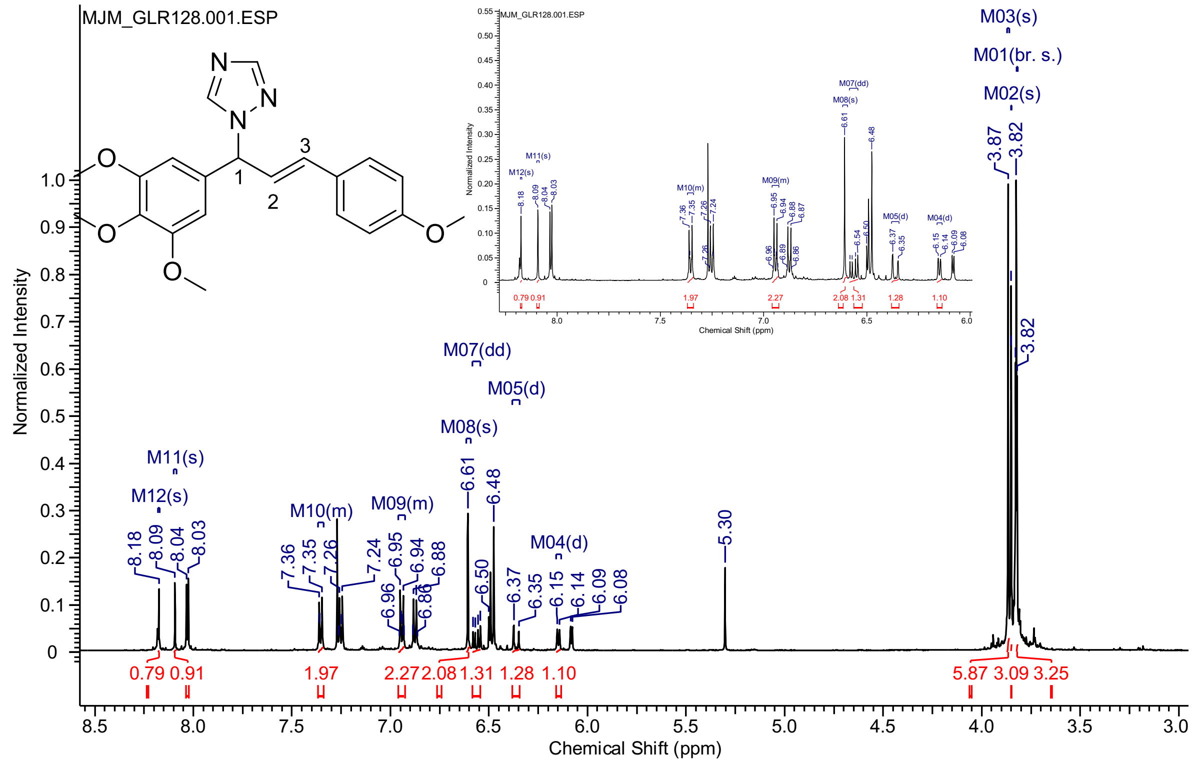

Figure S7: ^{1}H-NMR spectrum of compound 22a ($CDCl_3$)

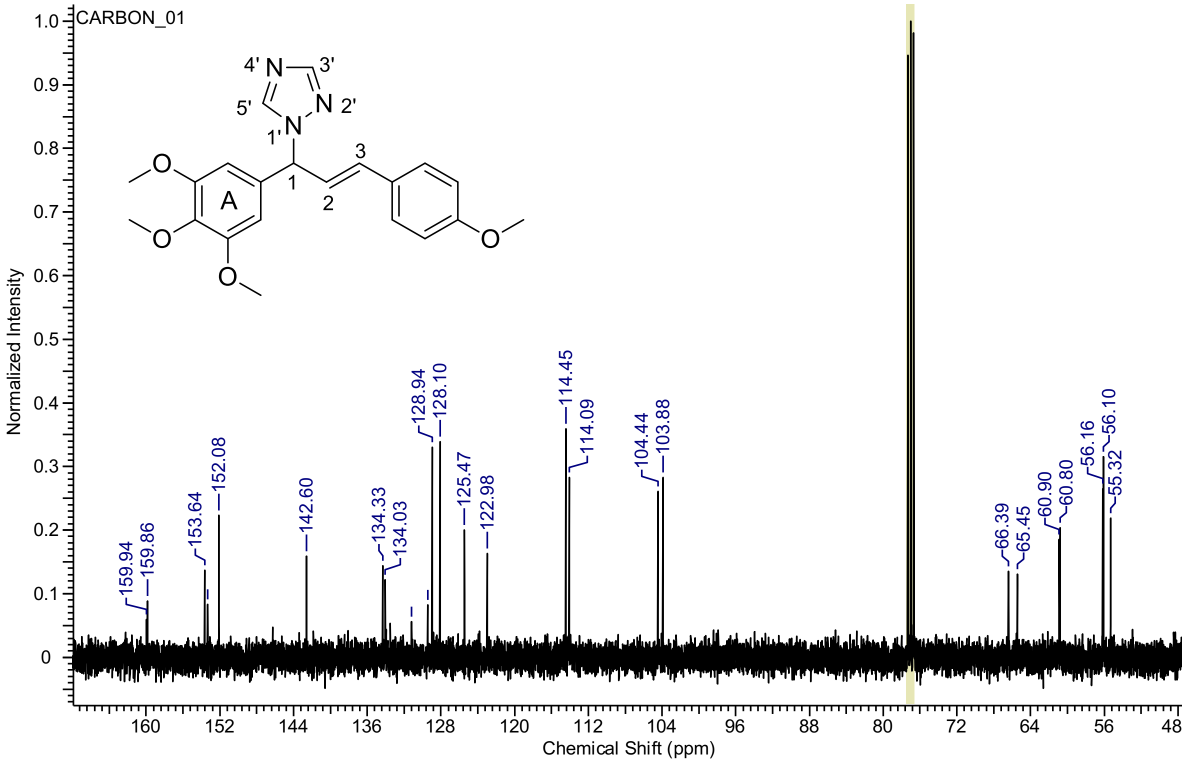

Figure S8: ^{13}C-NMR spectrum of compound 22a ($CDCl_3$)

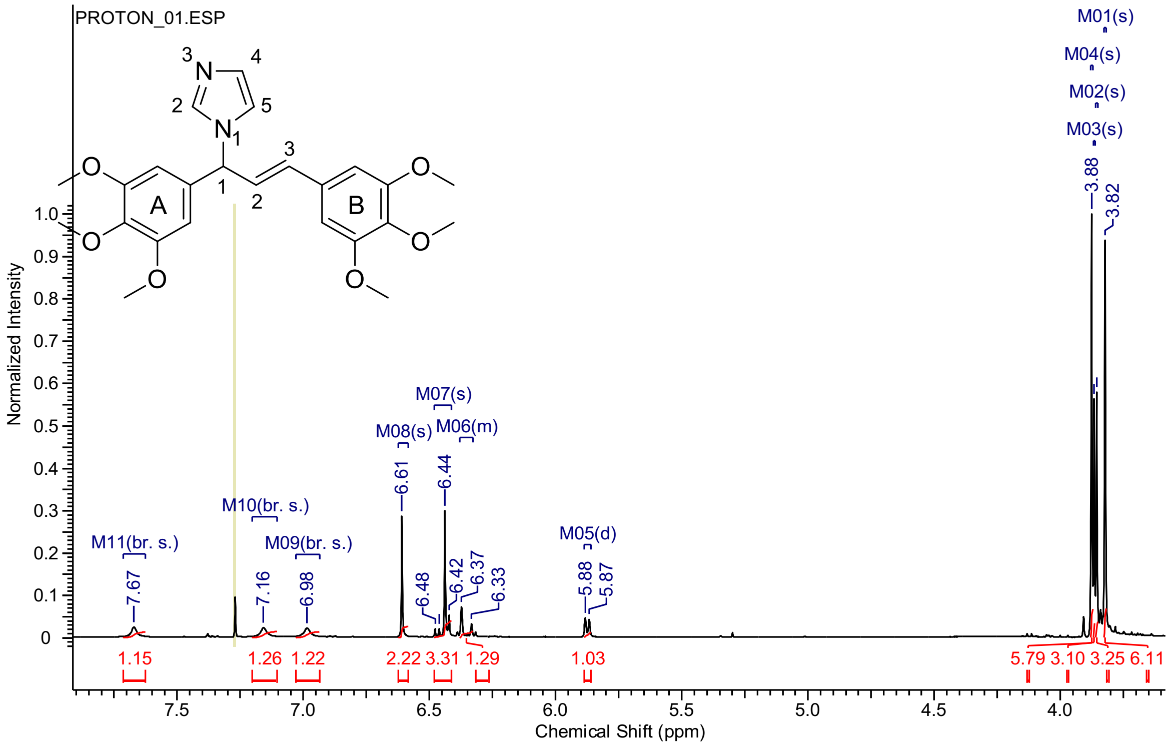

Figure S9: ^{1}H-NMR spectrum of compound 23c ($CDCl_3$)

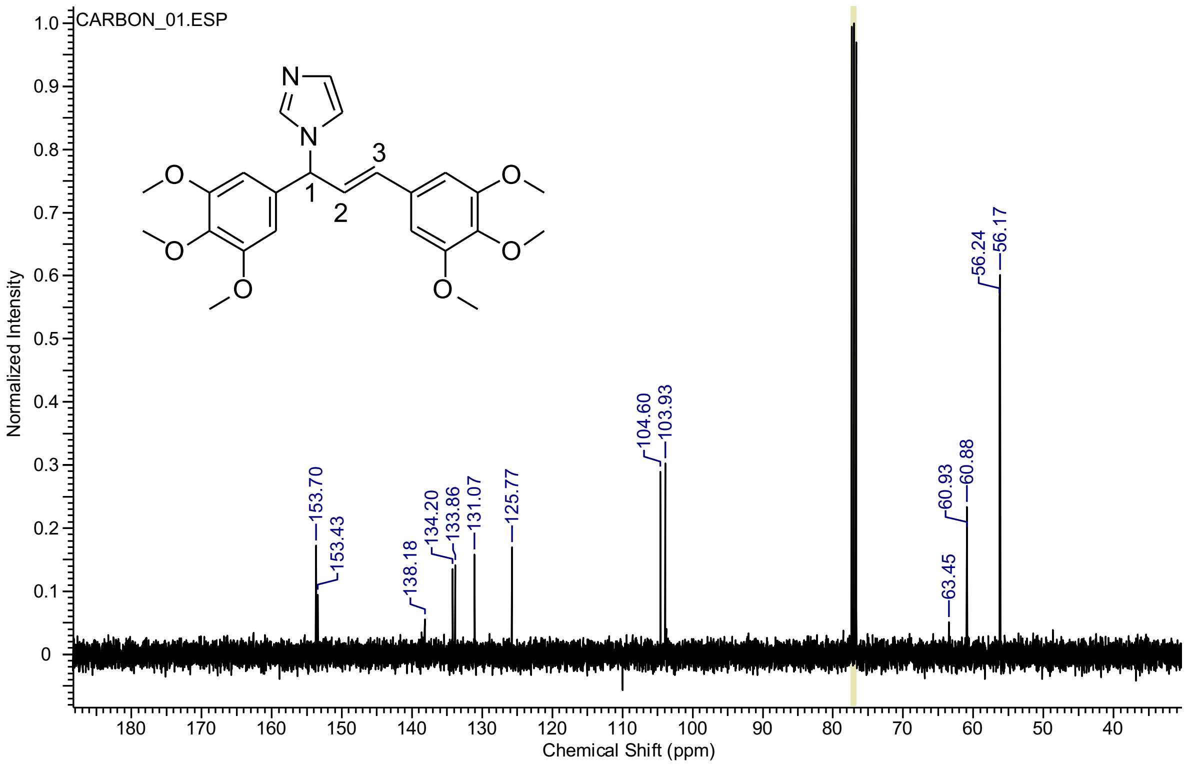

Figure S10: ^{13}C-NMR spectrum of compound 23c ($CDCl_3$)

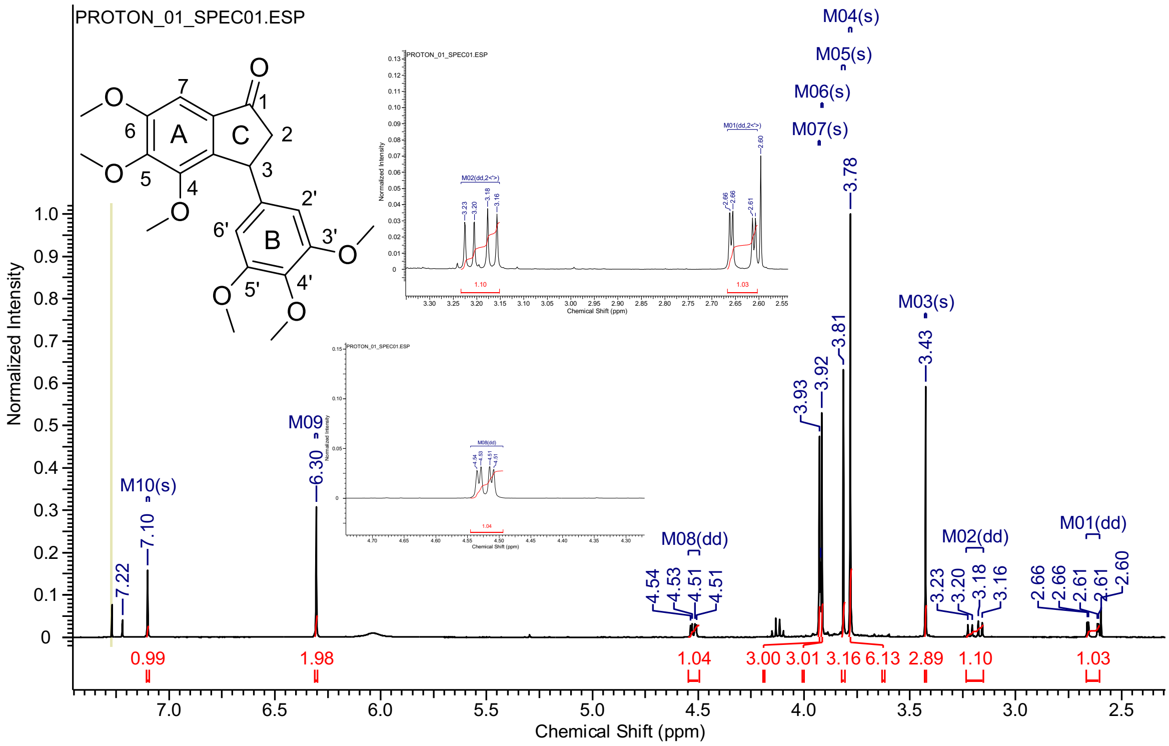

Figure S11: ^{1}H-NMR spectrum of compound 24b ($CDCl_3$)

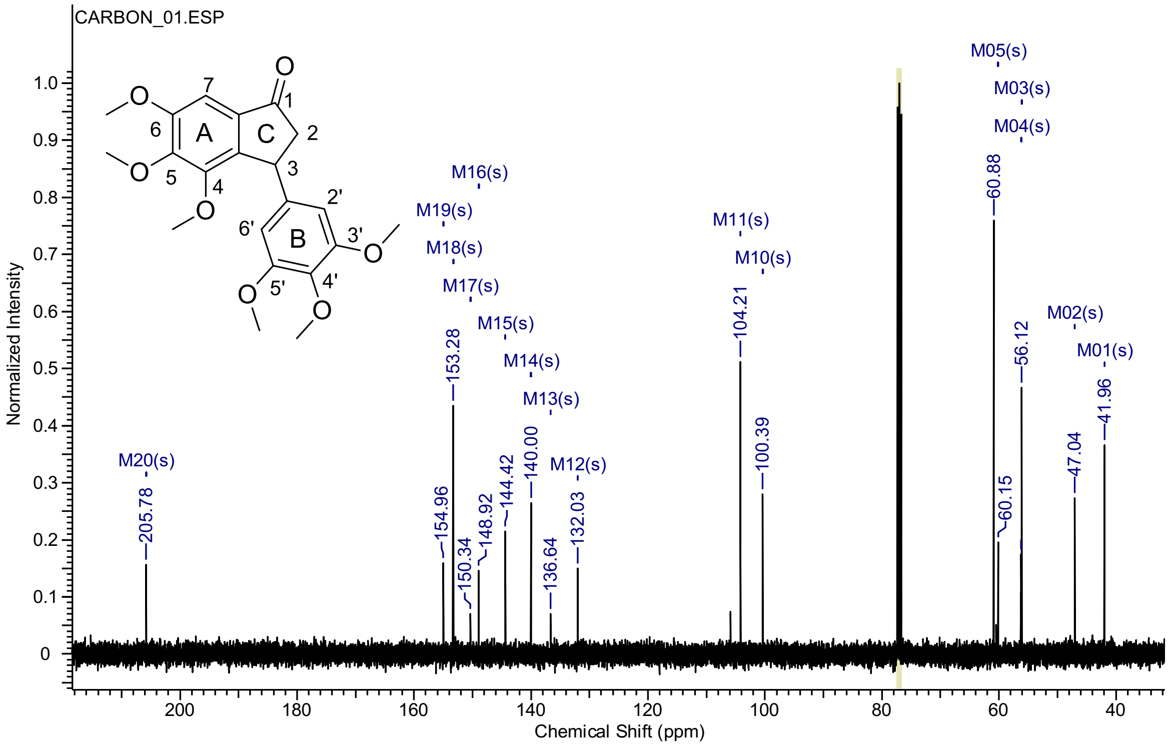

Figure S12: ^{13}C-NMR spectrum of compound 24b ($CDCl_3$)

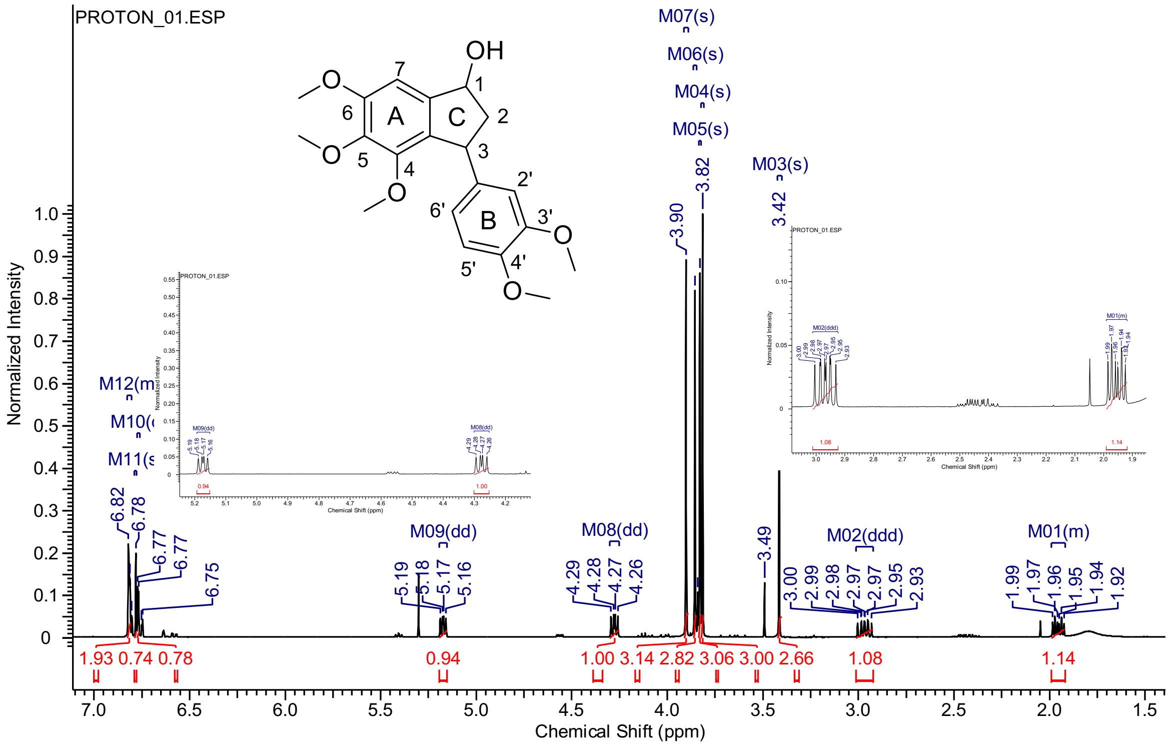

Figure S13: ^{1}H-NMR spectrum of compound 25c ($CDCl_3$)

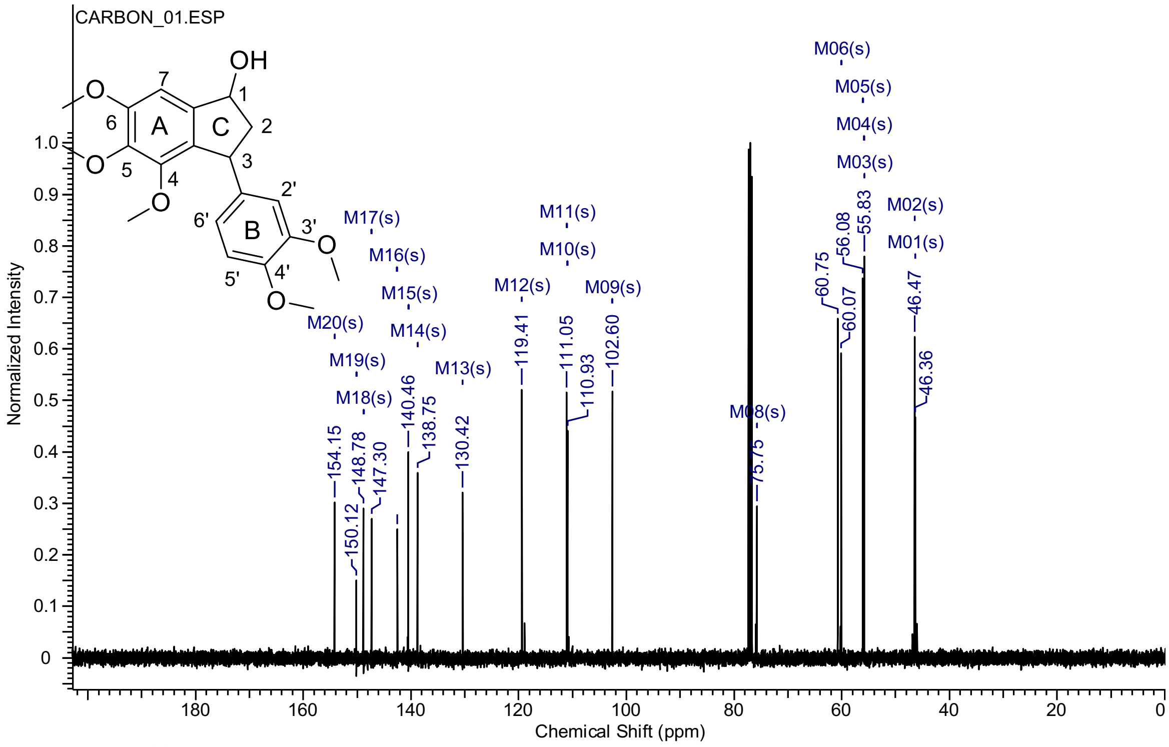

Figure S14: ^{13}C-NMR spectrum of compound 25c ($CDCl_3$)

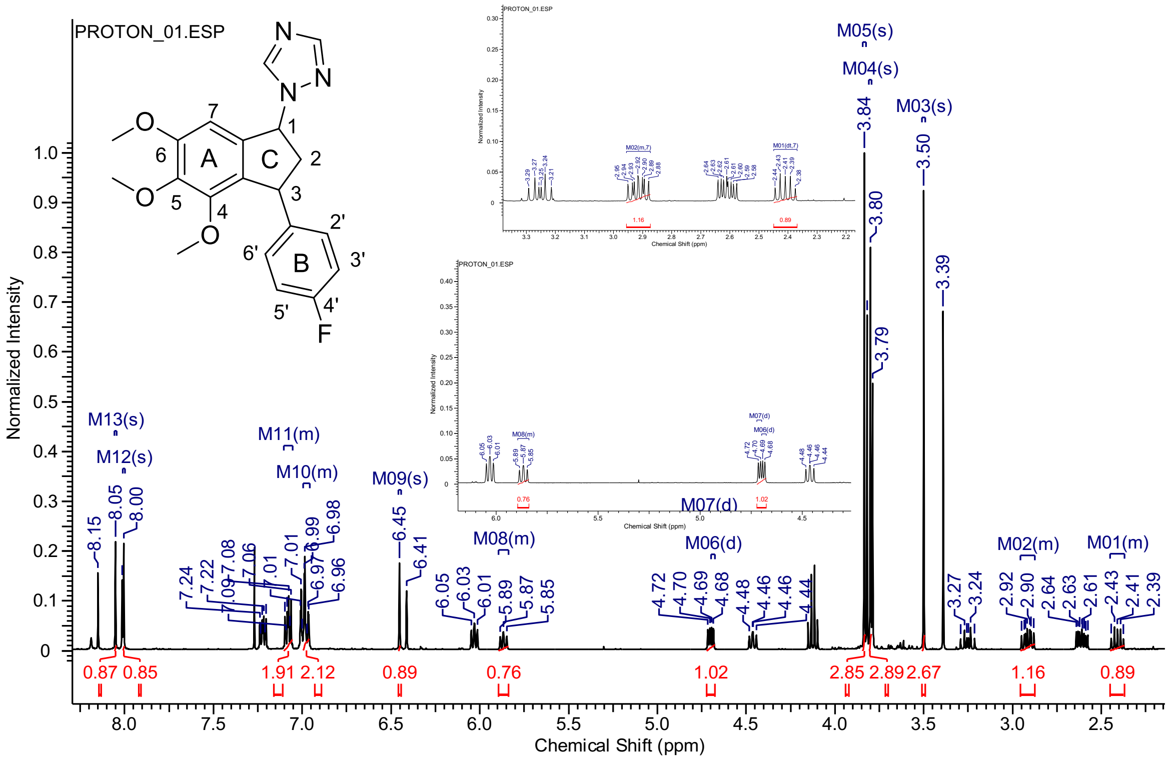

Figure S15: ^{1}H-NMR spectrum of compound 26d ($CDCl_3$)

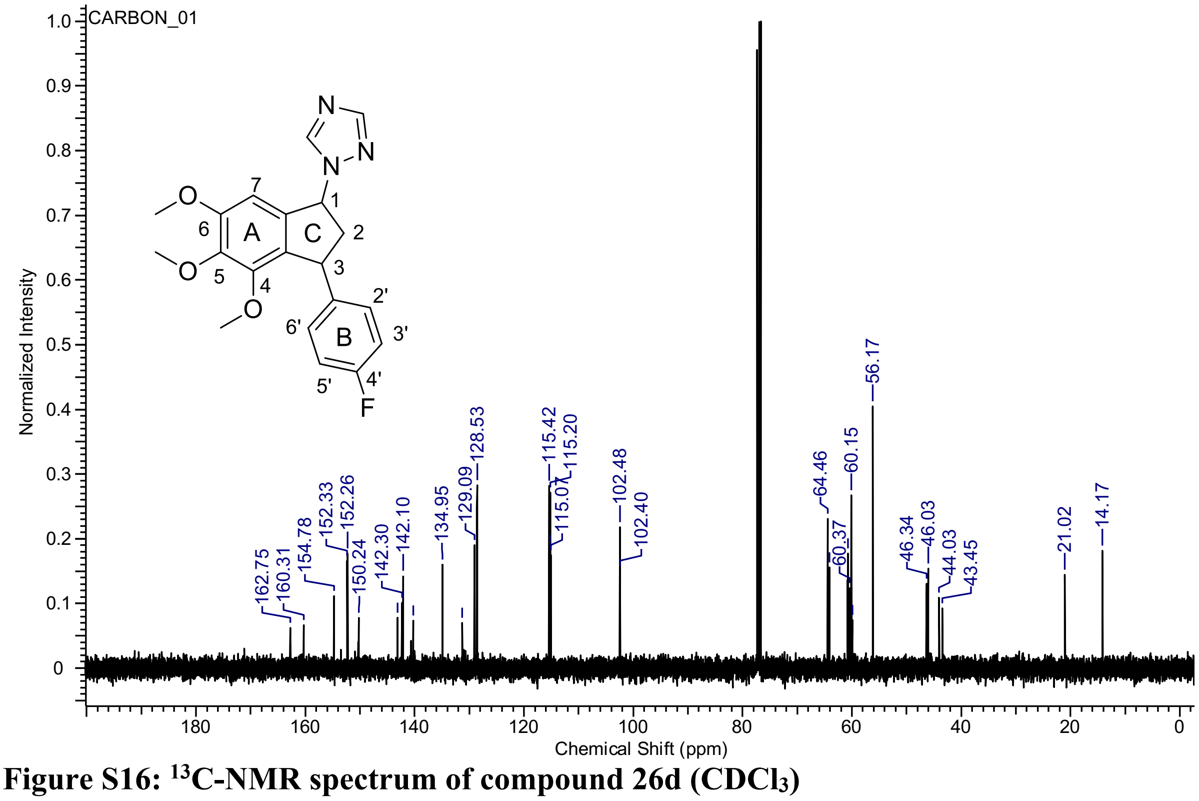

Figure S16: ^{13}C-NMR spectrum of compound 26d ($CDCl_3$)

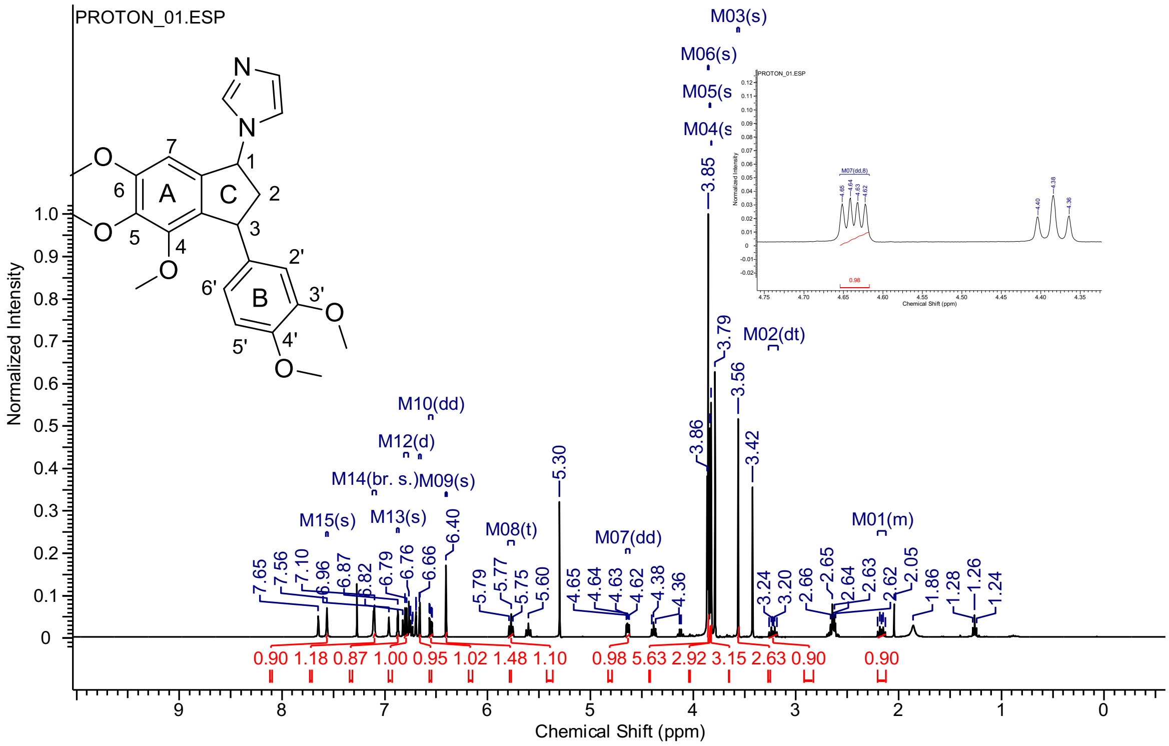

Figure S17: ^{1}H-NMR spectrum of compound 27c ($CDCl_3$)

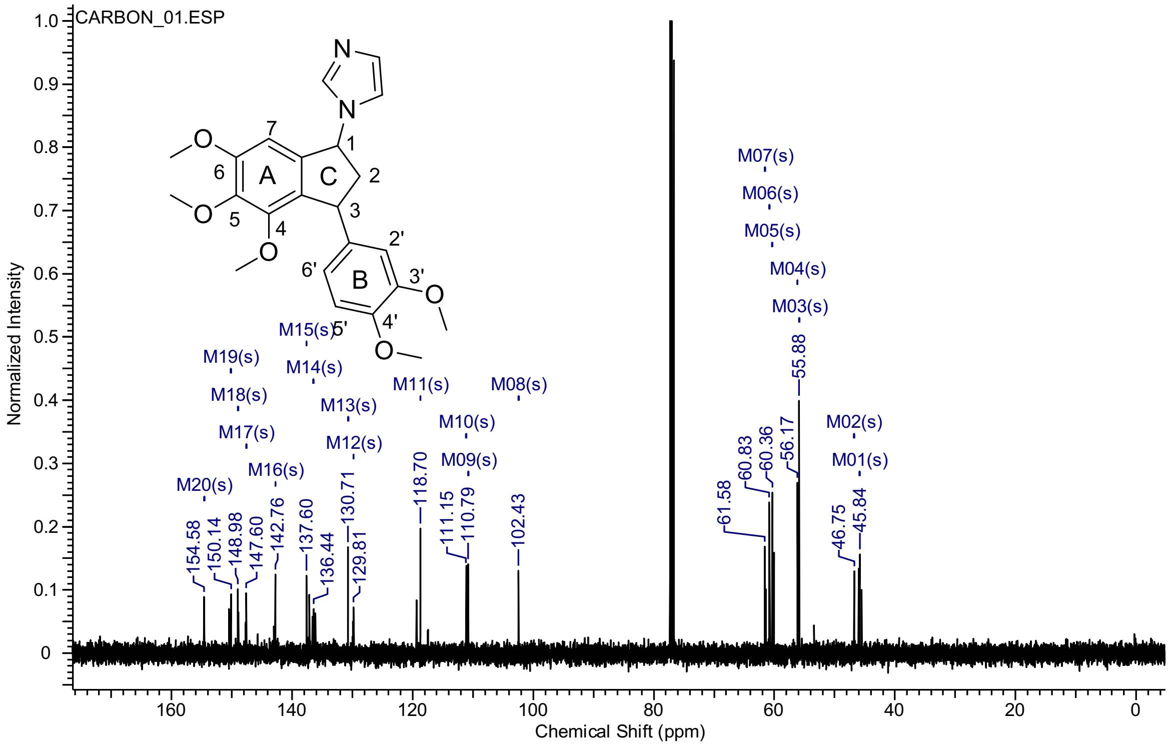

Figure S18: ^{13}C-NMR spectrum of compound 27c ($CDCl_3$)

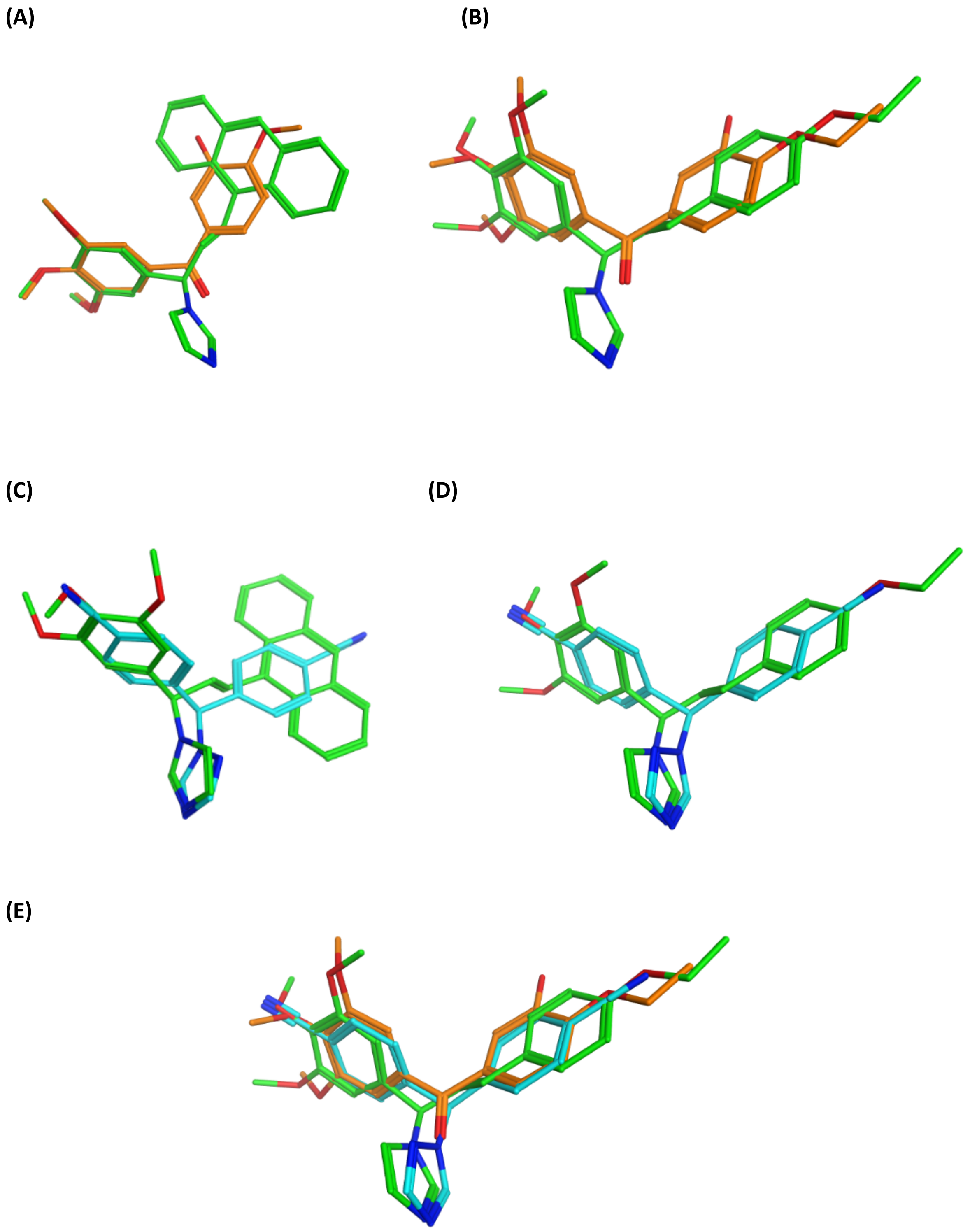

Figure S19: Overlay of **(A)** anthracene based imidazole-chalcone with phenstatin; **(B)** imidazole-chalcone with Phenstatin; **(C)** anthracene-based chalcone and letrozole; **(D)** imidazole-chalcone with letrozole and **(E)** imidazole-chalcone with letrozole and phenstatin

Table S1: Standard COMPARE Analysis of compound 22b

Rank	Compound	*r*
	**Based on GI_{50} mean graph**	
1	Glycoxalic acid	0.595
2	Dichloroallyl lawsone	0.592
3	S-Trityl-L-cysteine	0.58
4	Vinblastine sulfate	0.578
5	DUP785 (brequinar)	0.569
	**Based on TGI mean graph**	
1	Paclitaxel (Taxol) hiConc:-6.0	0.703
2	S-Trityl-L-cysteine	0.648
3	Vinblastine sulfate	0.643
4	Paclitaxel (Taxol) hiConc:-4.6	0.641
5	Rhizoxin	0.586

The target set was the standard agent database and the target set endpoints were selected to be equal to the seed end points. Standard COMPARE analysis was performed. Correlation values (*r*) are Pearson correlation coefficients. Paclitaxel appears at different concentrations as the drug has been tested by the NCI at multiple concentration ranges. LC_{50} values all > 0.1 mM.

Table S2: Physicochemical descriptors of selected (*E*)-1-(3-(4-methoxyphenyl)-1-(3,4,5-trimethoxyphenyl)allyl)-1*H*-1,2,4-triazoles and related compounds [a].

Cpd No.	MW	Molar Refractivity (m^3/mol)	Num. HBD	Num. HBA	Num. Rot Bonds	TPSA ($Å^2$)	Log P (consensus)[c]	Water Solubility (mg/mL)[d]	Solubility Class[e]	GI Absorption[b]
**22a**	381.43	106.16	0	6	8	67.63	3.21	1.12e-02	Moderately soluble	High
**22b**	397.42	108.18	1	7	8	87.86	2.89	1.60e-02	Moderately soluble	High
**22c**	441.48	119.14	0	8	10	86.09	3.23	9.11e-03	Moderately soluble	High
**22d**	411.45	112.65	0	7	9	76.86	3.24	1.02e-02	Moderately soluble	High
**22e**	369.39	99.62	0	6	7	58.40	3.55	8.81e-03	Moderately soluble	High
**22f**	351.40	99.67	0	5	7	58.40	3.24	1.20e-02	Moderately soluble	High
**22g**	396.40	108.49	0	7	8	104.22	2.69	1.19e-02	Moderately soluble	High
**23a**	380.44	108.36	0	5	8	54.74	3.43	1.12e-02	Moderately soluble	High
**23b**	396.44	110.39	1	6	8	74.97	3.09	1.59e-02	Moderately soluble	High
**23c**	440.49	121.35	0	7	10	73.20	3.41	9.22e-03	Moderately soluble	High
**23d**	410.46	114.85	0	6	9	63.97	3.43	1.02e-02	Moderately soluble	High
**23e**	368.40	101.83	0	5	7	45.51	3.76	8.78e-03	Moderately soluble	High
**23f**	384.86	106.88	0	4	7	45.51	3.98	3.36e-03	Moderately soluble	High
**26a**	397.42	105.91	1	7	6	87.86	2.67	2.30e-02	Moderately soluble	High
**26b**	441.48	116.87	0	8	8	86.09	2.97	1.31e-02	Moderately soluble	High
**26c**	411.45	110.38	0	7	7	76.86	2.95	1.47e-02	Moderately soluble	High
**26d**	369.39	97.35	0	6	5	58.40	3.33	1.27e-02	Moderately soluble	High
**26e**	351.40	97.39	0	5	5	58.40	3.03	1.73e-02	Moderately soluble	High
**27a**	396.44	108.11	1	6	6	74.97	2.85	2.29e-02	Moderately soluble	High
**27b**	440.49	119.07	0	7	8	73.20	3.16	1.33e-02	Moderately soluble	High
**27c**	410.46	112.58	0	6	7	63.97	3.18	1.47e-02	Moderately soluble	High
**27d**	424.49	117.39	0	6	8	63.97	3.48	8.73e-03	Moderately soluble	High
**27e**	368.40	99.56	0	5	5	45.51	3.55	1.26e-02	Moderately soluble	High
**27f**	350.41	99.60	0	4	5	45.51	3.24	1.72e-02	Moderately soluble	High
**27g**	395.41	108.42	0	6	6	91.33	2.65	1.71e-02	Moderately soluble	High
**27h**	425.43	114.91	0	7	7	100.56	2.62	1.55e-02	Moderately soluble	High
**27i**	366.41	101.62	1	5	5	65.74	2.83	2.48e-02	Moderately soluble	High
**30**	466.53	131.28	0	7	11	73.20	3.96	3.22e-03	Moderately soluble	High

**33a**	486.35	130.12	0	1	4	17.82	5.88	1.43e-05	Poorly soluble	Low
**33b**	361.44	115.20	0	2	4	30.71	4.49	7.26e-04	Moderately soluble	High

[a]SwissADME http://www.swissadme.ch; [b]according to the white of the BOILED-Egg; [c]Average of all five predictions; [d]Topological method to predict Water Solubility included in SwissADME using an implementation of the ESOL model; [e] Solubility class: Log S scale: Insoluble < -10 < Poorly < -6 < Moderately < -4 < Soluble < -2 Very < 0 < Highly

Table S3: Pharmacokinetic interaction estimations with P-gp and CYP 450 isozymes of (*E*)-1-(3-(4-methoxyphenyl)-1-(3,4,5-trimethoxyphenyl)allyl)-1*H*-1,2,4-triazoles and related compounds)[a]

Cpd No.	BBB permeable[b]	P-gp substrate	CYP1A2 Inhibitor	CYP2C19 inhibitor	CYP2C9 inhibitor	CYP2D6 Inhibitor	CYP3A4 Inhibitor	Skin permeation Coefficient (cm/s)	Abbott Bioavailability Score[c]
**22a**	Yes	No	Yes	Yes	Yes	No	Yes	-5.91	0.55
**22b**	No	No	No	Yes	No	No	Yes	-6.26	0.55
**22c**	No	No	No	Yes	Yes	No	No	-6.32	0.55
**22d**	Yes	No	Yes	Yes	Yes	No	No	-6.12	0.55
**22e**	Yes	No	Yes	Yes	Yes	No	No	-5.75	0.55
**22f**	Yes	No	Yes	Yes	Yes	No	No	-5.71	0.55
**22g**	No	No	Yes	Yes	Yes	No	Yes	-6.11	0.55
**23a**	Yes	No	Yes	Yes	Yes	Yes	Yes	-5.90	0.55
**23b**	Yes	No	Yes	Yes	Yes	Yes	No	-6.25	0.55
**23c**	Yes	No	No	Yes	Yes	No	No	-6.31	0.55
**23d**	Yes	No	Yes	Yes	Yes	No	Yes	-6.11	0.55
**23e**	Yes	No	Yes	Yes	Yes	Yes	Yes	-5.74	0.55
**23f**	Yes	No	Yes	Yes	Yes	Yes	Yes	-5.46	0.55
**26a**	No	No	Yes	No	Yes	Yes	Yes	-6.59	0.55
**26b**	No	No	No	No	Yes	Yes	Yes	-6.64	0.55
**26c**	Yes	No	Yes	No	Yes	Yes	Yes	-6.45	0.55
**26d**	Yes	No	No	Yes	Yes	Yes	Yes	-6.08	0.55
**26e**	Yes	No	Yes	Yes	Yes	Yes	Yes	-6.04	0.55
**27a**	Yes	Yes	No	No	Yes	Yes	Yes	-6.57	0.55
**27b**	Yes	No	No	No	Yes	Yes	Yes	-6.64	0.55
**27c**	Yes	No	No	No	Yes	Yes	Yes	-6.43	0.55
**27d**	Yes	Yes	No	No	Yes	Yes	Yes	-6.26	0.55
**27e**	Yes	Yes	Yes	Yes	Yes	Yes	Yes	-6.06	0.55
**27f**	Yes	Yes	Yes	Yes	Yes	Yes	Yes	-6.02	0.55
**27g**	No	No	Yes	Yes	Yes	Yes	Yes	-6.42	0.55
**27h**	No	No	Yes	Yes	Yes	Yes	Yes	-6.62	0.55
**27i**	Yes	Yes	Yes	Yes	No	Yes	Yes	-6.37	0.55
**30**	No	No	No	Yes	Yes	No	No	-6.01	0.55
**33a**	No	Yes	Yes	Yes	Yes	No	No	-4.42	0.55
**33b**	Yes	Yes	Yes	Yes	Yes	No	Yes	-4.88	0.55

[a] SwissADME: a free web tool to evaluate pharmacokinetics, drug-likeness and medicinal chemistry friendliness of small molecules http://www.swissadme.ch; [b]According to the yolk of the BOILED-Egg; [c]The bioavailability score is the parameter which predict the probability of more than 10% oral bioavailability in rat based on PSA (potential surface area) and Lipinski rule-of-five. This parameter falls on four classes of probabilities (11%, 17%, 56% or 85%) and allows to filter off the molecules with cell-permeability issues

Table S4: Drug-likeness of representative compounds assessed via lead-likeness and reactivity/toxicity filters for (*E*)-1-(3-(4-methoxyphenyl)-1-(3,4,5-trimethoxyphenyl)allyl)-1*H*-1,2,4-triazoles and related compounds [a ,d]

Cpd No.	Lipinski	Ghose	Veber	Egan	Muegge	PAINS[b]	Brenk[c]	Lead-likeness	Synthetic accessibility
**22a**	Yes; 0 violation	Yes	Yes	Yes	Yes	0 alerts	3 alerts	No; 3 violations: MW>350, Rotors>7, XLOGP3>3.5	3.85
**22b**	Yes; 0 violation	Yes	Yes	Yes	Yes	0 alerts	0 alerts	No; 2 violations: MW>350, Rotors>7	3.93
**22c**	Yes; 0 violation	Yes	Yes	Yes	Yes	0 alerts	0 alerts	No; 3 violations: MW>350, Rotors>7, XLOGP3>3.5	4.20
**22d**	Yes; 0 violation	Yes	Yes	Yes	Yes	0 alerts	0 alerts	No; 3 violations: MW>350, Rotors>7, XLOGP3>3.5	4.04
**22e**	Yes; 0 violation	Yes	Yes	Yes	Yes	0 alerts	0 alerts	No; 2 violations: MW>350, XLOGP3>3.5	3.69
**22f**	Yes; 0 violation	Yes	Yes	Yes	Yes	0 alerts	0 alerts	No; 2 violations: MW>350, XLOGP3>3.5	3.70
**22g**	Yes; 0 violation	Yes	Yes	Yes	Yes	0 alerts	1 alert: nitro group	No; 3 violations: MW>350, Rotors>7, XLOGP3>3.5	3.75
**23a**	Yes; 0 violation	Yes	Yes	Yes	Yes	0 alerts	0 alerts	No; 3 violations: MW>350, Rotors>7, XLOGP3>3.5	3.71
**23b**	Yes; 0 violation	Yes	Yes	Yes	Yes	0 alerts	0 alerts	No; 2 violations: MW>350, Rotors>7	3.80
**23c**	Yes; 0 violation	Yes	Yes	Yes	Yes	0 alerts	0 alerts	No; 3 violations: MW>350, Rotors>7, XLOGP3>3.5	4.07
**23d**	Yes; 0 violation	Yes	Yes	Yes	Yes	0 alerts	0 alerts	No; 3 violations: MW>350, Rotors>7, XLOGP3>3.5	3.92
**23e**	Yes; 0 violation	Yes	Yes	Yes	Yes	0 alerts	0 alerts	No; 2 violations: MW>350, XLOGP3>3.5	3.56
**23f**	Yes; 0 violation	Yes	Yes	Yes	Yes	0 alerts	0 alerts	No; 2 violations: MW>350, XLOGP3>3.5	3.57
**26a**	Yes; 0 violation	Yes	Yes	Yes	Yes	0 alerts	0 alerts	No; 1 violation: MW>350	4.01

**26b**	Yes; 0 violation	Yes	Yes	Yes	Yes	0 alerts	0 alerts	No; 2 violations: MW>350, Rotors>7	4.27
**26c**	Yes; 0 violation	Yes	Yes	Yes	Yes	0 alerts	0 alerts	No; 1 violation: MW>350	4.12
**26d**	Yes; 0 violation	Yes	Yes	Yes	Yes	0 alerts	0 alerts	No; 1 violation: MW>350	3.81
**26e**	Yes; 0 violation	Yes	Yes	Yes	Yes	0 alerts	0 alerts	No; 1 violation: MW>350	3.82
**27a**	Yes; 0 violation	Yes	Yes	Yes	Yes	0 alerts	0 alerts	No; 1 violation: MW>350	3.90
**27b**	Yes; 0 violation	Yes	Yes	Yes	Yes	0 alerts	0 alerts	No; 2 violations: MW>350, Rotors>7	4.17
**27c**	Yes; 0 violation	Yes	Yes	Yes	Yes	0 alerts	0 alerts	No; 1 violation: MW>350	4.01
**27d**	Yes; 0 violation	Yes	Yes	Yes	Yes	0 alerts	0 alerts	No; 3 violations: MW>350, Rotors>7, XLOGP3>3.5	4.15
**27e**	Yes; 0 violation	Yes	Yes	Yes	Yes	0 alerts	0 alerts	No; 1 violation: MW>350	3.71
**27f**	Yes; 0 violation	Yes	Yes	Yes	Yes	0 alerts	0 alerts	No; 1 violation: MW>350	3.71
**27g**	Yes; 0 violation	Yes	Yes	Yes	Yes	0 alerts	1 alert: nitro group	No; 1 violation: MW>350	3.82
**27h**	Yes; 0 violation	Yes	Yes	Yes	Yes	0 alerts	1 alert: nitro group	No; 1 violation: MW>350	4.04
**27i**	Yes; 0 violation	Yes	Yes	Yes	Yes	0 alerts	0 alerts	No; 1 violation: MW>350	3.75
**30**	Yes; 0 violation	No; 1 violation: MR>130	No; 1 violation: Rotors >10	Yes	Yes	0 alerts	0 alerts	No; 3 violations: MW>350, Rotors>7, XLOGP3>3.5	4.16
**33a**	Yes; 1 violation: MLOGP>4.15	No; 3 violations: MW>480, WLOGP>5.6, MR>130	Yes	No; 1 violation: WLOGP>5.88	No; 1 violation: XLOGP3 >5	0 alert	2 alerts: iodine, polycyclic aromatic hydrocarbon 2	No; 2 violations: MW>350, XLOGP3>3.5	3.72
**33b**	Yes; 0 violation	No; 1 violation: WLOGP>5.6	Yes	Yes	No; 1 violation: XLOGP3 >5	0 alert	1 alert: polycyclic aromatic hydrocarbon 2	No; 2 violations: MW>350, XLOGP3>3.5	3.48

[a]SwissADME http://www.swissadme.ch [b]Pan Assay Interference Structures: (PAINS); [c]Brenk Structural Alert e.g. Michael acceptor, more than 2 esters; nitro group, oxygen-nitrogen single bond, phthalimide, alkyl halide; [d]Additional druglikeness assessment methods including Ghose, Veber and Muegge filters together with medicinal chemistry structure alert assessments PAINS (pan assay interference compounds) and Brenk filters were used to identify

compounds which are potentially toxic, chemically reactive, metabolically unstable compounds or having poor pharmacokinetics.

References

1. Ducki, S.; Mackenzie, G.; Greedy, B.; Armitage, S.; Chabert, J.F.; Bennett, E.; Nettles, J.; Snyder, J.P.; Lawrence, N.J. Combretastatin-like chalcones as inhibitors of microtubule polymerisation. Part 2: Structure-based discovery of alpha-aryl chalcones. *Bioorg Med Chem* **2009**, *17*, 7711-7722.
2. Ducki, S.; Forrest, R.; Hadfield, J.A.; Kendall, A.; Lawrence, N.J.; McGown, A.T.; Rennison, D. Potent antimitotic and cell growth inhibitory properties of substituted chalcones. *Bioorg Med Chem Lett* **1998**, *8*, 1051-1056.
3. Prakasham, A.P.; Saxena, A.K.; Luqman, S.; Chanda, D.; Kaur, T.; Gupta, A.; Yadav, D.K.; Chanotiya, C.S.; Shanker, K.; Khan, F.*, et al.* Synthesis and anticancer activity of 2-benzylidene indanones through inhibiting tubulin polymerization. *Bioorg Med Chem* **2012**, *20*, 3049-3057.
4. Edwards, M.L.; Stemerick, D.M.; Sunkara, P.S. Chalcones: A new class of antimitotic agents. *J Med Chem* **1990**, *33*, 1948-1954.
5. Rao, Y.K.; Fang, S.H.; Tzeng, Y.M. Synthesis and biological evaluation of 3',4',5'-trimethoxychalcone analogues as inhibitors of nitric oxide production and tumor cell proliferation. *Bioorg Med Chem* **2009**, *17*, 7909-7914.
6. La Regina, G.; Bai, R.; Coluccia, A.; Famiglini, V.; Pelliccia, S.; Passacantilli, S.; Mazzoccoli, C.; Ruggieri, V.; Sisinni, L.; Bolognesi, A.*, et al.* New pyrrole derivatives with potent tubulin polymerization inhibiting activity as anticancer agents including hedgehog-dependent cancer. *J Med Chem* **2014**, *57*, 6531-6552.
7. Pathak, V.; Ahmad, I.; Kahlon, A.K.; Hasanain, M.; Sharma, S.; Srivastava, K.K.; Sarkar, J.; Shankar, K.; Sharma, A.; Gupta, A. Syntheses of 2-methoxyestradiol and eugenol template based diarylpropenes as non-steroidal anticancer agents. *RSC Adv.* **2014**, *4*, 35171-35185.
8. Lawrence, N.J.; Armitage, E.S.M.; Greedy, B.; Cook, D.; Ducki, S.; McGown, A.T. . The synthesis of indanones related to combretastatin a-4 via microwave-assisted nazarov cyclization of chalcones. . *Tetrahedron Lett.* **2006**, *47*, 1637-1640. .
9. Chang, M.Y.; Tsai, C.Y.;Wu, M.H. Nbs-mediated cyclization of trans-cinnamic alcohols. . *Tetrahedron* **2013**, *69*, 6364-6370.
10. Lawrence, N.J; Hadfield, J.A; McGown, A.T.; Butler, J.; Ducki, S.; Rennison, D.; Woo, M. Combretastatin A4 derivatives having antineoplastic activity. *WO2003040077, Paterson Institute for Cancer Research, United Kingdom, University of Manchester Institute of Science and Technology, Manchester, UK,* **2003**.
